# Genome-wide association study of endometrial cancer in E2C2

**DOI:** 10.1007/s00439-013-1369-1

**Published:** 2013-10-06

**Authors:** Immaculata De Vivo, Jennifer Prescott, Veronica Wendy Setiawan, Sara H. Olson, Nicolas Wentzensen, The Australian National Endometrial Cancer Study Group, John Attia, Amanda Black, Louise Brinton, Chu Chen, Constance Chen, Linda S. Cook, Marta Crous-Bou, Jennifer Doherty, Alison M. Dunning, Douglas F. Easton, Christine M. Friedenreich, Montserrat Garcia-Closas, Mia M. Gaudet, Christopher Haiman, Susan E. Hankinson, Patricia Hartge, Brian E. Henderson, Elizabeth Holliday, Pamela L. Horn-Ross, David J. Hunter, Loic Le Marchand, Xiaolin Liang, Jolanta Lissowska, Jirong Long, Lingeng Lu, Anthony M. Magliocco, Mark McEvoy, Tracy A. O’Mara, Irene Orlow, Jodie N. Painter, Loreall Pooler, Radhai Rastogi, Timothy R. Rebbeck, Harvey Risch, Carlotta Sacerdote, Fredrick Schumacher, Rodney J. Scott, Xin Sheng, Xiao-ou Shu, Amanda B. Spurdle, Deborah Thompson, David VanDen Berg, Noel S. Weiss, Lucy Xia, Yong-Bing Xiang, Hannah P. Yang, Herbert Yu, Wei Zheng, Stephen Chanock, Peter Kraft

**Affiliations:** 1 Channing Division of Network Medicine, Department of Medicine, Brigham and Women’s Hospital and Harvard Medical School, Boston, MA 02115 USA; 2 Department of Epidemiology, Harvard School of Public Health, Boston, MA USA; 3 University of Southern California, Los Angeles, CA USA; 4 Memorial Sloan-Kettering Cancer Center, New York, NY USA; 5 Division of Cancer Epidemiology and Genetics, National Cancer Institute, Bethesda, MD USA; 6 Division of Genetics and Population Health, Queensland Institute of Medical Research, Brisbane, Australia; 7 Centre for Clinical Epidemiology and Biostatistics, School of Medicine and Public Health, University of Newcastle, Newcastle, Australia; 8 Department of General Medicine, Hunter Medical Research Institute, John Hunter Hospital, University of Newcastle, Newcastle, Australia; 9 Fred Hutchinson Cancer Research Center, Seattle, WA USA; 10 University of New Mexico, Albuquerque, NM USA; 11 Division of Cancer Care, Department of Population Health Research, Alberta Health Services, Calgary, AB Canada; 12 Geisel School of Medicine, Dartmouth College, Lebanon, NH USA; 13 Strangeways Research Laboratory, Centre for Cancer Genetic Epidemiology (CCGE), University of Cambridge, Cambridge, UK; 14 Strangeways Research Laboratory, Department of Oncology, University of Cambridge, Cambridge, UK; 15 Strangeways Research Laboratory, Department of Public Health and Primary Care, University of Cambridge, Cambridge, UK; 16 Epidemiology Research Program, American Cancer Society, Atlanta, GA USA; 17 Division of Biostatistics and Epidemiology, University of Massachusetts, Amherst, MA USA; 18 Centre for Information Based Medicine and the School of Medicine and Public Health, University of Newcastle, Newcastle, Australia; 19 Hunter Medical Research Institute, Hunter Area Pathology Service, John Hunter Hospital, Newcastle, Australia; 20 Cancer Prevention Institute of California, Fremont, CA USA; 21 University of Hawaii Cancer Center, Honolulu, HI USA; 22 Department of Cancer Epidemiology and Prevention, M Sklodowska-Curie Cancer Center and Institute of Oncology, Warsaw, Poland; 23 Department of Medicine, Vanderbilt Epidemiology Center, Vanderbilt-Ingram Cancer Center, Vanderbilt University Medical Center, Nashville, TN USA; 24 Yale University School of Public Health, New Haven, CT USA; 25 H Lee Moffitt Cancer Centre and Research Institute, Tampa, FL USA; 26 Center for Clinical Epidemiology and Biostatistics, University of Pennsylvania School of Medicine, Philadelphia, PA USA; 27 Center for Cancer Prevention (CPO-Piemonte), Turin, Italy; 28 Human Genetic Foundation (HuGeF), Turin, Italy; 29 Centre for Information Based Medicine and the School of Biomedical Science and Pharmacy, University of Newcastle, Newcastle, Australia; 30 University of Washington, Seattle, WA USA; 31 Department of Epidemiology, Shanghai Cancer Institute, Shanghai, China

## Abstract

Endometrial cancer (EC), a neoplasm of the uterine epithelial lining, is the most common gynecological malignancy in developed countries and the fourth most common cancer among US women. Women with a family history of EC have an increased risk for the disease, suggesting that inherited genetic factors play a role. We conducted a two-stage genome-wide association study of Type I EC. Stage 1 included 5,472 women (2,695 cases and 2,777 controls) of European ancestry from seven studies. We selected independent single-nucleotide polymorphisms (SNPs) that displayed the most significant associations with EC in Stage 1 for replication among 17,948 women (4,382 cases and 13,566 controls) in a multiethnic population (African America, Asian, Latina, Hawaiian and European ancestry), from nine studies. Although no novel variants reached genome-wide significance, we replicated previously identified associations with genetic markers near the *HNF1B* locus. Our findings suggest that larger studies with specific tumor classification are necessary to identify novel genetic polymorphisms associated with EC susceptibility.

**Electronic supplementary material:**

The online version of this article (doi:10.1007/s00439-013-1369-1) contains supplementary material, which is available to authorized users.

## Introduction

Endometrial cancer (EC), a neoplasm of the uterine epithelial lining, is the most common gynecological malignancy in developed countries and the fourth most common cancer among US women (www.cancer.org 2013). This disease primarily affects postmenopausal women and is more common in women of European ancestry. In the USA in 2013, an estimated 49,560 women may develop EC and 8,190 may die from the disease, a case fatality similar to that of breast cancer. The estimated lifetime risk of women developing the disease in the USA is 1 in 38 (www.cancer.org 2013). EC is categorized into two distinct subtypes based on histologic and clinical characteristics. Type I ECs, the most common in women of European ancestry (80–90 %), are mostly endometrioid adenocarcinomas (EA). The remaining 10–20 % of ECs are Type II, which predominantly consist of serous and clear cell carcinomas.

EC risk is strongly increased by a Western lifestyle, with up to tenfold higher incidence rates in Western, industrialized countries than in Asia or rural Africa (Pisani et al. 1993). Major risk factors include obesity and use of postmenopausal estrogen-only hormone therapy (ET). Excess body weight has been associated with a two to fivefold increase in EC risk in both pre- and postmenopausal women, and has been estimated to account for about 40–50 % of EC incidence in affluent societies (Bergstrom et al. 2001). Epidemiological evidence also suggests increased risks in association with early age of menarche, late age of menopause, nulliparity and infertility. Furthermore, women with a family history of EC have their risk increased by nearly twofold (Gruber and Thompson 1996; Lucenteforte et al. 2009) and an even greater risk in rare family cancer syndromes such as Lynch syndrome (also termed hereditary nonpolyposis colorectal cancer, HNPCC) (Papadopoulos et al. 1994; Nicolaides et al. 1994; Risinger et al. 1993), suggesting that inherited genetic factors increase susceptibility to EC. Though these studies support an inherited genetic component to risk (Vasen et al. 1994; Schildkraut et al. 1989; Gruber and Thompson 1996; Seger et al. 2011), twin studies suggest that the familial aggregation in risk may be mostly due to shared environmental factors and not shared genetics (Lichtenstein et al. 2000).

The predominant mechanistic hypothesis describing Type I endometrial carcinogenesis is known as the “unopposed estrogen” hypothesis (Key and Pike 1988). This theory states that EC risk is increased among women who have high circulating levels of bioavailable estrogens and low levels of progesterone, so that the mitogenic effect of estrogens is insufficiently counterbalanced by the opposing effect of progesterone. The unopposed estrogen hypothesis is supported by observations that the use of ET (Herrinton and Weiss 1993; Persson et al. 1989) and of Oracon (a sequential oral contraceptive (OC) characterized by an unusually high ratio of estrogenic to progestogenic activity) (Weiss and Sayvetz 1980) greatly increase EC risk, while use of combined OCs (i.e., containing progestins as well as estrogen throughout the treatment period) is associated with a reduced risk (Henderson et al. 1983). A further observation that led to the unopposed estrogen hypothesis is that mitotic rates of endometrial tissue are higher during the follicular phase of the menstrual cycle, when progesterone levels are low and the uterine lining undergoes proliferation, than during the luteal phase (Ferenczy et al. 1979). Progesterone counteracts the growth-stimulatory effects of estrogen by inducing glandular and stromal differentiation (Clarke and Sutherland 1990; Ace and Okulicz 1995) and endometrial hyperplasia can be reversed by progestin therapy (Ehrlich et al. 1981). Many of the genes in the sex steroid hormone metabolism pathway have served as “candidates” in search of polymorphic variants that predispose to EC. Although some studies suggest that SNPs in these genes, for example, the *CYP19A1* (aromatase) gene, are associated with EC risk (Setiawan et al. 2009), very little of the genetic risk can be explained by these SNPs.

To this end, efforts have been undertaken to identify genes involved in EC causation. Recently, two genome-wide association studies (GWAS) of EC have been conducted (Spurdle et al. 2011; Long et al. 2012). However, only one study identified a novel genome-wide significant association (*P* = 7.1 × 10^−10^) with a susceptibility marker located at 17q12 (rs4430796), near the HNF1 homeobox B (*HNF1B*) gene, in relation to EC. Though originally identified in women of European ancestry, this locus has been replicated in other ethnicities (Setiawan et al. 2012). This marker has also been associated with prostate cancer (Thomas et al. 2008), diabetes (Winckler et al. 2007; Gudmundsson et al. 2007) and certain subtypes of ovarian cancer (Shen et al. 2013). In search of additional common genetic variants, we conducted a two-stage GWAS of EC among women participating in studies that are part of the Epidemiology of Endometrial Cancer Consortium (E2C2, details in Supplementary Table 1).

## Results

We conducted a GWAS within the E2C2 to identify genetic loci that predispose to EC. Details on the 15 participating studies are provided in Supplementary Table 1. The discovery phase of the GWAS (Stage 1) was conducted among women of European ancestry and was restricted to Type 1 EC, the most common subtype accounting for 80–90 % of all cases in women of European descent. Seven participating studies, including four cohort [California Teacher’s Study (CTS), Nurses’ Health Study (NHS), Multiethnic Cohort (MEC), Prostate, Lung, Colorectal, and Ovarian Cancer Screening Trial (PLCO)] and three case–control studies [Connecticut Endometrial Cancer (CONN), Fred Hutchinson Cancer Research Center (FHCRC), Polish Endometrial Cancer Study (PECS)], were genotyped in Stage 1 (2,695 cases, 2,777 controls). Study-specific population characteristics are summarized in Table 1. The mean age at diagnosis for cases in Stage 1 ranged from 59.6 in FHCRC to 67.7 in PLCO.

**Table 1:** Studies participating in the genome-wide association study (GWAS)

Study	Study acronym	Study type^a^	Platform used for genotyping	Number of cases	Number of controls	Location	Mean age at diagnosis years (cases)	Total
DISCOVERY–STAGE 1 Europeans only								
California Teacher’s Study	CTS	COHORT	Illumina Omniexpress	295	285	USA (CA)	65.3	580
Connecticut Endometrial Cancer Study	CONN	CACO	Illumina Omniexpress	482	571	USA (CT)	60.6	1,053
Fred Hutchinson Cancer Research Center	FHCRC	CACO	Illumina Omniexpress	640	693	USA (WA)	59.6	1,333
Multiethnic Cohort Study	MEC	COHORT	Illumina Omniexpress	90	199	USA (CA, HI)	65.0	289
Nurses’ Health Study	NHS	COHORT	Illumina Omniexpress	385	348	USA (11 STATES)	62.3	733
Polish Endometrial Case Control Study	PECS	CACO	Illumina Human 660 W	424	558	POLAND	61.1	982
Prostate, lung, colorectal and ovarian cancer screening trial	PLCO	COHORT	Illumina Omniexpress (cases) Illumina Omni 2.5 (controls)	379	123	USA (SEVERAL)	67.7	502
REPLICATION –FAST-TRACK (9 SNPS) AND STAGE 2 (1818 SNPS) All ethnicities								
Alberta Health Services	AHS	CACO	Taqman/Illumina Exome12v custom content	487	911	CANADA	58.5	1,398
Estrogen, Diet, Genetics and Endometrial Cancer	EDGE	CACO	Taqman/Illumina Exome12v custom content content	259	239	USA (NJ)	60.7	498
Fred Hutchinson Cancer Research Center	FHCRC	CACO	Taqman/Illumina Exome12v custom content	48	53	USA (WA)	60.3	101
Multiethnic Cohort Study	MEC	COHORT	Taqman/Illumina Exome12v custom	320	647	USA	65.5	967
REPLICATION –FAST-TRACK (9 SNPS) AND STAGE 2 (1818 SNPS) All ethnicities								
Cancer Prevention Study II	CPS II	COHORT	Taqman	496	497	USA (21 STATES)	69.0	993
Turin	TURIN	CACO	Taqman	265	274	ITALY	61.7	539
The Women’s Insights and Shared Experiences Study	WISE	CACO	Taqman	386	736	USA (NJ, PA)	62.9	1,122
Australian National Endometrial Case Study/Studies of Epidemiology Risk Factors in Cancer Heredity (Europeans)	ANECS/SEARCH	CACO	Illumina Infinium 610 k	1,287	8,273			9,560
Australian National Endometrial Cancer Study	ANECS	CACO (cases only)	Illumina Infinium 610 k	606	–	AUSTRALIA	61.2	
Queensland Institute of Medical Research	QIMR	COHORT (controls only)	Illumina Infinium 610 k	–	1,846	AUSTRALIA	NA	
Hunter Community Study	HCS	COHORT (controls only)	Illumina Infinium 610 k	–	1,237	AUSTRALIA	NA	
Study of epidemiology and risk factors in cancer heredity	SEARCH	CACO (cases only)	Illumina Infinium 610 k	681	–	BRITAIN	58.1	
Wellcome Trust Case–Control Consortium	WTCCC	CACO (controls only)	Illumina Infinium	–	5,190	BRITAIN	NA	
Shanghai Endometrial Cancer Genetics Study (Chinese)	SECGS	CACO	Affymetrix 6.0	834	1,936	CHINA	54.8	2,770

Number of cases and controls included in the final analysis

Specific ethnicities are reported in Supplementary Table 5

^a^CACO–Case–control study

**Fig. 1:**
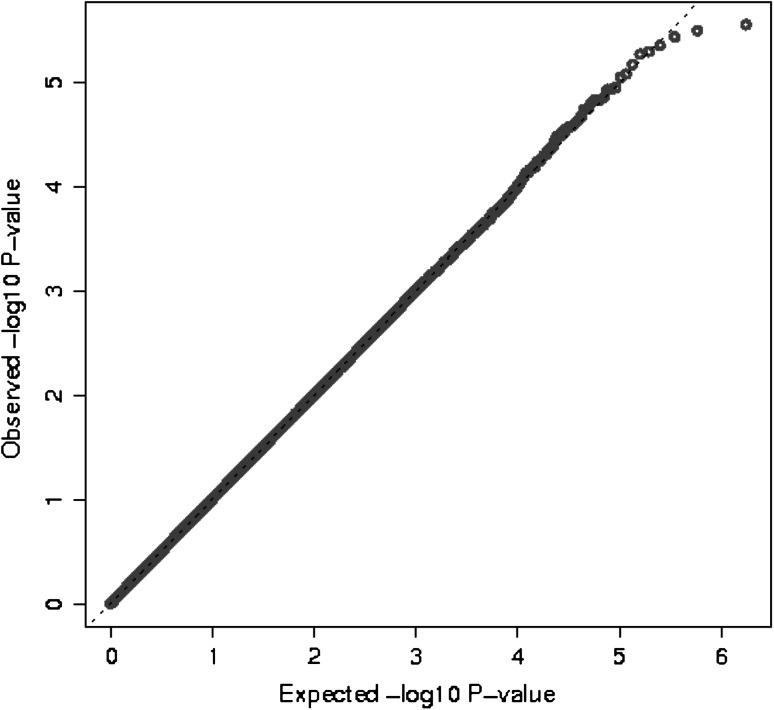
Log quantile–quantile (Q–Q) plot. The observed –log10 *P* values (Y-axis) of 873,935 SNPs from a meta-analysis of seven studies included in the discovery phase of the endometrial cancer GWAS adjusted for the principal components of genetic variation plotted against the expected –log10 quantile (X-axis). The genomic control lambda is 1.008. Imputed *P* values are represented by the* dashed line*

**Fig. 2:**
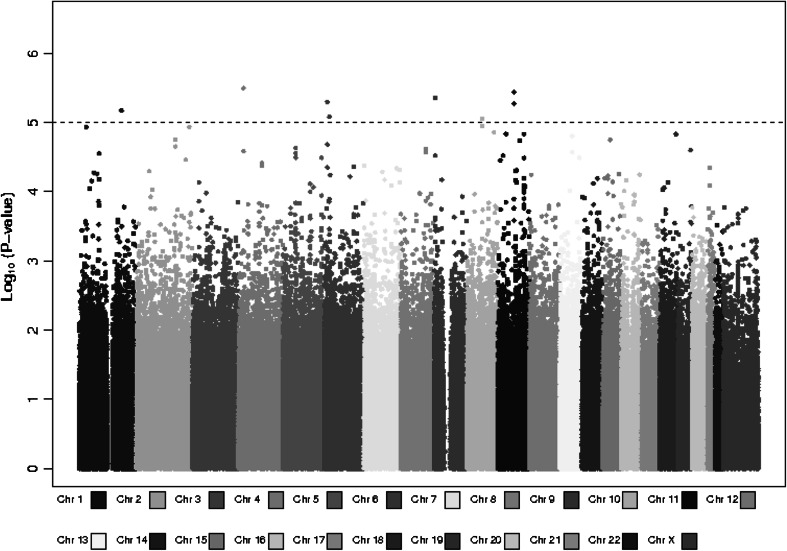
Manhattan plot of the association results. The –log10 *P* values from the meta-analysis of seven studies in the discovery phase of the endometrial cancer GWAS adjusted for principal components of genetic variation plotted against chromosomal base pair position. Chromosomes are* color coded*

After quality control metrics were applied (see methods), over 524K-genotyped SNPs remained in each study for a combined total of unique 873K SNPs for analysis. The genomic control lambda for the study was 1.008, indicating little evidence of population substructure, relatedness or differential genotyping between cases and controls (Fig. 1). No SNP association reached genome-wide significance (*P* < 5 × 10^−8^) (Fig. 2). In particular, we did not replicate rs1202524 (*P* = 0.39), a reported EC susceptibility locus in Asian women (Long et al. 2012), in our Stage 1 population of women of European ancestry.

Among SNPs associated with the smallest ranked *P* values, rs9344 and rs1352075 at the 11q13.3 locus caught our attention because of significant associations between this locus and breast cancer (Turnbull et al. 2010) and renal cancer (Purdue et al. 2011) in prior GWAS. Thus, we initially pursued a fast-track replication for seven SNPs independently associated with EC (*r* ^2^ < 0.2) at *P* < 1 × 10^−5^ from Stage 1, as well as two *HNF1B* SNPs (rs4430796 and rs11651755) identified by Spurdle et al. (Spurdle et al. 2011) (Supplementary Table 2). The fast-track replication was conducted in a multiethnic sample of 2,294 cases and 3,395 controls from two cohorts [MEC and the Prevention Study II (CPSII) Nutrition cohort] and five case–control studies [the Alberta Health Services (AHS) study, FHCRC, Estrogen, Diet, Genetics, and Endometrial Cancer (EDGE) study, Turin, and Women’s Insights and Shared Experiences (WISE)] (Table 1). Among women of European ancestry, we replicated EC associations with SNPs at the *HNF1B* locus (*P* < 0.005), but did not replicate any of the other seven SNPs (Supplementary Table 2a): the lowest *P* value for the seven SNPs in fast-track replication was 0.18. No statistically significant associations were observed when we examined among other ethnicities (Supplementary Table 2b).

We selected 2,129 SNPs with *P* < 0.0037 in Stage 1 for follow-up in a subset of the fast-track replication studies and two previously conducted GWAS (ANECS/SEARCH and SECGS) for Stage 2 (Supplementary Tables 3 and 4). DNA samples from a multiethnic sample of women in AHS, FHCRC, MEC and EDGE (Supplementary Table 5) were genotyped for 1,818 of these SNPs as custom content on Illumina’s Human Exome 12v1 chip; the remaining SNPs failed design or quality control. After pooled analysis, no SNP association reached genome-wide significance in women of European ancestry or in women of multiple ethnicities combined, either among Type I EC cases (Table 2) or among those with endometrioid subtype (Table 3). In addition, we further adjusted for BMI; results did not change qualitatively (data not shown).

**Table 2:** Results for all Type I endometrial cancer cases and controls in Stage 1 GWAS and Stage 2 replication (Illumina Exome 12v with custom content plus in silico validation) where *P* < 1 × 10^−4^

SNP	Chr	Position (bp)	Gene/locus	Coded allele	MAF	GWAS		Replication		GWAS + Replication	
						OR (95 % CI)	*P*	OR (95 % CI)	*P*	OR (95 % CI)	*P*
rs9459805	6	167336151	RNASET2, LOC100131869	A	0.19^a^	1.61 (1.26–2.06)	1.47E−04	1.15 (1.06–1.25)	7.61E−04	1.19 (1.10–1.29)	1.11E−05
rs4812563	20	40579609		T	0.39^a^	1.16 (1.06–1.26)	1.13E−03	1.10 (1.03–1.17)	2.09E−03	1.12 (1.06–1.17)	1.21E−05
rs7178187	15	99683251	SYNM, TTC23	A	0.47	1.13 (1.04–1.23)	3.46E−03	1.10 (1.04–1.17)	1.12E−03	1.11 (1.06–1.17)	1.37E−05
rs1453082	18	55936104	NEDD4L	A	0.14^a^	0.83 (0.74–0.93)	1.19E−03	0.86 (0.78–0.96)	4.90E−03	0.85 (0.78–0.91)	2.03E−05
rs1995971	7	79930551		T	0.31^a^	0.75 (0.62–0.90)	2.55E−03	0.89 (0.83–0.95)	6.38E−04	0.87 (0.82–0.93)	2.50E−05
rs11679180	2	229642422		A	0.24	1.22 (1.12–1.34)	1.16E−05	1.07 (1.00–1.15)	6.14E−02	1.13 (1.06–1.19)	3.02E−05
rs5940807	X	89552827	LOC100131981	T	0.47^a^	1.16 (1.07–1.27)	5.84E−04	1.14 (1.02–1.28)	2.25E−02	1.15 (1.08–1.24)	3.76E−05
rs12536378	7	154058474	DPP6	T	0.39^a^	1.15 (1.06–1.24)	6.33E−04	1.11 (1.02–1.21)	1.97E−02	1.13 (1.07–1.20)	4.15E−05
rs10887831	10	90268918	RNLS	T	0.16	0.80 (0.71–0.90)	4.12E−04	0.90 (0.82–0.98)	1.26E−02	0.86 (0.80–0.93)	4.93E−05
rs6468613	8	88009165	CNBD1	T	0.49^a^	0.88 (0.81–0.95)	9.92E−04	0.89 (0.82–0.98)	1.70E−02	0.88 (0.83–0.94)	4.95E−05
rs12778749	10	127846669	ADAM12	T	0.30^a^	1.14 (1.05–1.24)	1.70E−03	1.09 (1.02–1.16)	7.43E−03	1.11 (1.06–1.17)	5.43E−05
rs2891316	9	28587994	LINGO2	T	0.18	0.83 (0.74–0.93)	1.30E−03	0.89 (0.82–0.97)	9.39E−03	0.87 (0.81–0.93)	5.76E−05
rs12514742	5	35188476	PRLR	T	0.12	0.82 (0.73–0.92)	8.88E−04	0.82 (0.69–0.97)	2.37E−02	0.82 (0.74–0.90)	5.78E−05
rs1777220	6	126022602	LOC643623	A	0.38	0.85 (0.78–0.92)	4.34E−05	0.94 (0.85–1.03)	2.02E−01	0.88 (0.83–0.94)	7.06E−05
rs12521272	5	35190327	PRLR	A	0.19^a^	1.18 (1.07–1.30)	9.69E−04	1.10 (1.02–1.20)	1.54E−02	1.13 (1.07–1.21)	7.37E−05
rs6724138	2	55314518		A	0.20	0.79 (0.71–0.89)	5.06E−05	0.92 (0.85–1.00)	5.09E−02	0.88 (0.82–0.94)	7.82E−05
rs1692108	12	55095662		T	0.05^a^	0.72 (0.58–0.88)	1.56E−03	0.80 (0.67–0.95)	1.28E−02	0.76 (0.67–0.87)	7.88E−05

Stage 1 sample size: 5,472 (2,695 cases and 2,777 controls)

Stage 2 sample size: 15,294 (3,235 cases and 12,059 controls)

Contributing studies: CTS, CONN, FHCRC, MEC, NHS, PECS, PLCO, AHS, EDGE, ANECS/SEARCH, SECGS

MAF based on GWAS (^a^indicates MAF is for non-coded allele)

**Table 3:** Results for SNPs shown in Table 2, endometrioid cases only and controls in Stage 1 GWAS and Stage 2 replication

SNP	Chr	Position (bp)	Gene/locus	Coded allele	MAF	GWAS		Replication		GWAS + Replication	
						OR (95 % CI)	*P*	OR (95 % CI)	*P*	OR (95 % CI)	*P*
rs9459805	6	167336151	RNASET2, LOC100131869	A	0.19^a^	1.64 (1.23–2.19)	8.20E−04	1.18 (1.07–1.29)	7.07E−04	1.21 (1.11–1.33)	2.10E−05
rs4812563	20	40579609		T	0.40^a^	1.13 (1.02–1.25)	1.84E−02	1.11 (1.04–1.20)	3.52E−03	1.12 (1.05–1.19)	1.81E−04
rs7178187	15	99683251	SYNM, TTC23	A	0.47	1.16 (1.05–1.28)	2.84E−03	1.11 (1.03–1.18)	4.84E−03	1.12 (1.06–1.19)	5.55E−05
rs1453082	18	55936104	NEDD4L	A	0.14^a^	0.80 (0.70–0.91)	9.38E−04	0.88 (0.79–0.98)	1.85E−02	0.85 (0.78–0.92)	8.91E−05
rs1995971	7	79930551		T	0.31^a^	0.67 (0.54–0.83)	2.26E−04	0.90 (0.84–0.97)	6.86E−03	0.87 (0.81–0.94)	1.64E−04
rs11679180	2	229642422		A	0.24	1.23 (1.11–1.36)	6.18E−05	1.10 (1.01–1.19)	2.92E−02	1.15 (1.08–1.22)	2.37E−05
rs5940807	X	89552827	LOC100131981	T	0.48^a^	1.18 (1.07–1.31)	6.90E−04	1.08 (0.96–1.23)	2.08E−01	1.15 (1.06–1.24)	5.45E−04
rs12536378	7	154058474	DPP6	T	0.41^a^	1.10 (1.01–1.21)	2.85E−02	1.16 (1.02–1.31)	2.67E−02	1.12 (1.04–1.21)	2.19E−03
rs10887831	10	90268918	RNLS	T	0.16	0.81 (0.71–0.94)	5.31E−03	0.88 (0.80–0.98)	2.18E−02	0.86 (0.79–0.94)	4.77E−04
rs6468613	8	88009165	CNBD1	T	0.49^a^	0.89 (0.82–0.98)	1.16E−02	0.86 (0.76–0.97)	1.79E−02	0.88 (0.82–0.95)	6.19E−04
rs12778749	10	127846669	ADAM12	T	0.31^a^	1.17 (1.06–1.28)	1.88E−03	1.13 (1.05–1.22)	1.51E−03	1.14 (1.08–1.21)	1.01E−05
rs2891316	9	28587994	LINGO2	T	0.19	0.84 (0.74–0.96)	8.65E−03	0.89 (0.81–0.97)	1.14E−02	0.87 (0.81–0.94)	3.32E−04
rs12514742	5	35188476	PRLR	T	0.13	0.82 (0.72–0.94)	3.86E−03	0.79 (0.64–0.97)	2.44E−02	0.81 (0.72–0.91)	2.63E−04
rs1777220	6	126022602	LOC643623	A	0.39	0.86 (0.79–0.94)	1.31E−03	0.86 (0.75–0.98)	2.38E−02	0.86 (0.80–0.93)	8.55E−05
rs12521272	5	35190327	PRLR	A	0.19^a^	1.18 (1.05–1.32)	4.11E−03	1.14 (1.04–1.24)	3.98E−03	1.15 (1.08–1.24)	5.50E−05
rs6724138	2	55314518		A	0.20	0.82 (0.72–0.94)	3.37E−03	0.92 (0.84–1.00)	5.65E−02	0.88 (0.82–0.95)	1.16E−03
rs1692108	12	55095662		T	0.04^a^	0.73 (0.58–0.91)	6.56E−03	0.77 (0.64–0.92)	4.83E−03	0.75 (0.65–0.87)	9.53E−05

Stage 1 sample size: 4,335 (1,558 cases and 2,777 controls)

Stage 2 sample size: 2,616 (766 cases and 1,850 controls)

Contributing studies: CTS, CONN, FHCRC, MEC, PECS, PLCO, AHS, EDGE, ANECS/SEARCH

MAF based on GWAS (^a^indicates MAF is for non-coded allele). MAF may differ slightly from Table 2 because of different population used

## Discussion

Our present study reports results from a new independent GWAS of EC based on a total of 7,077 cases and 16,343 controls from the E2C2 (Table 1). We did not identify any novel loci associated with EC that reached genome-wide significance (*p* < 5 × 10^−8^).

In a joint analysis of the GWAS and replication populations, the variant most significantly associated with EC was rs9459805 on chromosome 6 at the *RNASET2* locus (OR = 1.19, 95 % CI 1.10–1.29; *P* = 1.11 × 10^−5^, Table 2). Of potential interest, two variants suggestively associated with EC (rs12514742, joint *P* = 5.78 × 10^−5^; rs12521272, joint *P* = 7.37 × 10^−5^) are located at the prolactin receptor (*PRLR*) gene locus on chromosome 5. Circulating levels of prolactin, a polypeptide hormone involved in numerous physiological processes including reproduction, are higher among EC patients compared to healthy controls (Levina et al. 2009; Yurkovetsky et al. 2007; Kanat-Pektas et al. 2010), and increased PRLR expression has been noted for endometrial tumors compared to non-cancerous endometrial tissue. Prolactin signaling via *PRLR* has also been shown to potentiate proliferation and inhibit chemotherapy-induced apoptosis of EC cell lines (Levina et al. 2009). Additional studies in independent populations are required to confirm whether variants at the *PRLR* locus influence EC risk.

To date, only one locus associated with EC at the genome-wide significance level has been identified by GWAS (Spurdle et al. 2011). Located within the *HNF1B* gene on chromosome 17, the common variant most significantly associated with EC (rs4430796; OR per G allele = 0.84, 95 % CI 0.79–0.89; *P* = 7.1 × 10^−10^) in the GWAS by Spurdle et al. (2011) was nominally associated with EC in our discovery (Stage 1) population in the expected direction (OR per G allele = 0.92, *P* = 0.03; Supplementary Table 2a). This effect estimate is consistent with a winner’s-curse adjustment of the original GWAS effect estimate, which also yields a per G allele OR of 0.92 (Zhong and Prentice 2008). Further genotyping within fast-track replication studies confirmed the association of the rs4430796 G allele with reduced EC risk among women of European ancestry (joint OR = 0.90, 95 % CI 0.85–0.96; *P* = 5.2 × 10^−4^) with no evidence of heterogeneity between studies (*P* = 0.50). In the earlier GWAS by Spurdle et al., the discovery phase was restricted to patients with the endometrioid histologic subtype of EC. Additionally restricting the replication stage to cases with endometrioid histology (~77 % of cases) slightly strengthened the association between rs4430796 and EC risk (joint OR = 0.82, 95 % CI 0.77–0.87; *P* = 4.3 × 10^−11^) in the study by Spurdle et al. (2011).

Our GWAS study included all EC cases diagnosed with Type 1 tumors, a group consisting of the following histologic subtypes: endometrioid adenocarcinoma (ICD-O-3 codes 8380, 8381, 8382, 8383), adenocarcinoma tubular (8210, 8211), papillary adenocarcinoma (8260, 8262, 8263), adenocarcinoma with squamous metaplasia (8570), mucinous adenocarcinoma (8480, 8481) and adenocarcinoma NOS (8140) (Kim et al. 2008). Even though the endometrioid adenocarcinoma subtypes represent the majority of Type 1 tumors (60 %) (Robboy et al. 2009), the inclusion of the less common Type 1 histologic subtypes may have introduced sufficient heterogeneity to reduce power to detect genome-wide significant associations. However, when we restricted our analysis to Stage 1 and Stage 2 cases with known endometrioid histology, the overall association of rs4430796 with EC risk remained the same, while the significance weakened most likely due to a loss of power from the reduced sample size. This is consistent with results from the PAGE study, which found that *HNF1B* may be a general susceptibility locus for EC, as risk associated with rs4430796 [G] was similar for Type 1 and Type 2 tumors (Setiawan et al. 2012). Most of the suggestive SNP associations in our study (Table 2) were slightly weakened when the analysis was restricted to cases with known endometrioid histology (Table 3).

Endometrial cancer is part of Lynch syndrome, which is attributable to the inheritance of rare, highly penetrant mutations in DNA mismatch repair genes (Nicolaides et al. 1994; Peltomaki et al. 1993; Aaltonen et al. 1993). The lifetime risk of EC among women with HNPCC is 50–60 %, whereas that of the general population is 2–3 %(Seger et al. 2011). Women with this inherited predisposition to endometrial neoplasm tend to develop the disease 15 years earlier than the general population (Vasen et al. 1994). Studies on estimates of heritability for EC suggested a high genetic component for younger women (Schildkraut et al. 1989; Gruber and Thompson 1996; Parslov et al. 2000). In addition, a record linkage study in Utah (Seger et al. 2011) indicated that there was considerable clustering of EC in families, even accounting for obesity. On the other hand, a twin study of sporadic cancers (i.e., not attributable to family cancer syndromes), which account for 98 % of EC cases, suggests a low genetic contribution (Lichtenstein et al. 2000).

Based on the results of this study and the previous GWAS in European ancestry women (Spurdle et al. 2011), it is unlikely that there exist any common variants with large effects on the risk of EC, although there may be many markers with smaller effects. For example, the probability that at least one of these GWAS would identify a genome-wide significant association with a marker that had a per-allele odds ratio of 1.2 and a risk allele frequency of 0.30 is over 80 %. Conversely, the power of this study to identify a marker like rs4430796 with a per-allele odds ratio of 1.08 and risk allele frequency of 0.52 is 5 %; the power of the Spurdle et al. GWAS was under 1 %. This suggests that *circa* 18 additional markers with *HNF1B*-like effects on EC exist, but have not yet been identified due to low power (Park et al. 2010). Consequently, a GWAS with 12,000 cases and 24,000 controls—triple the sample size of the two European ancestry GWAS conducted to date—should identify three or more markers with *HNF1B*-like effect sizes with 85 % probability, as well as other markers with smaller effects. We caution that these projections are based on only one known GWAS-identified risk marker; we cannot rule out a larger number of *HNF1B*-like risk markers and can say little about markers with subtler effects.

In conclusion, we did not identify any novel loci associated with EC susceptibility. Taken together, a low inherited genetic component, tumor heterogeneity and the small expected effects of genetic variants could explain the apparent lack of association. Therefore, larger studies with specific tumor classification (Kandoth et al. 2013) are necessary to identify novel genetic polymorphisms associated with EC susceptibility.

## Materials and methods

### Study participants

Participating studies are described in Table 1 and comprise a total of 7,077 EC cases and 16,343 controls from 15 studies (ten case–control and five cohort, which were analyzed as nested case–control). Cases in Stage 1 were diagnosed with Type I EC. In cohort studies, controls were cancer free at the time of case diagnosis. In case–control studies, controls had not had hysterectomies. The cohort studies were analyzed as nested case–control studies. Cases of European descent from CTS, CONN, FHRC, MEC, NHS and PLCO were scanned using Illumina Omniexpress. PLCO controls were scanned using Illumina Omni 2.5 and the PECS cases and controls were scanned using Illumina Human 660 W. With the exception of PLCO, all controls were matched to cases on age within each study site. Each participating study obtained informed consent from study participants and approval from its institutional review board (IRB) for this study and obtained IRB certification permitting data sharing in accordance with the NIH Policy for Sharing of Data Obtained in NIH Supported or Conducted Genome-Wide Association studies (GWAS).

Participating studies in Stage 2 are described in Table 1. We did not restrict to European ancestry in this stage; a multiethnic population was included (Supplementary Table 5), although we also conducted sensitivity analyses restricted to women of European ancestry. We conducted two replications, a fast track, in which nine SNPs were genotyped in all studies except ANECS, SEARCH and SECGS using the Taqman assay. Stage 2 was conducted using the Illumina’s Human Exome 12v1 chip with custom content in the following studies: AHS, FHCRC, MEC and EDGE.

### GWAS Genotyping

DNA was isolated from peripheral blood following the manufacturer’s recommended protocol. Genotyping was performed at two centers. At least 625 ng of each DNA sample from NHS, CONN, MEC, CTS and FHCRC was sent to USC for genotyping using the HumanOmniExpress BeadChips (Illumina Inc, San Diego, CA). The BeadChips were run on an Illumina iScan system using the Infinium HD Assay Super Automated Protocol. The GenomeStudio Genotyping (GT) Module (Illumina Inc, San Diego, CA) was used for data normalization and genotype calling. The following studies were genotyped at the Core Genotyping Facility (CGF), at the National Cancer Institute; PLCO cases were genotyped using the Illumina Omni Express chip, PECS controls were previously genotyped on the Illumina Human 660 W chip and PLCO controls were genotyped on the Omni 2.5 M chip.

### Replication genotyping

Fast-track replication was performed at the Dana Farber/Harvard Cancer Center High-Throughput Genotyping Core on the ABI PRISM 7900HT Sequence Detection System (Applied Biosystems, Foster City, CA) according to the manufacturer’s instructions. TaqMan^®^ assays were ordered using either Assays-on-Demand or using the ABI Assays-By-Design service. All Stage 2 replication samples were genotyped using Illumina Exome 12v with custom content (*N* = 1818 SNPs) (Table 1).

### Genome-wide association analysis

In total, 5,806 women with genotypes were available for Stage 1 analysis. To minimize bias due to population stratification, we used ~7,600 ancestry informative markers to identify and exclude women with <80 % European ancestry (*N* = 146). An additional four participants were excluded based on a self-report as being of non-European descent. We also identified four unexpected inter-study duplicates (all EC cases) and removed one subject from each unexpected duplicate pair. Because the scan was based on women of European descent with Type I EC, 180 cases of Type II EC were excluded for a final sample size of 5,472 (2,695 cases, 2,777 controls) women eligible for Stage 1. After filtering SNPs with completion rates <90 %, minor allele frequencies <1 %, and out of Hardy–Weinberg equilibrium (*P* < 0.0001) we had >524K genotyped SNPs in each Stage 1 study for a combined total of >873K unique SNPs across all studies. Concordance between known duplicates was >99.9 %.

We applied similar filters to the newly genotyped Stage 2 samples. Four pairs of unexpected duplicates (eight total samples) and 30 samples with <90 % SNP completion rate were removed. One genetically male sample and seven samples that did not cluster with other samples from their self-reported ancestry group were also excluded, leaving 2,975 samples for analysis. SNPs with <90 % completion rate were removed from analysis, as were SNPs that showed deviation from HWE at *P* < 10^−5^ in any ethnic group.

Genotyping procedures, quality control and analysis procedures for the ANECS/SEARCH and SECGS GWAS have been reported previously (Spurdle et al. 2011; Long et al. 2012).

In all analyses, genotypes were coded log additively (0, 1, 2 copies of the minor allele) and logistic regression was used to model associations. Stage 1 analyses were adjusted for study and the first two principal components. Analyses of the newly genotyped Stage 2 data (i.e., all Stage 2 studies except ANECS/SEARCH or SECGS) were adjusted for study and the first four principal components. Principal components for Stage 1 were calculated using ~7,600 independent markers (Yu et al. 2008); principal components for Stage 2 were calculated using 47,097 common SNPs on the exome chip. Of the 1,818 SNPs selected for replication in Stage 2, 1,371 loci included additional in silico data from two previously reported GWAS (Spurdle et al. 2011; Long et al. 2012) in a total of 2,121 cases and 10,209 controls from SEARCH/ANECS and SECGS studies. Study populations were analyzed separately and results combined using fixed effects meta-analysis. Association analyses of SNPs selected for fast-track replication were conducted in SAS Version 9.2 (SAS Institute, Cary, NC, USA). All other analyses were performed using PLINK software package (v 1.07, October 2009).

### Electronic supplementary material

Below is the link to the electronic supplementary material.

Supplementary material 1 (DOCX 31 kb)

Supplementary material 2 (PDF 214 kb)

Supplementary material 3 (DOCX 284 kb)

Supplementary material 4 (DOCX 216 kb)

Supplementary material 5 (DOCX 16 kb)

## Supplementary acknowledgments

The **Nurses’ Health Study (NHS)** is supported by the NCI, NIH Grants Number 1R01 CA134958, 2R01 CA082838, P01 CA087969, and R01 CA49449. The authors would like to thank the participants and staff of the Nurses’ Health Study for their valuable contributions as well as the following state cancer registries for their help: AL, AZ, AR, CA, CO, CT, DE, FL, GA, ID, IL, IN, IA, KY, LA, ME, MD, MA, MI, NE, NH, NJ, NY, NC, ND, OH, OK, OR, PA, RI, SC, TN, TX, VA, WA, WY. In addition, this study was approved by the Connecticut Department of Public Health (DPH) Human Investigations Committee. Certain data used in this publication were obtained from the DPH. The authors assume full responsibility for analyses and interpretation of these data. The authors would also like to thank Channing Division of Network Medicine, Department of Medicine, Brigham and Women’s Hospital and Harvard Medical School. Finally, the authors would also like to acknowledge Pati Soule and Hardeep Ranu for their laboratory assistance.

The **Cancer Prevention Study II (CPS-II) cohort** is supported by the American Cancer Society (ACS) and by NCI, NIH Grant Number 2R01 CA082838. The CPS-II would like to thank the CPS-II participants and Study Management Group for their invaluable contributions to this research. The authors would also like to acknowledge the contribution to this study from central cancer registries supported through the Centers for Disease Control and Prevention National Program of Cancer Registries, and cancer registries supported by the National Cancer Institute Surveillance Epidemiology and End Results program.

The **Multiethnic Cohort Study (MEC)** is supported by the NCI, NHI Grants Number CA54281, CA128008 and 2R01 CA082838.

The **Fred Hutchinson Cancer Research Center (FHCRC)** is supported by NCI, NIH Grant Number 2R01 CA082838, NIH RO1 CA105212, RO1 CA 87538, RO1 CA75977, RO3 CA80636, NO1 HD23166, R35 CA39779, KO5 CA92002 and funds from the Fred Hutchinson Cancer Research Center.

The **Women’s Insights and Shared Experiences (WISE)** is supported by the NCI, NIH Grants Number P01-CA77596 and 2R01 CA082838.

The **Connecticut Endometrial Cancer Study** is supported by NCI, NIH Grant Number 2R01 CA082838.

The **Shanghai Endometrial Cancer Genetic Study (SECGS)** was supported by the US PHS grants R01 CA98285, R01 CA064277, and R01 CA90899, NCI, NIH Grant Number 2R01 CA082838 as well as institutional funds from the Vanderbilt University. The SECGS would like to thank research staff and participants of the Shanghai Endometrial Cancer Study and Shanghai Breast Cancer Study for their contributions in the study.

The **EDGE Study (Estrogen, Diet, Genetics, and Endometrial Cancer)** is supported by NCI, NIH Grants Number 2R01 CA082838, R01CA83918 (Olson PI) and P30-CA008748 (Cancer Center Support Grant).

The **Australian National Endometrial Cancer Study (ANECS)** recruitment was supported by project grants from the National Health and Medical Research Council of Australia (ID#339435), The Cancer Council Queensland (ID#4196615) and Cancer Council Tasmania (ID#403031 and ID#457636). The **Studies of Epidemiology and Risk factors in Cancer Heredity (SEARCH)** recruitment was funded by a program grant from Cancer Research UK [C490/A10124]. Case genotyping was supported by the National Health and Medical Research Council (ID#552402). Control data was generated by the Wellcome Trust Case Control Consortium (WTCCC), and a full list of the investigators who contributed to the generation of the data is available from the WTCCC website. We acknowledge use of DNA from the British 1958 Birth Cohort collection, funded by the Medical Research Council grant G0000934 and the Wellcome Trust grant 068545/Z/02. Funding for this project was provided by the Wellcome Trust under award 085475. Recruitment of the QIMR controls was supported by the National Health and Medical Research Council of Australia (NHMRC). The University of Newcastle, the Gladys M Brawn Senior Research Fellowship scheme, The Vincent Fairfax Family Foundation, the Hunter Medical Research Institute and the Hunter Area Pathology Service all contributed towards the costs of establishing the Hunter Community Study. A.B.S. is supported by the National Health and Medical Research Council (NHMRC) Fellowship Scheme. D.F.E. is a Principal Research Fellow of Cancer Research UK. A.M.D is supported by the Joseph Mitchell Trust. ANECS, SEARCH, QIMR and the HCS thank the many individuals who participated in the research studies, Penny Webb, Kaltin Ferguson, and the ANECS research team, Nick Martin, Grant Montgomery, Dale Nyholt and Anjali Henders for access to GWAS data from QIMR controls, Paul Pharoah for his role in implementing and supporting the SEARCH study, the Eastern Cancer Registration and Information Centre and the SEARCH research team, the numerous institutions and their staff who supported recruitment, and acknowledge the contribution of our clinical and scientific collaborators and their staff. See http://www.anecs.org.au/ for full listing of the ANECS Group and other contributors to ANECS. SEARCH collaborators include Caroline Baynes, Don Conroy, Bridget Curzon, Patricia Harrington, Sue Irvine, Clare Jordan, Craig Luccarini, Rebecca Mayes, Hannah Munday, Barbara Perkins, Radka Platte, Anabel Simpson, Anne Stafford and Judy West. QIMR thanks Margie Wright, Lisa Bowdler, Sara Smith, Megan Campbell and Scott Gordon for control sample collection and data processing.

The **California Teachers Study (CTS)** is supported by NCI, NIH Grant Number 2R01 CA082838, R01 CA91019 and R01 CA77398, and contract 97-10500 from the California Breast Cancer Research Fund.

The **Polish Endometrial Cancer Study (PECS)** is supported by the Intramural Research Program of the NCI.

The **Prostate, Lung, Colorectal, and Ovarian Cancer Screening Trial (PLCO)** is supported by the Extramural and the Intramural Research Programs of the NCI.

The **Alberta Health Services Study (AHS)** was supported by operating grants obtained from the National Cancer Institute of Canada with funds from the Canadian Cancer Society and the Canadian Institute for Health Research. The AHS is also supported by NCI, NIH Grant Number 2R01 CA082838. Dr Christine Friedenreich is supported by career awards from Alberta Innovates-Health Solutions and the Alberta Cancer Foundation through the Weekend to End Women’s Cancers Breast Cancer Chair. Dr Linda Cook was supported through the Canada Research Chairs program.

The **Turin endometrial cancer case control study** was supported by the Italian Association for Research on Cancer (AIRC) and Ricerca Finalizzata Regione Piemonte.
