# Supplementary material for: Genome-wide association study of endometrial cancer in E2C2
Source: Hum Genet. 2013 Oct 6;133(2):211–24. doi: 10.1007/s00439-013-1369-1 (PMC3898362; doi:10.1007/s00439-013-1369-1)
Supplement: Supplementary file 1 — Supplementary material 1 (DOCX 31 kb) [file 439_2013_1369_MOESM1_ESM.docx]

**Supplementary Methods: Table 1**

**Case Control**

**Connecticut:** The Connecticut Endometrial Cancer Study is a population-based case-control study conducted between December 2004 and March 2009. The study enrolled 668 incident cases and 674 population controls. The cases were newly diagnosed endometrial cancer patients aged between 35 and 80 years, and were identified from 28 hospitals in Connecticut through Rapid Case Ascertainment, a Yale Cancer Center shared resource. The control subjects were selected through random digit dialing, and were frequency matched to cases on age. Each subject enrolled underwent an in-person interview. A structured questionnaire was used to elicit information on demographic features, menstrual and reproductive history, use of exogenous hormones, medical history, family history of cancer, dietary habit and physical activity. Majority of the study participants (92%) provided biological samples, and most of the specimens were blood samples. For those who did not give blood, saliva samples were collected using the Oragene kits.

**FHCRC:** The Fred Hutchinson Cancer Research Center (FHCRC) endometrial cancer studies are comprised of three population-based case-control studies conducted in King, Pierce, and Snohomish counties of western Washington State, and include 882 cases and 1,022 controls. All of the studies had similar study protocols and questionnaires, and have been described in detail previously [Doherty JA et al, Cancer Epidemiol Biomarkers Prev 20, 1873-82, 2011]. Study participants were ages 50-74 years. Cases included women who were diagnosed with invasive endometrial adenocarcinoma in 1994-1995, 1997-1999, and 2003-2005. Eligible control women had intact uteri and were identified using random-digit dialing or through random selection from Health Care Financing Administration data files. In-person interviews ascertained height, weight at different ages, reproductive, contraceptive, and menstrual history, family history of cancer, history of selected chronic conditions, and history of contraceptive and non-contraceptive hormone use. For the earlier two studies (1994-1999), 81.1% of eligible cases (n=472) and 71.5% of eligible controls (n=664) were interviewed, and of these, 81.1% of cases and 67.7% of controls provided a blood sample. For the latter study period (2003-2005), 70.0% of cases (n=410) and 84.1% of controls (n=356) were interviewed and 97.8% of cases and 97.5% of controls provided either a blood or buccal sample.

**Polish:** The Polish Endometrial Cancer Study (PECS) included women, ages 20 to 74 years, who were newly diagnosed with histologically or cytologically confirmed invasive endometrial cancer in Warsaw or Lodz during 2001-2003. Cases were identified through a rapid identification system at participating hospitals and through the local cancer registries. Controls who had no prior uterine cancer or hysterectomy were selected through the Polish Electronic System (PESEL), a database of all Polish residents during case accrual, and frequency-matched to cases on age and location. A total of 550 (79.3%) of 695 eligible cases and 1,925 (67.7%) of 2,843 eligible controls completed an in-person interview.

**Alberta Health Services**: The Case-Control Study of Endometrial Cancer and Lifetime Physical Activity is a component of a multidisciplinary research program on endometrial cancer in Alberta [E-CAP]. Lifestyle exposures were determined retrospectively through an in-person interview and completion of a diet history questionnaire. Cases were women 30-79 years of age with incident, histologically-confirmed, invasive, primary endometrial cancer diagnosed in Alberta between September 12, 2002 and February 15, 2006 and consecutively identified directly from pathology reports and the Alberta Cancer Registry (ACR). Controls were women over the age of 30 years who were residents of Alberta, never diagnosed with endometrial cancer and without a hysterectomy or endometrial ablation. Controls were population-based, randomly identified from the general population via probability sampling using a bank of randomly selected telephone numbers and subsequently contacted through random-digit dialing (RDD). 549 cases and 1034 controls participated in the study and of those, 526 cases and 977 controls provided a blood sample. The in-person interview was done using cognitive interviewing methods that included memory probes to aide in recall. Collection of a blood sample occurred either pre-hysterectomy or post-hysterectomy for cases and occurred post-interview for controls at participating laboratories throughout Alberta.

**SECGS:** The Shanghai Endometrial Cancer Genetic Study (SECGS) includes 834 endometrial cancer cases who were recruited to the Shanghai Endometrial Cancer Study (SECS) and 1936 controls who were recruited to the Shanghai Breast Cancer Study (SBCS). As described in detail elsewhere, both SECS and SBCS are two population-based case-control studies that were conducted in parallel in Shanghai during same period using an identical study protocol (Xu WH *et al.*, Cancer Epidemiol Biomarkers Prev 2009; Zheng W *et al.,* Nat Genet 2009). Briefly, 1199 women aged between 30 and 69 with newly diagnosed with EC between 1997 and 2003 were identified through the population-based tumor registry and recruited to the SECS (response rate 83%). The SBCS controls were randomly selected from the general population using the Shanghai Resident Registry with response rate of 74%. Women with prior hysterectomies were not eligible for inclusion in this study. Participants completed a detailed in-person interview at the time of enrollment and provided a blood or buccal cell sample. Case and control genotype data for stage 2 SNPs, or for correlated SNPs with R2>0.8, were extracted from existing Affymetrix 6.0 genome-wide scan data (Long *et al.,* Cancer Epidemiol Biomarkers Prev 2012).

**EDGE:** The EDGE Study (Estrogen, Diet, Genetics, and Endometrial Cancer) is a population-based case-control study conducted in six counties in northern New Jersey. Cases were eligible if they were age 21 and over, newly diagnosed with endometrial cancer between July 1, 2001 and June 30, 2005, and spoke English or Spanish. Controls were age 21 and over, resided in these counties, and had not had a hysterectomy. They were located by random digit dialing, use of lists of CMS subscribers, and area sampling. There were a total of 469 cases and 467 controls, including 417 cases and 402 controls who provided a buccal sample. Pathology reports and slides were obtained for cases in the study and reviewed by the study pathologist.

**ANECS/SEARCH**: The Australian National Endometrial Cancer Study (ANECS) is an Australian population-based case-control family study of cancer of the uterine corpus (Spurdle et al., Nat Genet 2011). Women aged 18-79 years, registered on the Electoral Roll and newly diagnosed with primary cancer of the endometrium between July 2005 and December 2007 were identified through major hospitals nationally and also from state-based cancer registries. Case participation rate was 63%. All ANECS participants completed a detailed questionnaire providing clinical and epidemiological information including BMI and ethnicity**.**

The Studies of Epidemiology and Risk factors in Cancer Heredity (SEARCH) is an ongoing UK population-based study with cases ascertained through the Eastern Cancer Registration and Information Center (http://www.ecric.org.uk). All women diagnosed with endometrial cancer between the ages of 18–69 years (average age diagnosis 58 years) from 31 July 2001 to 1 September 2007 were eligible for inclusion. Approximately 54% of eligible patients had enrolled in the study at the time genotyping took place. Women taking part in the study were asked to provide a 20 ml blood sample for DNA analysis and to complete a comprehensive epidemiological questionnaire.

Genome-wide genotyping of the ANECS and SEARCH cases was performed using an Illumina Infinium 610K array and called using the Illuminus algorithm. Genotypes were available for 1317 cases with endometrial cancer. Samples were excluded as follows: probable Turner’s syndrome or male sex based on genotypes for markers on the X and Y chromosomes (n=4); call rate <95% (n=15); heterozygosity outside 5 standard deviations from the mean (n=7); probable sibling pairs identified as close relatives by identity-by-state probabilities >0.85 (n=3); >15% non-European ancestry estimated from identity-by-state scores (n=1), leaving a total of 1287 cases (606 from ANECS and 681 from SEARCH). The duplicate concordance was 99.998%.

Controls were genotyped as part of the Wellcome Trust Case Control Consortium (WTCCC2) (Wellcome Trust Case Control Consortium, Nature 2007). These controls are drawn from two sources: 2922 controls from the 1958 Birth Cohort (1958BC), a population-based study in the United Kingdom of individuals born in 1 week in 1958 (Power *et al*., Int J Epidemiol 2006); and 2737 controls identified through the UK National Blood Service (NBS) (Wellcome Trust Case Control Consortium, Nature 2007). The analyses were based on 2694 1958BC and 2496 NBS controls.

The Queensland Institute of Medical Research (**QIMR**) control sample is a subsection of subjects recruited as part of the Brisbane Adolescent Twin Study (McGregor *et al.* Genet Epidemiol 1999; Zhu *et al.,* Am J Hum Genet 1999). Twins were recruited from schools in Brisbane, Australia and surrounding areas of southeast Queensland and were examined close to their 12th birthday. Blood was obtained from all twins and most parents. Parents were asked the ancestry of all eight great-grandparents of the twins. More than 95% of great-grandparents were identified as being of northern European ancestry, mainly from Britain and Ireland. Analysis used genotype data from parents and siblings only, extracted from an existing Illumina 610K BeadChip genome-wide association scan (Painter *et al.*, Nat Genet 2011) and recalled using the Illuminus algorithm. After standard QC steps (as for the case data) 1846 QIMR controls were included in the analysis.

The Hunter Community Study (**HCS**) is a population-based cohort study consisting of men and women aged 55-85 years of age in Newcastle, New South Wales, Australia (McEvoy *et al.,* Int J Epidemiol 2010). Participants were randomly selected from the NSW State electoral roll (listing on the electoral roll is compulsory in Australia) and contacted between December 2004 and December 2007. Non-English speaking persons and those living in a residential aged-care facility were ineligible for participation in the study. Participants were asked to complete five self-report questionnaires as well as attend the HCS data collection centre so clinical measures could be obtained. In total, 44.5% of eligible controls agreed to participate in this study. Genotype data for this study were extracted from an existing Illumina 610K BeadChip genome-wide association study scan and recalled using the Illuminus algorithm. After standard QC steps (as for the case data) 1237 HCS controls were included in the analysis.

**WISE:** The Women’s Insights and Shared Experiences (WISE) study is a population-based case‑control study of 616 case patients with incident endometrial cancer and 1583 frequency-matched control subjects selected from the community by random digit dialing. Full details of the study design have been previously reported^78^. The source population for this study was the contiguous nine-county region around Philadelphia. Eligible cases were African American or White women residing in these counties who were newly diagnosed with endometrial cancer between July 1, 1999, and June 30, 2002, and aged 50-79 years at the time of diagnosis. The case patients were identified through active surveillance at 65 hospitals that were open during this period. Control subjects were selected from residents of the same geographic regions as the case patients, and ascertained by random-digit dialing. Telephone interviews and medical records abstractions were used to collect data. We have collected DNA from buccal cells collected using swabs for all cases and controls.

**TURIN STUDY**: The Turin case-control study recruited 297 women (age 40-74) with newly diagnosed cancer of the endometrium (histologically confirmed) treated at the Turin gynaecological hospital and living in Piedmont region (North-Western Region of Italy). Two groups of controls were recruited: i) a random sample of the female population (age 40-74) of the EPIC Turin study recruited in 1998-1999 (N=98); ii) a hospital sample of women (age 40-74) treated at the same hospital than cases for minor affections not related to diet or to hormonal status (N=209). Both control groups included only residents in Piedmont Region and not hysterectomized women. After informed consent, blood samples were collected from cases and hospital controls. Blood samples from EPIC controls were drawn at the moment of recruitment in the EPIC study and stored in liquid nitrogen. Questionnaires of EPIC Italy (Lifestyle and Food Frequency Questionnaires) were used to collect information about contraceptive and reproductive history, use of hormone replacement therapy, physical activity, history of previous and/or current illnesses, medical and surgical treatment and hospitalization, education, socioeconomic status, tobacco smoke. Work, recreational, household and vigorous physical activity were assessed as part of the standardized lifestyle questionnaire. In addition to lifestyle data, the FFQ systematically estimating average portion sizes, and containing up to 260 food items was used.

**Cohort**

**MEC:** Between 1993 and 1996, participants entered the Hawaii/Los Angeles Multiethnic Cohort Study (MEC) after completing and returning a baseline questionnaire. The MEC consists of 215,251 men and women (age 45-75 years at baseline) from Hawaii (Japanese, Whites and Native Hawaiians) and California (African-Americans and Latinos). Incident cancer cases are currently ascertained through population-based SEER tumor registries in California and Hawaii. Blood samples have been collected from cases with breast, prostate, colorectal and endometrial cancer and controls, and we are currently in the process of establishing a biological specimen bank that will include prospectively collected biospecimens from approximately 80,000 cohort participants.

**CTS:** The California Teachers Study (CTS) is a large prospective cohort study of 133,479 women, age 21 or older at baseline (Bernstein *et al.,* Cancer Causes and Control 2002). Participants were active (within two years) or retired California public school teachers and administrators when the cohort was established in 1995-96. As long as participants reside in California (93% at baseline with only 9% outmigration by 2013) they remain in active follow-up for cancer outcomes via linkage with the statewide population-based California Cancer Registry. Follow-up is also conducted through annual contact with participants and linkages with the National Change of Address, National Death Index, and other databases. As part of a nested case-control study of endometrial cancer, we obtained blood specimens from 395 endometrial cancer cases, age 50 or older, diagnosed between joining the cohort and December 31, 2004, and 654 controls, frequency-matched to cases on 5-yr age group, race/ethnicity, and broad geographic region within California. Blood was obtained from 272 cases and 445 controls and buccal cells from 123 cases and 209 controls.

**NHS:** The Nurses’ Health Study (NHS) consists of 121,700 women enrolled in 1976 aged 30-55 years of age at baseline. In 1989-90, 32,826 participants provided a blood sample, and in 2000-1, 32,883 participants provided a buccal cell sample. Cancer follow-up is conducted by personal mailings and searches of the National Death Index and is estimated to be greater than 90%. All participants diagnosed with cancer are asked for permission to review medical records to confirm the diagnoses and obtain additional information on tumor histology, staging, and other characteristics.

**PLCO Cohort:** The Prostate, Lung, Colorectal, and Ovarian Cancer Screening Trial (PLCO), coordinated by NCI in 10 U.S. centers, enrolled during 1993 - 2001 approximately 77,000 men and 78,000 women, aged 55-74, in a randomized, two-arm trial to determine if screening reduced mortality from these cancers. Sequential blood samples, including plasma, serum, buffy coat, and cryopreserved whole blood (1 collection), were collected from participants assigned to the screening arm, and buccal cells were collected from participants assigned to the community care arm. Incident cancers are ascertained by annual mailed questionnaires, other self-reports, and National Death Index (NDI) review. Confirmation of diagnosis and pathology reports is sought for all cancers; mortality is obtained through PLCO Trial sources and NDI reviews.

**ACS CPS-II Cohort:** The American Cancer Society Cancer Prevention Study II Nutrition Cohort (CPS-II) was established in 1992; the cohort includes over 86,000 men and 97,000 women who completed a mailed questionnaire in 1992. Starting in 1997, follow-up questionnaires have been sent to surviving cohort members every other year to update exposure information and to ascertain occurrence of new cases of cancer; a >90% response rate has been achieved for each follow-up questionnaire. Incident cancers are verified through medical records, state cancer registries, or death certificates. From 1998 - 2001, blood samples were collected from a subgroup of 39,376 cohort members. To further supplement the DNA resources, during 2000 - 2001, buccal cell samples were collected by mail from an additional 70,000 cohort members. For all cases, exposure information was collected by questionnaire before the cancer diagnosis.
