# Supplementary material for: Genome-wide association study of endometrial cancer in E2C2
Source: Hum Genet. 2013 Oct 6;133(2):211–24. doi: 10.1007/s00439-013-1369-1 (PMC3898362; doi:10.1007/s00439-013-1369-1)
Supplement: Supplementary file 2 — Supplementary material 2 (PDF 214 kb) [file 439_2013_1369_MOESM2_ESM.pdf]

Supplementary Table 2a: Fast track replication in participants of European non-Hispanic ancestry (N = 4270)

			GWAS					Replication[b]					GWAS + Replication				
SNP	CHR	A	Beta	StdErr	Q	P-Assoc	P-Het	Beta	StdErr	Q	P-Assoc	P-Het	Beta	StdErr	Q	P-Assoc	P-Het
RS10739046	9p24.2	C	-0.1949	0.0416	2.10	2.8E-06	0.91	-0.0120	0.0475	3.48	0.80	0.48	-0.1155	0.0313	11.88	2.2E-04	0.04
RS10822320	10q21.3	T	-0.2849	0.0641	5.67	8.9E-06	0.34	0.0964	0.0718	1.82	0.18	0.61	-0.1154	0.0478	17.54	1.6E-02	0.004
RS11651755 (HNF1B)	17q12	C	-0.0684	0.0435	5.98	1.2E-01	0.31	-0.1503	0.0442	3.61	7.0E-04	0.46	-0.1083	0.0310	5.33	4.8E-04	0.38
RS1352075	11q13.3	C	-0.2011	0.0442	2.29	5.3E-06	0.81	-0.0671	0.0516	3.41	0.19	0.33	-0.1445	0.0336	7.30	1.7E-05	0.20
RS4430796 (HNF1B)	17q12	G	-0.0845	0.0399	9.02	3.4E-02	0.17	-0.1253	0.0440	3.86	4.4E-03	0.42	-0.1027	0.0296	4.33	5.2E-04	0.50
RS4697273	4p15.2	A	-0.2487	0.0534	5.47	3.2E-06	0.36	0.0514	0.0539	1.06	0.34	0.90	-0.1001	0.0379	16.71	8.3E-03	0.005
RS9344	11q13.3	A	0.1840	0.0397	2.66	3.6E-06	0.85	0.0542	0.0514	2.20	0.29	0.53	0.1354	0.0314	6.19	1.6E-05	0.29
RS9369262[a]	6p24.1	C	-0.4497	0.0986	NA	5.1E-06	NA	-0.0466	0.0468	0.99	0.32	0.91	-0.1207	0.0423	14.64	4.3E-03	0.01
RS941990	6p22.3	G	0.2090	0.0469	3.69	8.3E-06	0.72	0.0124	0.0596	5.82	0.84	0.12	0.1346	0.0369	12.47	2.7E-04	0.03

[a]Genotyped only in PECS for the GWAS

[b]Contributing studies: AHS, EDGE, FHCRC, MEC, CPSII, Turin, Wise

Supplementary Table 2b. Fast track replication in participants of Black, Latina, Asian, and Hawaiian ancestry (N = 1284)

			BLACK (N=455)[d]					LATINA (N=382)[e]					ASIAN (N=366)[f]			HAWAIIAN (N=81)[f]		
SNP	CHR	A	Beta	StdErr	Q	P-Assoc	P-Het	Beta	StdErr	Q	P-Assoc	P-Het	Beta	StdErr	P-Assoc	Beta	StdErr	P-Assoc
RS10739046	9p24.2	C	0.1369	0.1711	2.22	0.42	0.53	-0.0159	0.1585	1.47	0.92	0.23	-0.1649	0.1578	0.30	-0.0680	0.3446	0.84
RS10822320*	10q21.3	T	0.1961	0.1886	4.85	0.30	0.09	0.0889	0.1566	1.74	0.57	0.42	-0.1040	0.1535	0.50	-1.1379	0.5736	0.05
RS11651755 (HNF1B)	17q12	C	-0.0905	0.1559	0.67	0.56	0.88	-0.1329	0.1663	2.71	0.42	0.26	-0.3597	0.1744	0.04	0.0961	0.3535	0.79
RS1352075	11q13.3	C	0.2364	0.1736	3.00	0.17	0.39	0.0156	0.1565	0.23	0.92	0.89	-0.1267	0.2114	0.55	0.6335	0.4029	0.12
RS4430796 (HNF1B)	17q12	G	-0.1159	0.1544	1.05	0.45	0.79	-0.1016	0.1673	1.68	0.54	0.43	-0.3646	0.1672	0.03	0.0319	0.3736	0.93
RS4697273	4p15.2	A	-0.5752	0.3026	0.22	0.06	0.64	-0.2276	0.1781	2.01	0.20	0.37	0.4878	0.1891	0.01	-0.2629	0.4235	0.53
RS9344	11q13.3	A	-0.1436	0.1791	0.68	0.42	0.88	0.0585	0.1570	0.22	0.71	0.90	-0.0727	0.1544	0.64	-0.0506	0.3379	0.88
RS9369262	6p24.1	C	-0.1524	0.1802	3.46	0.40	0.33	-0.3774	0.1570	1.24	0.02	0.54	-0.1558	0.1710	0.36	-0.3895	0.3629	0.28
RS941990[c]	6p22.3	G	-0.3225	0.1952	0.01	0.10	0.93	-0.0342	0.1550	0.92	0.83	0.63	0.2970	0.1874	0.11	0.1016	0.3149	0.75

[c]Excludes WISE Black participants (N=193) due to failed genotyping

[d]Black subjects were from EDGE, FHCRC, MEC and WISE

[e]Latinas from EDGE, FHCRC, and MEC

[f]Asians and Hawaiians from MEC only (Q and P-Het not applicable)
