# Supplementary material for: Genome-wide association study of endometrial cancer in E2C2
Source: Hum Genet. 2013 Oct 6;133(2):211–24. doi: 10.1007/s00439-013-1369-1 (PMC3898362; doi:10.1007/s00439-013-1369-1)
Supplement: Supplementary file 4 — Supplementary material 4 (DOCX 216 kb) [file 439_2013_1369_MOESM4_ESM.docx]

|  | | | | | |
| --- | --- | --- | --- | --- | --- |
| LOCUS | CHR | LOCATION | GENE | P_WHITES^a^ | P_ALL^b^ |
| rs4407 | 22 | 48741798 |  | 2.72E-06 | 2.29E-06 |
| rs12778749 | 10 | 127846669 | ADAM12 | 7.67E-06 | 1.01E-05 |
| rs3773582 | 3 | 55017749 | CACNA2D3 | 7.70E-06 | 0.0001782 |
| rs9459805 | 6 | 167336151 | RNASET2,LOC100131869 | 7.82E-06 | 2.10E-05 |
| rs1777220 | 6 | 126022602 | LOC643623 | 1.30E-05 | 8.55E-05 |
| rs4730998 | 7 | 120819121 | C7orf58 | 1.36E-05 | 0.0001792 |
| rs11679180 | 2 | 229642422 |  | 1.44E-05 | 2.37E-05 |
| rs686181 | X | 84198075 | RPS6KA6,UBE2DNL | 2.14E-05 | 1.56E-05 |
| rs7050236 | X | 67173332 |  | 2.81E-05 | 8.71E-05 |
| rs7034893 | 9 | 5494438 | PDCD1LG2 | 3.23E-05 | 0.00152 |
| rs17143147 | 7 | 120719961 | C7orf58 | 4.28E-05 | 3.61E-05 |
| rs6893754 | 5 | 56100006 | MAP3K1 | 4.35E-05 | 0.001099 |
| rs7178187 | 15 | 99683251 | SYNM,TTC23 | 4.71E-05 | 5.55E-05 |
| rs1453082 | 18 | 55936104 | NEDD4L | 6.61E-05 | 8.91E-05 |
| rs12521272 | 5 | 35190327 | PRLR | 6.82E-05 | 5.50E-05 |
| rs2981578 | 10 | 123340311 | FGFR2,LOC100131885 | 7.39E-05 | 7.42E-05 |
| rs2306692 | 12 | 57541265 | LRP1 | 7.66E-05 | 8.95E-05 |
| rs1649949 | 10 | 83916424 | NRG3 | 7.85E-05 | 0.000375 |
| rs1692108 | 12 | 55095662 |  | 8.34E-05 | 9.53E-05 |
| rs11073126 | 15 | 36116795 |  | 8.47E-05 | 0.0001304 |
| rs10822320 | 10 | 66364116 |  | 9.30E-05 | 0.0004459 |
| rs13396253 | 2 | 105027124 | LOC402093 | 9.62E-05 | 0.01315 |
| rs4407359 | 3 | 65597953 | MAGI1 | 0.0001009 | 0.001672 |
| rs1995971 | 7 | 79930551 |  | 0.0001025 | 0.0001639 |
| rs4816153 | 20 | 1012798 |  | 0.0001178 | 0.0002023 |
| rs9901473 | 17 | 66794431 |  | 0.0001188 | 0.0005111 |
| rs1351965 | 3 | 142123384 | XRN1 | 0.0001198 | 0.0001948 |
| rs13135778 | 4 | 123664446 | LOC729338,BBS12,LOC727709 | 0.0001225 | 0.0001539 |
| rs4925 | 10 | 106022789 | GSTO1,GSTO2 | 0.0001261 | 0.0002155 |
| rs2891316 | 9 | 28587994 | LINGO2 | 0.0001289 | 0.0003323 |
| rs7192139 | 16 | 5762949 |  | 0.00013 | 0.0005771 |
| rs6880595 | 5 | 35161332 | PRLR | 0.0001381 | 0.0002678 |
| rs1574433 | 11 | 117347947 | DSCAML1 | 0.0001432 | 8.15E-05 |
| rs10466868 | 12 | 131939920 |  | 0.0001481 | 0.0002276 |
| rs9867514 | 3 | 76689925 |  | 0.0001494 | 0.0005371 |
| rs7313455 | 12 | 56853231 | TIMELESS,MIP,SPRYD4 | 0.0001844 | 0.0005085 |
| rs2884127 | 10 | 17578161 |  | 0.0001994 | 7.57E-05 |
| rs602445 | 11 | 117958148 | TMPRSS4 | 0.0002035 | 0.0005707 |
| rs13185299 | 5 | 141525620 | NDFIP1 | 0.0002054 | 0.0001105 |
| rs5940807 | X | 89552827 | LOC100131981 | 0.0002283 | 0.0005448 |
| rs7182113 | 15 | 78392357 | SH2D7,CIB2 | 0.0002476 | 0.002754 |
| rs13138426 | 4 | 123652580 | LOC729338,BBS12,LOC727709 | 0.0002596 | 0.000339 |
| rs7007961 | 8 | 67276508 |  | 0.0002799 | 0.001265 |
| rs9313736 | 5 | 175948316 | RNF44 | 0.0003175 | 0.0001671 |
| rs11024021 | 11 | 16735491 |  | 0.0003282 | 0.001981 |
| rs6044832 | 20 | 17433598 | PCSK2 | 0.0003407 | 0.0009364 |
| rs11577262 | 1 | 30918166 |  | 0.000401 | 7.90E-05 |
| rs6724138 | 2 | 55314518 |  | 0.0004045 | 0.001162 |
| rs9698813 | X | 143607592 |  | 0.0004277 | 0.0002398 |
| rs12514742 | 5 | 35188476 | PRLR | 0.0004344 | 0.0002632 |
| rs882486 | 10 | 112294424 |  | 0.0004807 | 0.00863 |
| rs1371608 | 7 | 12406826 | VWDE | 0.0004813 | 0.0006558 |
| rs11644898 | 16 | 80754757 | CDYL2 | 0.0004922 | 0.0009343 |
| rs655089 | 11 | 69448575 | CCND1 | 0.0004956 | 0.001553 |
| rs13160147 | 5 | 36191407 | SKP2,C5orf33 | 0.0005157 | 0.001435 |
| rs9884230 | 4 | 169641425 | PALLD | 0.0005258 | 0.002123 |
| rs11082688 | 18 | 46168576 | KIAA0427 | 0.0005326 | 0.001329 |
| rs2546532 | 5 | 143595109 | KCTD16 | 0.0005525 | 0.001715 |
| rs308447 | 4 | 123738546 | FGF2 | 0.0005737 | 0.0004004 |
| rs9842553 | 3 | 59306653 |  | 0.0005798 | 0.0002507 |
| rs243196 | 14 | 90043381 | FOXN3,PRO1768 | 0.0006108 | 0.004656 |
| rs879013 | 20 | 1009679 |  | 0.0006127 | 0.0003037 |
| rs17860116 | 21 | 34602321 | IFNAR2 | 0.0006264 | 0.009864 |
| rs7793669 | 7 | 105639848 | FLJ23834 | 0.0006336 | 0.0006145 |
| rs754672 | 11 | 113249136 | ANKK1 | 0.0006497 | 0.0005719 |
| rs1998521 | 10 | 5992119 | IL15RA | 0.0006569 | 0.004649 |
| rs17599340 | 15 | 51039891 | SPPL2A | 0.0006572 | 0.0004669 |
| rs8125955 | 20 | 3019957 | PTPRA,GNRH2,MRPS26 | 0.0006655 | 0.001451 |
| rs992089 | 3 | 158684240 |  | 0.0007058 | 0.0007835 |
| rs9487737 | 6 | 112175517 | FYN | 0.0007262 | 0.004156 |
| rs11797015 | X | 125414651 |  | 0.0007322 | 0.0005174 |
| rs17252027 | 13 | 51751705 | RPL5P31 | 0.0007485 | 0.0006838 |
| rs7668932 | 4 | 160149875 |  | 0.000752 | 0.002291 |
| rs32128 | 5 | 11343384 | CTNND2 | 0.0007652 | 0.001473 |
| rs719802 | 11 | 113234679 | TTC12 | 0.000772 | 0.001797 |
| rs3738541 | 1 | 236749626 | HEATR1 | 0.0007744 | 0.001831 |
| rs7846809 | 9 | 5748232 | KIAA1432 | 0.0007791 | 0.003941 |
| rs17072860 | 4 | 183136858 |  | 0.0008086 | 0.001712 |
| rs17120398 | 14 | 85355544 |  | 0.0008094 | 0.002222 |
| rs11082877 | 18 | 49014615 |  | 0.0008112 | 0.002457 |
| rs1499242 | 11 | 22793809 | GAS2 | 0.0008123 | 0.0282 |
| rs10488790 | 11 | 29478337 | LOC729560 | 0.0008236 | 7.46E-05 |
| rs10887831 | 10 | 90268918 | RNLS | 0.0008266 | 0.0004768 |
| rs4820242 | 22 | 36982675 | CACNG2 | 0.0008587 | 0.001 |
| rs12528660 | 6 | 19876533 |  | 0.0008695 | 0.00186 |
| rs7791858 | 7 | 92136754 | PEX1 | 0.0009039 | 0.009652 |
| rs7684974 | 4 | 165944299 | TRIM60 | 0.0009414 | 0.00278 |
| rs17487218 | 4 | 165837436 | LOC653794,RPL26P16 | 0.0009649 | 0.0002082 |
| rs7769272 | 6 | 125899476 |  | 0.0009712 | 0.00325 |
| rs11727514 | 4 | 175682850 | GLRA3 | 0.0009906 | 0.003528 |
| rs17513440 | 4 | 21175390 | KCNIP4 | 0.001015 | 0.002727 |
| rs9458764 | 6 | 163657117 | PACRG | 0.00102 | 0.001081 |
| rs591372 | 11 | 74146105 |  | 0.001059 | 0.0067 |
| rs2136150 | 3 | 7353942 | GRM7 | 0.001073 | 0.0025 |
| rs12289084 | 11 | 134020617 | JAM3,NCAPD3 | 0.00108 | 0.002976 |
| rs4530225 | 18 | 38630544 |  | 0.001082 | 0.002283 |
| rs16840284 | 1 | 240680957 | GREM2 | 0.001099 | 0.00394 |
| rs9930333 | 16 | 53799977 | FTO | 0.001102 | 0.001251 |
| rs3803220 | 13 | 25241628 | ATP12A | 0.001107 | 0.001451 |
| rs4240889 | 1 | 152534954 | LCE3E | 0.001165 | 0.0006868 |
| rs4812563 | 20 | 40579609 |  | 0.001218 | 0.0001805 |
| rs10489526 | 1 | 115293527 | CSDE1 | 0.001235 | 0.001027 |
| rs17781793 | 12 | 67739929 | LOC645328 | 0.001237 | 0.0009405 |
| rs12714456 | 3 | 77104596 | ROBO2 | 0.00126 | 0.01298 |
| rs4485736 | 3 | 141925039 | GK5 | 0.001263 | 0.001514 |
| rs9471976 | 6 | 42920549 | RPL24P4,GNMT | 0.00137 | 0.003638 |
| rs17243220 | 6 | 24662764 | KIAA0319,TTRAP,ACOT13 | 0.001375 | 0.002037 |
| rs17441991 | 10 | 45736643 | OR6D1P | 0.001408 | 0.0008999 |
| rs12212378 | 6 | 150589471 |  | 0.001415 | 0.003531 |
| rs6637581 | X | 128495691 |  | 0.001475 | 0.002826 |
| rs832404 | 5 | 56267431 | MIER3,LOC100130001 | 0.001538 | 0.0006496 |
| rs6760802 | 2 | 11398736 | ROCK2 | 0.001576 | 0.001419 |
| rs6827817 | 4 | 88330987 | HSD17B11,LOC100129693,NUDT9 | 0.001594 | 0.002584 |
| rs17080959 | 6 | 151575944 | AKAP12 | 0.001604 | 0.001315 |
| rs2048343 | 5 | 84242662 |  | 0.001624 | 0.001226 |
| rs4130504 | 4 | 163955092 |  | 0.00164 | 0.0007696 |
| rs1274762 | 14 | 69155627 | RPL12P7 | 0.001643 | 0.0007734 |
| rs7131430 | 11 | 7597280 | PPFIBP2 | 0.001653 | 0.002328 |
| rs1549661 | 16 | 76367157 | CNTNAP4 | 0.001658 | 0.003228 |
| rs13007757 | 2 | 143610406 | LOC100129955 | 0.001694 | 0.002501 |
| rs1011204 | 6 | 2687541 | MYLK4 | 0.001756 | 0.003783 |
| rs7819010 | 8 | 118368791 |  | 0.001806 | 0.001092 |
| rs4904358 | 14 | 87501042 |  | 0.001904 | 0.0128 |
| rs1789170 | 11 | 69509343 | ORAOV1,FGF19 | 0.001921 | 0.003744 |
| rs7626433 | 3 | 133543917 | SRPRB,RAB6B | 0.001934 | 0.00311 |
| rs2579085 | 12 | 90581532 |  | 0.002004 | 0.001435 |
| rs651979 | 11 | 103859645 | PDGFD | 0.002037 | 0.005131 |
| rs9598051 | 13 | 34318946 |  | 0.00207 | 0.0002692 |
| rs6943781 | 7 | 41871611 |  | 0.002101 | 0.003877 |
| rs6912709 | 6 | 67641300 |  | 0.002187 | 0.0007057 |
| rs6774637 | 3 | 141952543 | GK5 | 0.002255 | 0.0008488 |
| rs9838009 | 3 | 59265315 |  | 0.002302 | 0.001819 |
| rs668204 | 11 | 113149642 | NCAM1 | 0.002313 | 0.003116 |
| rs1431372 | 21 | 29184528 |  | 0.002374 | 0.01703 |
| rs16973694 | 18 | 38290277 |  | 0.002417 | 0.006891 |
| rs1028861 | 1 | 222426667 |  | 0.002428 | 0.001057 |
| rs9956244 | 18 | 48195558 | MAPK4 | 0.002476 | 0.0009938 |
| rs12636203 | 3 | 75186326 |  | 0.002516 | 0.0004541 |
| rs7760703 | 6 | 39859667 | DAAM2,LOC100132140 | 0.002521 | 0.008176 |
| rs314078 | 4 | 189296076 |  | 0.002553 | 0.004025 |
| rs2834940 | 21 | 36836921 | RPL34P3 | 0.002564 | 0.00245 |
| rs10956429 | 8 | 129514987 |  | 0.002618 | 0.01329 |
| rs4951309 | 1 | 204112406 | ETNK2 | 0.00262 | 0.006545 |
| rs6906626 | 6 | 51676922 | PKHD1 | 0.002632 | 0.0115 |
| rs2906964 | 17 | 15331958 | CDRT4,RPL9P2 | 0.002652 | 0.003648 |
| rs3746427 | 20 | 33730464 | EDEM2 | 0.002682 | 0.002661 |
| rs10738389 | 9 | 15179840 | TTC39B | 0.002688 | 0.001207 |
| rs596866 | 10 | 6550199 | PRKCQ | 0.002696 | 0.001078 |
| rs13320391 | 3 | 155055548 |  | 0.00277 | 0.001619 |
| rs7943620 | 11 | 29275928 |  | 0.002798 | 0.00174 |
| rs11021984 | 11 | 2282360 | ASCL2 | 0.002827 | 0.005723 |
| rs12437455 | 15 | 68878577 | LOC100129840,CORO2B | 0.002906 | 0.002067 |
| rs16955797 | 15 | 31322565 | TRPM1 | 0.002925 | 0.002147 |
| rs6747687 | 2 | 3123061 |  | 0.002942 | 0.01115 |
| rs6468613 | 8 | 88009165 | CNBD1 | 0.002944 | 0.0006187 |
| rs215903 | 16 | 21729068 | OTOA | 0.002957 | 0.002075 |
| rs13025842 | 2 | 68546449 | CNRIP1 | 0.003039 | 0.01335 |
| rs1386443 | 8 | 122358307 |  | 0.003069 | 0.005423 |
| rs1445998 | 5 | 56268205 | LOC100130001 | 0.003123 | 0.01361 |
| rs4703509 | 5 | 35200194 | PRLR | 0.003195 | 0.003056 |
| rs1428925 | 5 | 83329010 | EDIL3 | 0.0032 | 0.07399 |
| rs2047370 | 3 | 87211142 |  | 0.003241 | 0.02501 |
| rs10498722 | 6 | 25186512 |  | 0.003304 | 0.002729 |
| rs12199027 | 6 | 130418172 | L3MBTL3 | 0.00339 | 0.003338 |
| rs697864 | 12 | 116254489 |  | 0.003464 | 0.001108 |
| rs4652352 | 1 | 179042300 | FAM20B,TOR3A | 0.003536 | 0.002124 |
| rs7225247 | 17 | 52933176 |  | 0.003752 | 0.01055 |
| rs855402 | 6 | 10044051 | OFCC1 | 0.003772 | 0.001936 |
| rs16883397 | 5 | 9698629 |  | 0.003792 | 0.00848 |
| rs4715996 | 6 | 15758316 |  | 0.003847 | 0.01949 |
| rs749932 | 3 | 41378621 | ULK4 | 0.003856 | 0.001057 |
| rs13287975 | 9 | 105399642 | LOC100131879 | 0.003863 | 0.005077 |
| rs16880561 | 4 | 28378913 |  | 0.003892 | 0.007649 |
| rs2131464 | 4 | 28795758 |  | 0.003982 | 0.03275 |
| rs10739046 | 9 | 4316336 | GLIS3 | 0.004028 | 0.002114 |
| rs6130333 | 20 | 41921716 |  | 0.004035 | 0.008618 |
| rs12536378 | 7 | 154058474 | DPP6 | 0.004036 | 0.002191 |
| rs1530394 | 2 | 31363732 | GALNT14 | 0.004089 | 0.006 |
| rs4820239 | 22 | 36958908 | CACNG2 | 0.004098 | 0.004282 |
| rs2338967 | 17 | 32923810 | C17orf102,TMEM132E | 0.004136 | 0.0135 |
| rs1041321 | 9 | 32394422 | ACO1 | 0.004158 | 0.009893 |
| rs9766943 | 6 | 124763669 | NKAIN2 | 0.004204 | 0.01658 |
| rs2293373 | 3 | 133547692 | SRPRB,RAB6B | 0.004246 | 0.006978 |
| rs7311709 | 12 | 59636969 |  | 0.004328 | 0.002429 |
| rs16830373 | 2 | 195753257 |  | 0.004393 | 0.004583 |
| rs2294689 | 6 | 24653273 | KIAA0319,TTRAP,ACOT13 | 0.004439 | 0.0256 |
| rs678570 | 18 | 6975451 | LAMA1 | 0.004462 | 0.002161 |
| rs4140566 | 6 | 97397823 | KLHL32 | 0.004514 | 0.01289 |
| rs1454242 | 2 | 3848532 |  | 0.00454 | 0.009053 |
| rs11621961 | 14 | 94769476 | SERPINA10,SERPINA6 | 0.004542 | 0.00529 |
| rs11066633 | 12 | 114044030 |  | 0.004567 | 0.003641 |
| rs11187348 | 10 | 95016235 |  | 0.004634 | 0.0017 |
| rs1862626 | 5 | 56032940 |  | 0.004641 | 0.005974 |
| rs2204039 | 3 | 36694732 | NBPF21P | 0.004644 | 0.01177 |
| rs10936537 | 3 | 167132319 |  | 0.004672 | 0.00591 |
| rs2086720 | 2 | 48215197 | VN1R18P | 0.004698 | 0.00632 |
| rs4318522 | 3 | 146594746 |  | 0.004746 | 0.002252 |
| rs1405376 | 4 | 72515164 |  | 0.004748 | 0.003241 |
| rs604449 | 8 | 116005882 |  | 0.00481 | 0.004879 |
| rs7103667 | 11 | 76747390 | B3GNT6 | 0.004899 | 0.001644 |
| rs10823571 | 10 | 72316867 | KIAA1274 | 0.00491 | 0.0214 |
| rs7429544 | 3 | 75285488 |  | 0.004938 | 0.01917 |
| rs1870259 | 3 | 28390351 | AZI2 | 0.004955 | 0.003096 |
| rs7970739 | 12 | 76232239 |  | 0.004967 | 0.003604 |
| rs9403037 | 6 | 139418007 |  | 0.004971 | 0.004134 |
| rs11800014 | 1 | 22414070 | CDC42,LOC729796 | 0.005025 | 0.01421 |
| rs315721 | 5 | 169715038 | LCP2 | 0.005106 | 0.03678 |
| rs11786582 | 8 | 119385578 | SAMD12 | 0.005133 | 0.01713 |
| rs1551050 | 5 | 16533551 | FAM134B | 0.005207 | 0.009982 |
| rs11668097 | 19 | 53465008 | ZNF321,ZNF816A,ZNF702P | 0.005258 | 0.008075 |
| rs6089866 | 20 | 61872309 | BIRC7,NKAIN4 | 0.00531 | 0.01382 |
| rs13082346 | 3 | 40929572 |  | 0.005347 | 0.001867 |
| rs4697273 | 4 | 22235796 |  | 0.005481 | 0.009118 |
| rs17019462 | 2 | 4628979 |  | 0.005512 | 0.002846 |
| rs9811119 | 3 | 167160385 | SERPINI2 | 0.005518 | 0.007165 |
| rs1739766 | 10 | 83875762 | NRG3 | 0.005521 | 0.003315 |
| rs12611955 | 2 | 175591073 | LOC100133109,LOC440926 | 0.005525 | 0.003593 |
| rs1465641 | 2 | 106438936 | NCK2 | 0.005525 | 0.006086 |
| rs2645950 | 3 | 130224642 |  | 0.005548 | 0.0251 |
| rs1404057 | 2 | 48924091 | GTF2A1L,LHCGR | 0.005562 | 0.009955 |
| rs666156 | 10 | 105911963 | C10orf79,LOC751602 | 0.005702 | 0.007581 |
| rs17512637 | 4 | 40420804 | RBM47 | 0.005726 | 0.002672 |
| rs16970049 | 15 | 40251595 | EIF2AK4,H3F3AP1 | 0.00577 | 0.08258 |
| rs1547727 | X | 145210623 |  | 0.005781 | 0.007424 |
| rs2252403 | 17 | 38934285 | KRT26,KRT27 | 0.005792 | 0.007449 |
| rs11699653 | 20 | 34622916 | C20orf152 | 0.005802 | 0.01384 |
| rs17070739 | 18 | 60819383 | BCL2 | 0.005878 | 0.0925 |
| rs12345835 | 9 | 18916311 | ADAMTSL1 | 0.005932 | 0.01063 |
| rs11195144 | 10 | 112233212 |  | 0.005944 | 0.01143 |
| rs2072963 | 20 | 1099294 | PSMF1 | 0.006089 | 0.02108 |
| rs7507285 | 19 | 30732502 |  | 0.006139 | 0.006486 |
| rs9487747 | 6 | 112231090 |  | 0.006193 | 0.005873 |
| rs4384310 | 10 | 133253673 |  | 0.006213 | 0.07552 |
| rs2827079 | 21 | 23196432 |  | 0.006226 | 0.02488 |
| rs9687397 | 5 | 34575747 |  | 0.006328 | 0.006617 |
| rs869802 | 13 | 102529512 | FGF14 | 0.006338 | 0.007283 |
| rs3091869 | 20 | 45425918 |  | 0.006351 | 0.01857 |
| rs351676 | 2 | 143711265 | KYNU,LOC100131793 | 0.006434 | 0.01938 |
| rs4646887 | 1 | 165654812 | ALDH9A1 | 0.006466 | 0.009246 |
| rs1884499 | 20 | 55959429 | RAE1,RBM38 | 0.006468 | 0.04159 |
| rs17366047 | 9 | 121137235 |  | 0.00648 | 0.003245 |
| rs4144737 | 8 | 130761182 | GSDMC | 0.006562 | 0.004113 |
| rs9360252 | 6 | 67656128 |  | 0.006594 | 0.002689 |
| rs526108 | 11 | 95154173 |  | 0.006714 | 0.006373 |
| rs12640505 | 4 | 40262814 |  | 0.006717 | 0.006551 |
| rs10247842 | 7 | 8522345 | NXPH1 | 0.00674 | 0.01809 |
| rs2235587 | 20 | 1115919 | PSMF1 | 0.006748 | 0.006118 |
| rs1289305 | 4 | 104353539 |  | 0.006792 | 0.01109 |
| rs10133677 | 14 | 47746980 | MDGA2 | 0.006801 | 0.008728 |
| rs2060983 | 8 | 131025034 |  | 0.006945 | 0.01599 |
| rs3791765 | 2 | 10099635 | GRHL1 | 0.006986 | 0.003045 |
| rs4571555 | 6 | 22155622 | LOC729177 | 0.007031 | 0.005572 |
| rs4484960 | 1 | 45765523 | hCG_1820661 | 0.007145 | 0.006735 |
| rs12777369 | 10 | 59808144 |  | 0.007189 | 0.01916 |
| rs11177408 | 12 | 69289749 | CPM | 0.00723 | 0.01008 |
| rs9294147 | 6 | 80191532 | LCA5 | 0.007295 | 0.00202 |
| rs4410962 | 9 | 105207670 |  | 0.007306 | 0.007419 |
| rs17036281 | 3 | 12354411 | PPARG | 0.007332 | 0.01608 |
| rs17831280 | 15 | 88742512 | NTRK3 | 0.007385 | 0.02323 |
| rs13103414 | 4 | 139833891 | LOC100128578 | 0.007425 | 0.02925 |
| rs10975148 | 9 | 5500300 | PDCD1LG2 | 0.007481 | 0.03838 |
| rs1985053 | 21 | 21791915 | KRT18P2,LOC391271,RPS3AP1 | 0.007573 | 0.007379 |
| rs4653395 | 1 | 34615576 | CSMD2 | 0.007739 | 0.02945 |
| rs2300769 | 3 | 179420666 | USP13 | 0.007759 | 0.01155 |
| rs6585753 | 10 | 123443881 |  | 0.007778 | 0.01769 |
| rs17078780 | 8 | 2748821 |  | 0.00778 | 0.004907 |
| rs6546646 | 2 | 71101403 |  | 0.007805 | 0.04055 |
| rs10985459 | 9 | 124703332 |  | 0.00785 | 0.005732 |
| rs6532059 | 4 | 89163923 |  | 0.008019 | 0.01561 |
| rs12528572 | 6 | 108658974 | LACE1 | 0.008069 | 0.009128 |
| rs10194837 | 2 | 228243310 | TM4SF20 | 0.008109 | 0.004179 |
| rs931804 | 5 | 172034100 |  | 0.008122 | 0.005616 |
| rs6632085 | X | 34823049 |  | 0.008134 | 0.007623 |
| rs852069 | 20 | 17122593 |  | 0.008153 | 0.003586 |
| rs2036831 | 15 | 45263499 | C15orf43 | 0.008215 | 0.02476 |
| rs13074881 | 3 | 164217297 |  | 0.008231 | 0.009364 |
| rs4656457 | 1 | 165652568 | ALDH9A1 | 0.008239 | 0.01299 |
| rs8048537 | 16 | 85230723 |  | 0.008285 | 0.03638 |
| rs2560851 | 5 | 16887383 | MYO10 | 0.008318 | 0.005101 |
| rs4646889 | 1 | 165654291 | ALDH9A1 | 0.008324 | 0.01211 |
| rs7100031 | 10 | 124818438 | ACADSB | 0.008362 | 0.02032 |
| rs10753688 | 1 | 165666448 | ALDH9A1,LOC440700 | 0.00837 | 0.0122 |
| rs4407726 | 6 | 150611573 |  | 0.008381 | 0.0136 |
| rs250147 | 16 | 79591972 |  | 0.008441 | 0.02745 |
| rs10782790 | 1 | 83087776 |  | 0.008556 | 0.0159 |
| rs3764066 | 13 | 23744015 | SGCG | 0.008612 | 0.01321 |
| rs17148768 | 10 | 10777085 |  | 0.00865 | 0.004273 |
| rs2247788 | 21 | 43067245 |  | 0.008761 | 0.02253 |
| rs158639 | 5 | 55611710 |  | 0.00878 | 0.0775 |
| rs2102911 | 1 | 242148832 | CFLP4,MAP1LC3C | 0.008796 | 0.0223 |
| rs17757896 | 18 | 38455134 |  | 0.008854 | 0.008966 |
| rs9616084 | 22 | 46480602 | LOC642648,LOC400931 | 0.008872 | 0.01165 |
| rs6617745 | X | 88004302 | CPXCR1 | 0.00891 | 0.01079 |
| rs11816423 | 10 | 91881662 |  | 0.008986 | 0.02096 |
| rs2064454 | 20 | 33496169 | ACSS2 | 0.00904 | 0.01436 |
| rs3744021 | 17 | 73871911 | TRIM47 | 0.009043 | 0.01753 |
| rs16863956 | 2 | 5708764 |  | 0.009177 | 0.02307 |
| rs708827 | 12 | 116253777 |  | 0.009346 | 0.003399 |
| rs17137306 | 7 | 114461205 |  | 0.009516 | 0.01802 |
| rs4918430 | 10 | 94899803 | LOC389997 | 0.009548 | 0.008872 |
| rs8045775 | 16 | 58867855 |  | 0.009629 | 0.04369 |
| rs6057825 | 20 | 31921539 |  | 0.009687 | 0.006719 |
| rs10510511 | 3 | 21260370 |  | 0.009714 | 0.05431 |
| rs30623 | 5 | 14332325 | TRIO,UNQ1870 | 0.009845 | 0.02444 |
| rs6562259 | 13 | 62733451 |  | 0.009879 | 0.01173 |
| rs3795828 | 1 | 110996325 | PROK1 | 0.00988 | 0.00418 |
| rs3763630 | 9 | 35731004 | TLN1,CREB3,GBA2,RGP1 | 0.009934 | 0.02409 |
| rs16926356 | 9 | 10727372 |  | 0.01 | 0.007068 |
| rs6949979 | 7 | 1792688 | ELFN1 | 0.01 | 0.008886 |
| rs1376080 | 18 | 44030044 | RNF165 | 0.01009 | 0.03837 |
| rs11097720 | 4 | 101388148 | EMCN | 0.01023 | 0.01307 |
| rs860580 | 5 | 56149429 | MAP3K1 | 0.01025 | 0.01411 |
| rs10486156 | 7 | 7348633 |  | 0.01028 | 0.02064 |
| rs10800956 | 1 | 190632794 |  | 0.0103 | 0.003367 |
| rs3136195 | 16 | 14036471 | ERCC4 | 0.01032 | 0.01329 |
| rs11764661 | 7 | 105632516 | FLJ23834 | 0.01049 | 0.01315 |
| rs4822857 | 22 | 27502567 |  | 0.01052 | 0.004416 |
| rs7572707 | 2 | 23176757 |  | 0.01057 | 0.008113 |
| rs1469322 | 8 | 53930798 |  | 0.01059 | 0.01276 |
| rs12099456 | 12 | 88658684 |  | 0.01062 | 0.005305 |
| rs3129591 | 13 | 22555886 |  | 0.01081 | 0.007649 |
| rs1282275 | 1 | 111629571 |  | 0.0109 | 0.01997 |
| rs4750886 | 10 | 129239267 | DOCK1 | 0.011 | 0.01536 |
| rs10911730 | 1 | 185406380 |  | 0.01105 | 0.01489 |
| rs9467251 | 6 | 24657168 | KIAA0319,TTRAP,ACOT13 | 0.01109 | 0.02086 |
| rs4416661 | 6 | 67688283 |  | 0.01118 | 0.03379 |
| rs219684 | 21 | 27733641 |  | 0.01121 | 0.005016 |
| rs9309641 | 2 | 88355735 | KRCC1,SMYD1 | 0.01122 | 0.02715 |
| rs1323194 | 13 | 102518690 | FGF14 | 0.01124 | 0.0135 |
| rs631752 | 15 | 42395260 | PLA2G4D | 0.01128 | 0.01875 |
| rs3816539 | 1 | 26786627 | DHDDS,RPL17P9,HMGN2 | 0.01132 | 0.01107 |
| rs12971263 | 18 | 38768209 |  | 0.01132 | 0.02805 |
| rs16852171 | 3 | 168044074 | LOC389174 | 0.01175 | 0.01185 |
| rs2828624 | 21 | 25299689 |  | 0.01184 | 0.0122 |
| rs1930465 | 10 | 59766998 |  | 0.01185 | 0.02117 |
| rs13094834 | 3 | 67467766 | SUCLG2 | 0.01199 | 0.01235 |
| rs6972422 | 7 | 18834375 | HDAC9 | 0.01211 | 0.006032 |
| rs1067147 | 14 | 48502282 |  | 0.01227 | 0.01033 |
| rs195371 | 6 | 37304386 | TBC1D22B,RNF8 | 0.01232 | 0.05938 |
| rs7803700 | 7 | 150464171 | GIMAP3P,tcag7.1033 | 0.01242 | 0.02523 |
| rs831751 | 1 | 201158976 | LOC440706 | 0.01243 | 0.01377 |
| rs7408 | 5 | 141380348 | GNPDA1 | 0.01247 | 0.01386 |
| rs32136 | 5 | 11336674 | CTNND2 | 0.01248 | 0.02475 |
| rs11120211 | 1 | 207276379 | C4BPB,C4BPA | 0.01251 | 0.02288 |
| rs491003 | 6 | 94861241 |  | 0.01252 | 0.006979 |
| rs902523 | 5 | 77216237 |  | 0.01255 | 0.05372 |
| rs9304531 | 18 | 26605972 |  | 0.01265 | 0.02739 |
| rs651529 | 1 | 244452911 |  | 0.01277 | 0.01165 |
| rs4388301 | 6 | 138282375 |  | 0.01281 | 0.03186 |
| rs17143575 | 7 | 20998875 |  | 0.01318 | 0.01058 |
| rs7001690 | 8 | 8600305 |  | 0.01319 | 0.02201 |
| rs453644 | 21 | 17743767 | C21orf34 | 0.0132 | 0.03607 |
| rs17068331 | 6 | 139416226 |  | 0.01327 | 0.009379 |
| rs10830204 | 11 | 88787544 | GRM5 | 0.01336 | 0.08234 |
| rs13395387 | 2 | 43076226 |  | 0.01338 | 0.01949 |
| rs6444106 | 3 | 185811495 | ETV5 | 0.01348 | 0.02961 |
| rs5006712 | 8 | 130759038 | GSDMC | 0.0136 | 0.01518 |
| rs10943605 | 6 | 79655477 | PHIP | 0.01363 | 0.01319 |
| rs929881 | 16 | 74808425 | FA2H | 0.01372 | 0.05203 |
| rs17777754 | 7 | 34790349 | NPSR1 | 0.0139 | 0.008981 |
| rs6820993 | 4 | 115897055 | NDST4 | 0.01397 | 0.02814 |
| rs10897823 | 11 | 81192641 |  | 0.01397 | 0.0428 |
| rs697212 | 12 | 104100617 | STAB2 | 0.01399 | 0.06141 |
| rs2590634 | 7 | 118534383 |  | 0.01403 | 0.04111 |
| rs9580437 | 13 | 23350509 |  | 0.01408 | 0.02746 |
| rs4923709 | 15 | 36618505 |  | 0.01412 | 0.004077 |
| rs6943492 | 7 | 142347046 | TRB@,TRBV21-1,TRBV22-1,TRBV23-1,TRBV24-1 | 0.0142 | 0.03014 |
| rs2837405 | 21 | 41429710 | DSCAM | 0.01423 | 0.004388 |
| rs16962997 | 15 | 50159269 | ATP8B4 | 0.01429 | 0.01543 |
| rs1568158 | 12 | 118060457 | KSR2 | 0.01436 | 0.01654 |
| rs717275 | 1 | 115610091 | TSPAN2 | 0.01437 | 0.02226 |
| rs10916851 | 1 | 21040151 | KIF17,SH2D5 | 0.01453 | 0.001256 |
| rs11673260 | 19 | 52181798 | MIR99B,hsa-mir-99b,MIRLET7E,hsa-let-7e,MIR125A,hsa-mir-125a,NCRNA00085 | 0.01456 | 0.01346 |
| rs10771185 | 12 | 9056410 | PHC1 | 0.01464 | 0.02342 |
| rs4076750 | 1 | 22423543 | CDC42 | 0.01466 | 0.02465 |
| rs6972380 | 7 | 121406624 |  | 0.0148 | 0.007233 |
| rs2094199 | 6 | 76793338 | IMPG1 | 0.01486 | 0.03963 |
| rs12461826 | 19 | 46731528 | RPL12P41,IGFL1 | 0.01489 | 0.03201 |
| rs13674 | 10 | 12877269 | CAMK1D | 0.015 | 0.02154 |
| rs603965 | 11 | 69462910 | CCND1,FLJ42258 | 0.01505 | 0.01058 |
| rs1423213 | 5 | 58178433 |  | 0.01512 | 0.008475 |
| rs3741906 | 12 | 6105143 | VWF | 0.01515 | 0.006315 |
| rs10913563 | 1 | 178483231 | NCRNA00083,C1orf49 | 0.01554 | 0.03051 |
| rs2300244 | 12 | 13840654 | GRIN2B | 0.01555 | 0.013 |
| rs542494 | 1 | 234424289 | SLC35F3 | 0.01561 | 0.01146 |
| rs9825287 | 3 | 38714855 |  | 0.01581 | 0.008221 |
| rs10073098 | 5 | 6156845 |  | 0.01581 | 0.01391 |
| rs4352622 | 5 | 119234983 |  | 0.01589 | 0.04204 |
| rs2720961 | 3 | 194769289 |  | 0.01593 | 0.01804 |
| rs4832159 | 2 | 85523541 | TCF7L1,LOC647305 | 0.01607 | 0.01798 |
| rs2139747 | 3 | 126387875 | TXNRD3,C3orf46,LOC645852 | 0.01608 | 0.03954 |
| rs4654986 | 1 | 22138198 | LDLRAD2 | 0.01613 | 0.02956 |
| rs690449 | 15 | 53499618 |  | 0.01616 | 0.003581 |
| rs12336476 | 9 | 89821983 | LOC100132348 | 0.01622 | 0.009182 |
| rs4743569 | 9 | 105414408 | LOC100131879 | 0.01632 | 0.01844 |
| rs9351022 | 6 | 85662325 |  | 0.01637 | 0.01922 |
| rs1399790 | 12 | 92917324 | LOC100132126 | 0.01649 | 0.07236 |
| rs10203913 | 2 | 65723237 | LOC100129140 | 0.01672 | 0.009696 |
| rs2390256 | 7 | 20177882 | MACC1 | 0.01679 | 0.01494 |
| rs11976076 | 7 | 26525891 | LOC100130672 | 0.01684 | 0.01795 |
| rs11733487 | 4 | 21236883 | KCNIP4 | 0.01697 | 0.01487 |
| rs12380966 | X | 153155987 | L1CAM,LCAP,AVPR2 | 0.01699 | 0.08011 |
| rs11652541 | 17 | 15651231 | TBC1D26,CDRT15L1 | 0.01708 | 0.0407 |
| rs2302768 | 18 | 12718593 | CEP76,PSMG2 | 0.01712 | 0.01414 |
| rs11675218 | 2 | 65215030 | SLC1A4 | 0.01712 | 0.01752 |
| rs12626495 | 21 | 26609246 |  | 0.01719 | 0.008742 |
| rs969527 | 6 | 9986985 | OFCC1 | 0.01719 | 0.03666 |
| rs12458935 | 18 | 36959010 |  | 0.01722 | 0.0298 |
| rs2317299 | 2 | 236903093 | AGAP1,LOC100131101 | 0.01729 | 0.01433 |
| rs16829304 | 1 | 189958783 | LOC647132 | 0.0174 | 0.005319 |
| rs10983950 | 9 | 121036075 |  | 0.01745 | 0.008253 |
| rs2544098 | 12 | 45977587 | LOC100131290 | 0.01754 | 0.0142 |
| rs216626 | 2 | 80814388 | CTNNA2 | 0.0176 | 0.02598 |
| rs9877751 | 3 | 75202372 |  | 0.01761 | 0.00557 |
| rs17631802 | 4 | 23595674 | LOC643751 | 0.01803 | 0.02618 |
| rs2967821 | 5 | 3846180 |  | 0.01813 | 0.01522 |
| rs7290713 | 22 | 33188545 | SYN3,TIMP3 | 0.01814 | 0.09369 |
| rs16856600 | 2 | 170058497 | LRP2 | 0.01827 | 0.02468 |
| rs2900119 | 5 | 107205967 | FBXL17 | 0.01846 | 0.02655 |
| rs1799017 | 7 | 78524614 | MAGI2 | 0.01857 | 0.01731 |
| rs2176489 | 10 | 15333431 | FAM171A1 | 0.01859 | 0.01559 |
| rs7659540 | 4 | 153008540 |  | 0.01862 | 0.03937 |
| rs9363656 | 6 | 67588454 |  | 0.01875 | 0.0055 |
| rs4934405 | 10 | 90188684 | RNLS | 0.01882 | 0.02734 |
| rs13411636 | 2 | 236532308 | AGAP1 | 0.01884 | 0.01439 |
| rs700750 | 7 | 46753491 | LOC730338 | 0.01894 | 0.004592 |
| rs2416944 | 12 | 12016045 | ETV6 | 0.01899 | 0.03477 |
| rs11678307 | 2 | 64644762 |  | 0.01906 | 0.01338 |
| rs2115138 | 5 | 119122508 |  | 0.0191 | 0.01529 |
| rs1341113 | 6 | 104792340 |  | 0.0192 | 0.0223 |
| rs1021795 | 3 | 26396178 | VENTXP4 | 0.01924 | 0.002676 |
| rs2835195 | 21 | 37347259 |  | 0.01932 | 0.04069 |
| rs6070131 | 20 | 56113557 | CTCFL | 0.01935 | 0.008351 |
| rs28374796 | 1 | 242149849 | CFLP4,MAP1LC3C | 0.01935 | 0.03814 |
| rs556146 | 11 | 100694891 | FLJ32810 | 0.01944 | 0.02592 |
| rs11968661 | 6 | 128014339 |  | 0.01976 | 0.02263 |
| rs8048816 | 16 | 75422201 | CFDP1 | 0.01984 | 0.02226 |
| rs11141494 | 9 | 89306305 |  | 0.01987 | 0.003226 |
| rs11101132 | 10 | 50637298 | LOC100128032 | 0.0199 | 0.008306 |
| rs4949652 | 1 | 77708940 |  | 0.02005 | 0.01903 |
| rs10980757 | 9 | 113853766 |  | 0.02011 | 0.02197 |
| rs10195033 | 2 | 62541900 |  | 0.02012 | 0.02011 |
| rs4243863 | 8 | 135745432 |  | 0.02012 | 0.05342 |
| rs3219492 | 1 | 45796354 | HPDL,MUTYH,TOE1 | 0.02015 | 0.01455 |
| rs28373959 | 1 | 242150334 | CFLP4,MAP1LC3C | 0.02017 | 0.04319 |
| rs321472 | 16 | 26745376 |  | 0.02019 | 0.0741 |
| rs258795 | 5 | 142540017 | ARHGAP26 | 0.0202 | 0.01171 |
| rs1474488 | 21 | 41370818 |  | 0.02032 | 0.01851 |
| rs4856202 | 3 | 81224298 |  | 0.02043 | 0.03314 |
| rs12468846 | 2 | 224693416 | AP1S3 | 0.02048 | 0.01105 |
| rs1561937 | 11 | 25585180 |  | 0.0207 | 0.0249 |
| rs399246 | 19 | 30595364 |  | 0.02083 | 0.01454 |
| rs193773 | 16 | 11298850 | LOC729954 | 0.02091 | 0.03916 |
| rs133845 | 22 | 26132839 | ADRBK2,MYO18B | 0.02093 | 0.0137 |
| rs7683416 | 4 | 106152984 | TET2 | 0.02096 | 0.01494 |
| rs17451107 | 3 | 156797609 |  | 0.02113 | 0.06543 |
| rs226384 | 12 | 9263647 | A2M,LOC100130074 | 0.02123 | 0.03465 |
| rs1133253 | 3 | 33049152 | GLB1 | 0.02123 | 0.04803 |
| rs11548666 | 3 | 150345664 | SELT,LOC677762 | 0.02141 | 0.0212 |
| rs2695681 | 15 | 55099981 |  | 0.02147 | 0.01681 |
| rs1953092 | 10 | 59799112 |  | 0.02149 | 0.0671 |
| rs2869621 | 20 | 50556670 |  | 0.02165 | 0.02268 |
| rs7175830 | 15 | 61275513 | RORA | 0.02188 | 0.04056 |
| rs13237572 | 7 | 118263380 |  | 0.02209 | 0.04308 |
| rs4243769 | 15 | 25140684 | SNRPN | 0.02222 | 0.01144 |
| rs10430123 | 1 | 46221440 | IPP,RPL6P1 | 0.02227 | 0.04649 |
| rs859141 | 12 | 25222024 | LRMP | 0.02229 | 0.03866 |
| rs12689294 | X | 13465476 |  | 0.02237 | 0.0369 |
| rs3743759 | 16 | 77224656 | MON1B,SYCE1L | 0.02239 | 0.01643 |
| rs10743138 | 11 | 10422929 | LOC100130460 | 0.02243 | 0.007784 |
| rs6888754 | 5 | 111586429 | EPB41L4A | 0.02245 | 0.04993 |
| rs11211169 | 1 | 46166709 | TMEM69,IPP | 0.02256 | 0.0358 |
| rs223612 | 6 | 143714240 |  | 0.02266 | 0.05609 |
| rs7589342 | 2 | 106433478 | NCK2 | 0.02268 | 0.0209 |
| rs1493661 | 11 | 25682233 |  | 0.02268 | 0.1585 |
| rs729005 | 8 | 126672313 |  | 0.02273 | 0.02905 |
| rs12753487 | 1 | 97733434 | DPYD | 0.02273 | 0.04815 |
| rs11618565 | 13 | 103937572 | LOC728183 | 0.02274 | 0.007668 |
| rs2271733 | 18 | 56940307 | RAX | 0.02275 | 0.04112 |
| rs12449788 | 17 | 50452607 |  | 0.02281 | 0.01254 |
| rs13204707 | 6 | 68775710 |  | 0.02293 | 0.03317 |
| rs9369298 | 6 | 41576640 | FOXP4 | 0.02297 | 0.03922 |
| rs12053259 | 2 | 106428720 | NCK2 | 0.02304 | 0.02193 |
| rs10491108 | 17 | 28793317 | CPD,GOSR1 | 0.02327 | 0.04061 |
| rs12206378 | 6 | 79876976 |  | 0.02333 | 0.02471 |
| rs846995 | 6 | 107998142 | LOC728089 | 0.02342 | 0.06635 |
| rs13408457 | 2 | 121189187 |  | 0.02359 | 0.03396 |
| rs11971136 | 7 | 45343699 |  | 0.02359 | 0.05295 |
| rs9471517 | 6 | 12341753 |  | 0.02362 | 0.0159 |
| rs10932919 | 2 | 222421725 | EPHA4,LOC729770,RPL23P5 | 0.02363 | 0.1057 |
| rs2471512 | 12 | 88453608 | C12orf29,CEP290 | 0.02388 | 0.02012 |
| rs582285 | 11 | 117362948 | DSCAML1 | 0.02394 | 0.01196 |
| rs7112550 | 11 | 128656157 | FLI1 | 0.02396 | 0.03091 |
| rs1500639 | 15 | 98047533 |  | 0.02416 | 0.02211 |
| rs12770651 | 10 | 81670940 | LOC100132402,MBL1P | 0.02425 | 0.02321 |
| rs10199004 | 2 | 143244498 |  | 0.02443 | 0.04821 |
| rs10931982 | 2 | 202832130 |  | 0.02455 | 0.02902 |
| rs1511771 | 18 | 42102847 |  | 0.02458 | 0.06985 |
| rs836610 | 2 | 173521724 | LOC100129169 | 0.02461 | 0.006282 |
| rs5005414 | 18 | 38728795 |  | 0.02467 | 0.04624 |
| rs12956050 | 18 | 44935801 |  | 0.02472 | 0.01092 |
| rs10249433 | 7 | 19397441 |  | 0.02472 | 0.04547 |
| rs7018983 | 9 | 4303682 | GLIS3 | 0.02483 | 0.02274 |
| rs2658645 | 10 | 59727834 | MRPS35P3 | 0.02496 | 0.07288 |
| rs721367 | 13 | 95546650 |  | 0.02501 | 0.4237 |
| rs9283670 | 4 | 41758803 | PHOX2B | 0.02513 | 0.03522 |
| rs4381764 | 2 | 202126863 | CASP8 | 0.02534 | 0.06869 |
| rs13173675 | 5 | 161568643 | GABRG2 | 0.02541 | 0.01232 |
| rs2136492 | 2 | 221704949 |  | 0.02541 | 0.01793 |
| rs7798109 | 7 | 121482703 | LOC100131285 | 0.02542 | 0.04075 |
| rs2322100 | 18 | 5477770 | EPB41L3 | 0.02559 | 0.01642 |
| rs16828502 | 2 | 151496151 |  | 0.02562 | 0.03926 |
| rs12359953 | 10 | 33358070 | RPL7AP53 | 0.02562 | 0.08793 |
| rs12601304 | 17 | 68629948 |  | 0.02563 | 0.01744 |
| rs16960029 | 16 | 58315125 | CCDC113,KLKBL4 | 0.02574 | 0.1156 |
| rs10826846 | 10 | 30985081 | hCG_1783494,LOC731954 | 0.02576 | 0.1449 |
| rs3000049 | 14 | 84903941 |  | 0.02592 | 0.04563 |
| rs9366483 | 6 | 22911301 |  | 0.02606 | 0.0845 |
| rs9809435 | 3 | 141023325 | ACPL2,ZBTB38 | 0.02613 | 0.01765 |
| rs9612827 | 22 | 25403765 | KIAA1671 | 0.02624 | 0.03526 |
| rs2009259 | 15 | 25416057 | SNRPN,SNORD115-1,SNORD115@,SNORD115-2,SNORD115-3,SNORD115-4,SNORD115-5,SNORD115-6,SNORD115-7,SNORD115-8,SNORD115-9,SNORD115-10,SNORD115-11 | 0.02625 | 0.04932 |
| rs12672417 | 7 | 98701971 | SMURF1 | 0.02629 | 0.06667 |
| rs4599370 | 4 | 169641509 | PALLD | 0.02643 | 0.0604 |
| rs12073135 | 1 | 65192187 | RAVER2 | 0.0265 | 0.05573 |
| rs12447155 | 16 | 50945544 |  | 0.02664 | 0.0103 |
| rs2713721 | 3 | 187541227 |  | 0.02668 | 0.03005 |
| rs10438246 | 14 | 105410183 | AHNAK2 | 0.02691 | 0.01145 |
| rs12852636 | X | 96402056 | DIAPH2 | 0.02693 | 0.03243 |
| rs1569020 | 12 | 131574485 | GPR133 | 0.02706 | 0.03805 |
| rs11794849 | 9 | 127204712 | GPR144 | 0.02712 | 0.003371 |
| rs2346412 | 2 | 46718989 | LOC388946 | 0.02714 | 0.00988 |
| rs12677136 | 8 | 118879130 | EXT1 | 0.02727 | 0.03099 |
| rs2114961 | 5 | 134574232 |  | 0.02741 | 0.05942 |
| rs17354986 | 1 | 20585773 |  | 0.02763 | 0.01936 |
| rs985545 | 7 | 19330700 |  | 0.02789 | 0.06745 |
| rs7553194 | 1 | 11864149 | MTHFR,CLCN6 | 0.02799 | 0.01265 |
| rs10972554 | 9 | 35661379 | SIT1,RMRP,CCDC107,C9orf100,CA9 | 0.02815 | 0.01488 |
| rs721173 | 2 | 13907633 |  | 0.02829 | 0.1223 |
| rs17274 | 7 | 142224511 | TRB@,TRBV10-3,TRBV11-3,TRBV12-3,TRBV12-4 | 0.02832 | 0.02331 |
| rs2104871 | X | 56070928 |  | 0.02836 | 0.01084 |
| rs6065460 | 20 | 40929694 | PTPRT | 0.02857 | 0.05436 |
| rs4677138 | 3 | 72430169 |  | 0.02867 | 0.02486 |
| rs2232316 | 2 | 169757562 | SPC25,G6PC2 | 0.02875 | 0.06715 |
| rs510441 | 1 | 156129968 | SEMA4A | 0.02894 | 0.08428 |
| rs1498994 | 3 | 20944820 |  | 0.02904 | 0.04506 |
| rs4646885 | 1 | 165658598 | ALDH9A1,LOC440700 | 0.02908 | 0.03881 |
| rs11809079 | 1 | 100914270 | CDC14A | 0.02947 | 0.05859 |
| rs2611093 | 11 | 34327576 | ABTB2 | 0.02948 | 0.01328 |
| rs3935256 | 18 | 64900038 |  | 0.02978 | 0.02106 |
| rs6033553 | 20 | 12922766 |  | 0.02993 | 0.009904 |
| rs7493080 | 14 | 69738079 | GALNTL1 | 0.03005 | 0.04618 |
| rs3742023 | 12 | 109693982 | ACACB | 0.03007 | 0.01963 |
| rs17693908 | 8 | 134021212 | TG | 0.03017 | 0.04063 |
| rs604814 | 1 | 10567122 | PEX14 | 0.03037 | 0.007126 |
| rs4073397 | 2 | 10162037 | UNQ5830 | 0.03041 | 0.01681 |
| rs4300357 | 11 | 26720410 | SLC5A12 | 0.03058 | 0.0371 |
| rs6573854 | 14 | 69167738 | RPL12P7 | 0.03061 | 0.01578 |
| rs11177370 | 12 | 69175793 |  | 0.03067 | 0.02595 |
| rs868407 | 1 | 201341341 | TNNT2,LAD1 | 0.03081 | 0.05829 |
| rs990626 | 2 | 169997051 | LRP2 | 0.03102 | 0.02596 |
| rs6094342 | 20 | 45080916 | ZNF663,ZNF840P | 0.03118 | 0.04976 |
| rs4751149 | 10 | 131727813 | EBF3 | 0.03119 | 0.01803 |
| rs4861168 | 4 | 42218575 |  | 0.0312 | 0.08526 |
| rs2364445 | 2 | 31176516 | GALNT14 | 0.03125 | 0.06633 |
| rs11628718 | 14 | 47697802 | MDGA2 | 0.03129 | 0.0506 |
| rs32636 | 5 | 14574119 | FAM105A | 0.03132 | 0.01902 |
| rs6943498 | 7 | 137767051 | AKR1D1 | 0.03138 | 0.04792 |
| rs688853 | 10 | 6489773 | PRKCQ | 0.03159 | 0.007527 |
| rs1002548 | 5 | 8523982 |  | 0.03162 | 0.04963 |
| rs12261088 | 10 | 23317917 | ARMC3 | 0.03178 | 0.0397 |
| rs4331766 | 4 | 23563103 | LOC643751 | 0.03185 | 0.0742 |
| rs6604644 | 1 | 216940621 | ESRRG | 0.0319 | 0.0308 |
| rs2329449 | 7 | 39587912 | C7orf36 | 0.03193 | 0.03142 |
| rs3768748 | 2 | 46351848 | PRKCE | 0.03195 | 0.12 |
| rs252353 | 16 | 29182179 |  | 0.03206 | 0.01418 |
| rs728546 | 16 | 69455528 | LOC100130763,CYB5B | 0.03242 | 0.01937 |
| rs10160 | 22 | 19026025 | LOC729314,LOC402036,LOC100129262,DGCR2 | 0.03247 | 0.01885 |
| rs2236194 | 20 | 61879009 | BIRC7,NKAIN4 | 0.03248 | 0.03284 |
| rs11649209 | 16 | 77334791 | ADAMTS18 | 0.03265 | 0.03136 |
| rs4935347 | 10 | 54540783 | MBL2 | 0.03267 | 0.01659 |
| rs613444 | 9 | 13682827 |  | 0.03267 | 0.02881 |
| rs6550227 | 3 | 19976253 | EFHB,LOC402125,RAB5A | 0.03272 | 0.004675 |
| rs16960032 | 16 | 58315581 | CCDC113,KLKBL4 | 0.03272 | 0.1393 |
| rs6464238 | 7 | 152231321 |  | 0.03298 | 0.01611 |
| rs12456141 | 18 | 26667607 |  | 0.03299 | 0.03362 |
| rs3922844 | 3 | 38624253 | SCN5A | 0.03303 | 0.02011 |
| rs541476 | 21 | 44795084 |  | 0.03306 | 0.04593 |
| rs609341 | 11 | 117341808 | DSCAML1 | 0.03312 | 0.0825 |
| rs6802930 | 3 | 126912432 | C3orf56 | 0.03324 | 0.04438 |
| rs940428 | 7 | 10518762 | LOC100128638 | 0.03326 | 0.03172 |
| rs7861175 | 9 | 113857337 |  | 0.03339 | 0.02903 |
| rs1560579 | 2 | 137875975 | THSD7B | 0.03345 | 0.01225 |
| rs12261843 | 10 | 35554054 | CCNY | 0.03349 | 0.0472 |
| rs7724372 | 5 | 102885995 | LOC100129962,NUDT12 | 0.0335 | 0.09237 |
| rs837948 | 12 | 125002525 | NCOR2 | 0.03357 | 0.03658 |
| rs355438 | 5 | 77224214 |  | 0.03362 | 0.1263 |
| rs11930133 | 4 | 42184374 |  | 0.03368 | 0.01862 |
| rs4677760 | 3 | 194553182 |  | 0.03372 | 0.03715 |
| rs6892131 | 5 | 164448888 |  | 0.03392 | 0.04062 |
| rs1149580 | 11 | 76549513 |  | 0.03412 | 0.05662 |
| rs10520813 | 15 | 96664707 | LOC100132798 | 0.03425 | 0.04305 |
| rs7329638 | 13 | 36736953 | SOHLH2 | 0.03438 | 0.02121 |
| rs1019661 | 4 | 14188591 |  | 0.03469 | 0.04384 |
| rs281792 | 2 | 200850378 |  | 0.03477 | 0.1328 |
| rs7071452 | 10 | 117821350 | GFRA1 | 0.03487 | 0.03788 |
| rs17561086 | 11 | 106398020 |  | 0.03525 | 0.02559 |
| rs12220128 | 10 | 94975011 | LOC387703 | 0.03556 | 0.01869 |
| rs11163047 | 1 | 81007960 |  | 0.03573 | 0.3679 |
| rs11692302 | 2 | 10993273 |  | 0.03613 | 0.03805 |
| rs6444277 | 3 | 187997999 | LPP | 0.03615 | 0.02486 |
| rs9815082 | 3 | 65702885 | MAGI1 | 0.0363 | 0.09155 |
| rs11729484 | 4 | 190552585 |  | 0.03631 | 0.07111 |
| rs3822056 | 4 | 187187005 | KLKB1,F11 | 0.03634 | 0.09184 |
| rs11646806 | 16 | 51641705 |  | 0.03639 | 0.04775 |
| rs2205264 | 8 | 117011944 |  | 0.03643 | 0.04015 |
| rs6117279 | 20 | 698987 |  | 0.0365 | 0.08655 |
| rs11759524 | 6 | 23078899 |  | 0.03658 | 0.04799 |
| rs3758012 | 8 | 1847184 | ARHGEF10 | 0.03689 | 0.03967 |
| rs693196 | 9 | 15202417 | TTC39B | 0.03701 | 0.06346 |
| rs1458018 | 5 | 95253295 | ELL2 | 0.03701 | 0.09195 |
| rs7221792 | 17 | 73834690 | LOC100129946,UNC13D,WBP2 | 0.03717 | 0.08424 |
| rs11701969 | 21 | 36372962 | RUNX1 | 0.03722 | 0.1281 |
| rs9672365 | 15 | 38206270 |  | 0.03758 | 0.02097 |
| rs2036707 | 13 | 108484454 | FAM155A | 0.03759 | 0.01673 |
| rs11807250 | 1 | 112123946 | ADORA3 | 0.03762 | 0.03831 |
| rs13337397 | 16 | 75295639 | BCAR1,LOC100131601 | 0.03764 | 0.03096 |
| rs288447 | 10 | 80359650 |  | 0.0389 | 0.05168 |
| rs10009093 | 4 | 143629934 | INPP4B | 0.03915 | 0.01577 |
| rs453573 | 20 | 15564114 | MACROD2 | 0.03917 | 0.02465 |
| rs1025949 | 12 | 20508587 | LOC100131677,PDE3A | 0.03933 | 0.01692 |
| rs1838697 | 5 | 13972255 |  | 0.03954 | 0.04137 |
| rs1805731 | 12 | 9095226 | PHC1,M6PR | 0.03967 | 0.05794 |
| rs16944877 | 12 | 115459615 |  | 0.03967 | 0.1724 |
| rs13249365 | 8 | 21275297 |  | 0.03978 | 0.05051 |
| rs4370456 | 7 | 118571724 |  | 0.03985 | 0.1294 |
| rs1481803 | 8 | 72056169 |  | 0.03992 | 0.03697 |
| rs12571712 | 10 | 54501332 |  | 0.0401 | 0.05456 |
| rs12208915 | 6 | 79702735 | PHIP | 0.04015 | 0.04179 |
| rs7679673 | 4 | 106061534 | TET2 | 0.04022 | 0.04211 |
| rs4868222 | 5 | 172282241 | ERGIC1 | 0.04042 | 0.04451 |
| rs10122597 | 9 | 114363693 | PTGR1,C9orf29 | 0.04067 | 0.1335 |
| rs6724972 | 2 | 174452807 | LOC100129456 | 0.04087 | 0.1144 |
| rs868567 | 10 | 129266910 |  | 0.04088 | 0.03997 |
| rs13233773 | 7 | 57503666 | ZNF716 | 0.04123 | 0.00555 |
| rs4827690 | X | 145190852 |  | 0.04153 | 0.04475 |
| rs6113334 | 20 | 21706490 | PAX1 | 0.0416 | 0.08172 |
| rs1421296 | 13 | 106259210 |  | 0.04174 | 0.03867 |
| rs4677029 | 3 | 71230143 | FOXP1 | 0.04177 | 0.03809 |
| rs13405823 | 2 | 165213471 |  | 0.04188 | 0.05953 |
| rs6973895 | 7 | 138566810 | KIAA1549 | 0.04191 | 0.02201 |
| rs9878609 | 3 | 72305546 |  | 0.04194 | 0.06148 |
| rs9536317 | 13 | 53494250 |  | 0.04244 | 0.01469 |
| rs10508689 | 10 | 25522280 | GPR158 | 0.04244 | 0.118 |
| rs11714198 | 3 | 157248336 | C3orf55 | 0.04286 | 0.04213 |
| rs7133666 | 12 | 18443809 | PIK3C2G | 0.04289 | 0.04559 |
| rs10049894 | 4 | 11767121 | LOC644753 | 0.0429 | 0.01361 |
| rs1043823 | 1 | 201845432 | IPO9,SHISA4,LOC100128471 | 0.04315 | 0.102 |
| rs274304 | 10 | 25247006 | PRTFDC1 | 0.04321 | 0.03044 |
| rs9435429 | 1 | 107526857 |  | 0.04327 | 0.01292 |
| rs237899 | 3 | 8808515 | OXTR | 0.04345 | 0.02367 |
| rs6577014 | 2 | 96076409 | FAHD2A,LOC653186 | 0.04352 | 0.03759 |
| rs4331558 | 2 | 42081856 | RPS12P4 | 0.04389 | 0.03175 |
| rs16927139 | 8 | 62198366 | CLVS1 | 0.0439 | 0.1474 |
| rs657816 | 1 | 30919030 |  | 0.04391 | 0.02417 |
| rs17005693 | 4 | 123281293 | KIAA1109,ADAD1 | 0.04392 | 0.02599 |
| rs2416329 | 5 | 113023119 |  | 0.04398 | 0.08843 |
| rs11787522 | 8 | 18372093 |  | 0.04398 | 0.1417 |
| rs17315781 | 5 | 37140209 | C5orf42 | 0.04405 | 0.04844 |
| rs4663692 | 2 | 237908632 |  | 0.04419 | 0.374 |
| rs7948759 | 11 | 12098277 |  | 0.04422 | 0.06842 |
| rs3777018 | 5 | 82626919 | XRCC4 | 0.04423 | 0.1743 |
| rs12368075 | 12 | 94293569 | LOC100129881 | 0.04437 | 0.05624 |
| rs7718303 | 5 | 119342045 |  | 0.04438 | 0.2099 |
| rs6088466 | 20 | 32913534 |  | 0.04451 | 0.06771 |
| rs2826 | 11 | 76733847 | ACER3,B3GNT6 | 0.04472 | 0.04589 |
| rs10883617 | 10 | 103113035 | BTRC | 0.04483 | 0.06432 |
| rs7119361 | 11 | 76573235 | ACER3 | 0.04493 | 0.02884 |
| rs10824493 | 10 | 78914597 | KCNMA1 | 0.04496 | 0.04832 |
| rs8026050 | 15 | 45142419 | SORD2 | 0.04496 | 0.1234 |
| rs770382 | 13 | 51141404 | LOC730194 | 0.04526 | 0.04329 |
| rs1846922 | 15 | 55069363 |  | 0.04537 | 0.03812 |
| rs1865550 | 8 | 131772999 |  | 0.04573 | 0.1355 |
| rs7033444 | 9 | 6901428 | KDM4C | 0.04577 | 0.02854 |
| rs7324897 | 13 | 75038000 |  | 0.04588 | 0.1145 |
| rs6908441 | 6 | 143480743 | AIG1 | 0.04601 | 0.03154 |
| rs9395145 | 6 | 45987653 | CLIC5 | 0.04626 | 0.3104 |
| rs1472258 | 7 | 146431210 | CNTNAP2 | 0.04635 | 0.07377 |
| rs5751239 | 22 | 42592239 | TCF20 | 0.04675 | 0.02961 |
| rs6868555 | 5 | 76919925 | OTP | 0.04682 | 0.01619 |
| rs10202378 | 2 | 185926550 |  | 0.04698 | 0.1627 |
| rs7693642 | 4 | 126986023 |  | 0.04708 | 0.03288 |
| rs1771492 | 1 | 245728083 | KIF26B | 0.04711 | 0.05505 |
| rs1440153 | 3 | 98489686 | ST3GAL6 | 0.04716 | 0.05148 |
| rs12077763 | 1 | 77679574 | PIGK | 0.04726 | 0.02844 |
| rs1572364 | 10 | 25085931 |  | 0.04732 | 0.04695 |
| rs951760 | 1 | 219862348 |  | 0.04753 | 0.09737 |
| rs7669896 | 4 | 119510402 | LOC729218 | 0.04757 | 0.08856 |
| rs28472312 | 16 | 28826049 | ATXN2L | 0.04788 | 0.0926 |
| rs1820238 | 16 | 58319225 | CCDC113,KLKBL4 | 0.04815 | 0.126 |
| rs1978628 | 16 | 77444613 | ADAMTS18 | 0.04842 | 0.08708 |
| rs6473677 | 8 | 52915994 |  | 0.04848 | 0.0298 |
| rs297565 | 8 | 96180926 |  | 0.04852 | 0.04011 |
| rs1040166 | 2 | 159606437 | LOC100131270 | 0.04858 | 0.06481 |
| rs11150996 | 18 | 74763117 | MBP,LOC100131698 | 0.04864 | 0.04124 |
| rs2973820 | 5 | 97049480 |  | 0.0487 | 0.07788 |
| rs17205561 | 15 | 62439284 |  | 0.04875 | 0.06232 |
| rs708838 | 12 | 116262914 |  | 0.04914 | 0.03041 |
| rs9358435 | 6 | 21497929 |  | 0.04967 | 0.03415 |
| rs1191812 | 2 | 20224999 | MATN3,LAPTM4A | 0.05002 | 0.05563 |
| rs12568891 | 1 | 61835924 | NFIA | 0.05076 | 0.07289 |
| rs12637098 | 3 | 194551660 |  | 0.05079 | 0.04153 |
| rs3782638 | 12 | 5834164 | ANO2 | 0.05093 | 0.07582 |
| rs7154970 | 14 | 71064577 | MED6 | 0.05105 | 0.03829 |
| rs2032700 | 3 | 179040177 | ZNF639 | 0.05121 | 0.05528 |
| rs17722526 | 15 | 40320267 | EIF2AK4,SRP14 | 0.05144 | 0.04008 |
| rs6708858 | 2 | 10098227 | GRHL1 | 0.05155 | 0.06391 |
| rs4827384 | X | 66068591 |  | 0.05157 | 0.3676 |
| rs2506842 | 6 | 138414419 | PERP | 0.05179 | 0.1111 |
| rs4482016 | 11 | 40918036 |  | 0.05221 | 0.05229 |
| rs10944578 | 6 | 92954254 |  | 0.05228 | 0.07398 |
| rs1909655 | 10 | 69378540 | CTNNA3 | 0.05253 | 0.163 |
| rs1873933 | 8 | 27417422 | GULOP | 0.05266 | 0.02743 |
| rs761878 | 22 | 50249677 | ZBED4 | 0.05273 | 0.1076 |
| rs4803995 | 19 | 47218599 | PRKD2,STRN4 | 0.05276 | 0.2933 |
| rs2272300 | 12 | 53587468 | CSAD,ZNF740,ITGB7 | 0.05307 | 0.05899 |
| rs1997739 | 22 | 26580172 | PITPNB | 0.05333 | 0.1034 |
| rs11713769 | 3 | 167901202 |  | 0.05337 | 0.07117 |
| rs1998249 | 10 | 102780638 | LOC100132914,PDZD7,SFXN3 | 0.05361 | 0.1245 |
| rs3735494 | 7 | 45124286 | CCM2,NACAD,LOC100128364 | 0.0539 | 0.0467 |
| rs2252902 | 8 | 11499277 |  | 0.05393 | 0.01919 |
| rs7145831 | 14 | 45458324 | FAM179B | 0.05429 | 0.05249 |
| rs265030 | 4 | 96385457 | UNC5C | 0.05454 | 0.0376 |
| rs10828351 | 10 | 23140731 |  | 0.05458 | 0.03876 |
| rs9949617 | 18 | 20879217 | C18orf45 | 0.05474 | 0.03486 |
| rs1821271 | 5 | 107263596 | FBXL17 | 0.0549 | 0.1766 |
| rs12658846 | 5 | 52064120 | PELO | 0.05495 | 0.05564 |
| rs12916632 | 15 | 55118942 |  | 0.05519 | 0.02231 |
| rs561811 | 17 | 781407 | NXN | 0.0552 | 0.07317 |
| rs1028598 | 2 | 33260019 | LTBP1 | 0.05528 | 0.08189 |
| rs12476097 | 2 | 175666865 | CHN1 | 0.05529 | 0.09249 |
| rs1467972 | 15 | 93245286 | LOC643790,UNQ6190 | 0.05529 | 0.1165 |
| rs4129743 | 18 | 75679215 |  | 0.05534 | 0.03099 |
| rs849511 | 2 | 206518838 |  | 0.05537 | 0.02373 |
| rs1728149 | 2 | 10617598 |  | 0.05545 | 0.128 |
| rs7537003 | 1 | 41032059 |  | 0.05551 | 0.1212 |
| rs1016166 | 7 | 26490833 | LOC100130672 | 0.05555 | 0.06316 |
| rs11055865 | 12 | 14390700 | LOC644693 | 0.05557 | 0.1279 |
| rs12562937 | 1 | 2229478 | SKI | 0.0556 | 0.08391 |
| rs17666079 | 10 | 26310679 | MYO3A | 0.05565 | 0.06371 |
| rs17522918 | 1 | 45987574 | PRDX1,LOC100128639 | 0.05595 | 0.02421 |
| rs17573298 | 16 | 78218016 | WWOX | 0.056 | 0.09015 |
| rs12518099 | 5 | 89546109 |  | 0.05604 | 0.02788 |
| rs12267038 | 10 | 13994618 | FRMD4A | 0.05619 | 0.07055 |
| rs7032778 | 9 | 316117 | DOCK8 | 0.0564 | 0.02965 |
| rs17236010 | 15 | 33688687 | RYR3 | 0.05647 | 0.0562 |
| rs4471960 | 20 | 8450247 | PLCB1 | 0.0565 | 0.04801 |
| rs13079502 | 3 | 66772425 |  | 0.05653 | 0.02742 |
| rs3744819 | 18 | 13882486 | MC2R | 0.05653 | 0.0364 |
| rs260118 | 15 | 99679830 | SYNM,TTC23 | 0.05663 | 0.04709 |
| rs7102076 | 11 | 106394204 |  | 0.05675 | 0.02392 |
| rs12633064 | 3 | 53061377 | SFMBT1,LOC401068 | 0.05718 | 0.1424 |
| rs844569 | 6 | 147912444 |  | 0.05725 | 0.07018 |
| rs11772988 | 7 | 31825367 | PDE1C | 0.0573 | 0.04038 |
| rs4873289 | 8 | 49697516 |  | 0.05735 | 0.04586 |
| rs958825 | 1 | 53759072 | LRP8 | 0.05772 | 0.03918 |
| rs3741278 | 11 | 117352949 | DSCAML1 | 0.05779 | 0.01266 |
| rs17610165 | 8 | 15061346 | SGCZ | 0.05783 | 0.05198 |
| rs10733510 | 9 | 4316407 | GLIS3 | 0.05817 | 0.08838 |
| rs11740678 | 5 | 111597275 | EPB41L4A | 0.05857 | 0.09415 |
| rs3846716 | 5 | 112059594 | APC | 0.05864 | 0.07443 |
| rs12059342 | 1 | 172307593 | DNM3 | 0.05867 | 0.1169 |
| rs4673317 | 2 | 206160453 | PARD3B | 0.05948 | 0.04321 |
| rs7528905 | 1 | 78456355 | FUBP1,DNAJB4 | 0.05951 | 0.2167 |
| rs17005544 | 12 | 79785690 | SYT1 | 0.05981 | 0.1004 |
| rs11143908 | 9 | 76921043 |  | 0.05988 | 0.04163 |
| rs2973996 | 5 | 119182542 |  | 0.06017 | 0.1118 |
| rs5967983 | X | 81714458 |  | 0.06043 | 0.1482 |
| rs6470602 | 8 | 129099503 | PVT1 | 0.06066 | 0.07651 |
| rs7754833 | 6 | 126190894 | NCOA7 | 0.06071 | 0.07987 |
| rs1830175 | 19 | 22382080 | ZNF676,LOC100128854 | 0.06077 | 0.07556 |
| rs9917642 | 3 | 149026545 |  | 0.06083 | 0.03098 |
| rs2823569 | 21 | 17406282 |  | 0.06163 | 0.09953 |
| rs11950485 | 5 | 124386991 |  | 0.06172 | 0.05851 |
| rs2067061 | 20 | 42196550 | SGK2 | 0.06186 | 0.02927 |
| rs2609240 | 7 | 34684184 | NPSR1 | 0.06194 | 0.1362 |
| rs12186409 | 5 | 87208971 |  | 0.06239 | 0.2706 |
| rs1622043 | 6 | 21609285 | LOC645301 | 0.06252 | 0.05216 |
| rs7232794 | 18 | 61185752 |  | 0.06298 | 0.1028 |
| rs7863623 | 9 | 21548586 |  | 0.06301 | 0.1039 |
| rs2776271 | 21 | 37327654 |  | 0.06304 | 0.2131 |
| rs4409444 | 9 | 9225463 | PTPRD | 0.06308 | 0.05076 |
| rs1019113 | 17 | 32548821 |  | 0.06324 | 0.1096 |
| rs7944964 | 11 | 96938440 |  | 0.06363 | 0.03823 |
| rs6419085 | 2 | 18286852 |  | 0.06373 | 0.0768 |
| rs2924907 | 8 | 2731382 |  | 0.06379 | 0.05404 |
| rs1773158 | 3 | 194334197 | TMEM44,LOC100132805 | 0.06441 | 0.1019 |
| rs6752228 | 2 | 161547697 |  | 0.06454 | 0.07404 |
| rs35794740 | 13 | 52996183 | THSD1,VPS36 | 0.06455 | 0.13 |
| rs7328678 | 13 | 75026129 |  | 0.06456 | 0.2063 |
| rs1276322 | 18 | 20921129 | C18orf45 | 0.06483 | 0.05842 |
| rs10894799 | 11 | 134208908 | GLB1L2,LOC100130507 | 0.06493 | 0.01861 |
| rs6764920 | 3 | 164267629 |  | 0.06532 | 0.04433 |
| rs360144 | 11 | 9788673 | LOC283104 | 0.06547 | 0.8615 |
| rs12710697 | 2 | 19320968 |  | 0.06571 | 0.06303 |
| rs11653777 | 17 | 28575158 | SLC6A4,BLMH | 0.06587 | 0.08869 |
| rs6935203 | 6 | 144434024 | SF3B5 | 0.06631 | 0.07416 |
| rs6817420 | 4 | 23927720 |  | 0.06656 | 0.08697 |
| rs1187280 | 9 | 87416825 | NTRK2 | 0.06671 | 0.1184 |
| rs10004074 | 4 | 139837873 | LOC100128578 | 0.06678 | 0.08032 |
| rs12559228 | X | 113585221 |  | 0.06761 | 0.0732 |
| rs7831232 | 8 | 139768859 | COL22A1 | 0.06773 | 0.04046 |
| rs11082705 | 18 | 46352562 | KIAA0427 | 0.06825 | 0.07891 |
| rs4765529 | 12 | 127797208 |  | 0.06844 | 0.08838 |
| rs7255003 | 19 | 52377635 | LOC730733,LOC100127975,ZNF577 | 0.06853 | 0.05857 |
| rs11216399 | 11 | 117323389 | DSCAML1 | 0.06892 | 0.0869 |
| rs11598565 | 10 | 33540448 | NRP1 | 0.0691 | 0.05293 |
| rs1615656 | 10 | 132720292 |  | 0.06954 | 0.1436 |
| rs3094481 | 16 | 2779236 | PRSS27 | 0.06958 | 0.2641 |
| rs6050200 | 20 | 24935748 | CST7,C20orf3 | 0.06999 | 0.07234 |
| rs1160224 | 1 | 195999508 |  | 0.07023 | 0.0397 |
| rs17866121 | 7 | 90057918 |  | 0.07112 | 0.08078 |
| rs10900771 | 5 | 123787030 |  | 0.07113 | 0.05446 |
| rs2058641 | 7 | 12173903 |  | 0.07178 | 0.142 |
| rs2194539 | 10 | 82561579 |  | 0.07184 | 0.1207 |
| rs1125065 | 3 | 43176153 |  | 0.07235 | 0.09969 |
| rs6657417 | 1 | 164497528 |  | 0.07242 | 0.08011 |
| rs1214896 | 12 | 72943972 | TRHDE | 0.07279 | 0.03509 |
| rs954509 | 7 | 45368159 |  | 0.07351 | 0.2147 |
| rs9398863 | 6 | 128320912 | PTPRK | 0.07361 | 0.08748 |
| rs10846681 | 12 | 125003705 | NCOR2 | 0.07374 | 0.1546 |
| rs1327556 | 10 | 112721408 | SHOC2 | 0.07395 | 0.09042 |
| rs11111712 | 12 | 104081723 | STAB2 | 0.07396 | 0.242 |
| rs1398777 | 3 | 145581255 |  | 0.07424 | 0.08998 |
| rs1147474 | 14 | 69781395 | GALNTL1 | 0.07429 | 0.05524 |
| rs4915221 | 1 | 201166383 | LOC440706,IGFN1 | 0.07477 | 0.1574 |
| rs12528998 | 6 | 50664180 | TFAP2D | 0.07549 | 0.07771 |
| rs1403739 | 15 | 61318450 | RORA | 0.07551 | 0.03537 |
| rs2029319 | 4 | 27229288 | FLJ45721 | 0.07577 | 0.06348 |
| rs218966 | 7 | 11022230 | PHF14 | 0.07585 | 0.04887 |
| rs10759180 | 9 | 109162816 |  | 0.07605 | 0.0455 |
| rs1864649 | 17 | 54087622 |  | 0.07605 | 0.1515 |
| rs8010431 | 14 | 86334363 |  | 0.0764 | 0.06374 |
| rs7706772 | 5 | 111628901 | EPB41L4A | 0.07659 | 0.07648 |
| rs6490971 | 13 | 25235807 | ATP12A | 0.07669 | 0.07304 |
| rs1148247 | 10 | 35496946 | CREM | 0.07679 | 0.1345 |
| rs535529 | 18 | 11562631 |  | 0.07686 | 0.2401 |
| rs12517454 | 5 | 82052937 |  | 0.07696 | 0.1025 |
| rs761586 | 6 | 110653700 | C6orf186 | 0.07714 | 0.05026 |
| rs10416242 | 19 | 13611618 |  | 0.07761 | 0.05965 |
| rs4597355 | 17 | 14912004 |  | 0.07799 | 0.4223 |
| rs2642682 | 5 | 38046081 |  | 0.07813 | 0.1304 |
| rs16904150 | 8 | 130762837 | GSDMC | 0.07831 | 0.08377 |
| rs239728 | 21 | 28702427 |  | 0.07835 | 0.2004 |
| rs4631934 | 12 | 63705313 |  | 0.07869 | 0.09522 |
| rs17142427 | 7 | 20149537 |  | 0.07897 | 0.2345 |
| rs2449222 | 8 | 3483411 | CSMD1 | 0.07905 | 0.2328 |
| rs13060971 | 3 | 167105744 | ZBBX | 0.0792 | 0.1755 |
| rs2254379 | 3 | 127937326 | EEFSEC | 0.07924 | 0.09673 |
| rs13183439 | 5 | 126298272 | 3-Mar | 0.07941 | 0.07266 |
| rs4876951 | 9 | 92290874 | UNQ6494,LOC100129600 | 0.07965 | 0.06389 |
| rs4461172 | 18 | 68845292 |  | 0.08001 | 0.08569 |
| rs713607 | 13 | 104750929 |  | 0.08015 | 0.1301 |
| rs4824184 | X | 22343597 |  | 0.08022 | 0.08053 |
| rs2272668 | 8 | 76463571 | HNF4G | 0.0808 | 0.1421 |
| rs13187021 | 5 | 110949080 |  | 0.08104 | 0.0415 |
| rs2681992 | 15 | 55113486 |  | 0.08106 | 0.05068 |
| rs2511636 | 8 | 105178819 | RIMS2 | 0.08126 | 0.07708 |
| rs12905568 | 15 | 100389182 |  | 0.08137 | 0.1631 |
| rs12638863 | 3 | 150533002 |  | 0.08147 | 0.0783 |
| rs1961415 | 13 | 22484460 |  | 0.08154 | 0.06636 |
| rs353325 | 5 | 40210933 |  | 0.08203 | 0.0571 |
| rs9967806 | 2 | 219878756 | LOC100129175,MIR375,hsa-mir-375,CCDC108 | 0.08216 | 0.2535 |
| rs2842573 | 1 | 52380808 | RAB3B | 0.08219 | 0.03136 |
| rs1740698 | 14 | 93548738 | ITPK1 | 0.0823 | 0.1202 |
| rs10487676 | 7 | 43535765 | HECW1 | 0.08253 | 0.1887 |
| rs11648451 | 16 | 74868518 |  | 0.08261 | 0.1287 |
| rs1652713 | 10 | 77112300 |  | 0.0827 | 0.05747 |
| rs17489259 | 2 | 98392302 | TMEM131 | 0.08277 | 0.1732 |
| rs1870563 | 5 | 168103440 | SLIT3 | 0.08279 | 0.03645 |
| rs12546664 | 8 | 92823082 |  | 0.0834 | 0.1043 |
| rs10923260 | 1 | 117818497 |  | 0.08355 | 0.03787 |
| rs11013262 | 10 | 23319824 | ARMC3 | 0.08364 | 0.1097 |
| rs6879661 | 5 | 53532777 | ARL15 | 0.08369 | 0.1535 |
| rs6562825 | 13 | 75022317 |  | 0.08378 | 0.1833 |
| rs7782106 | 7 | 155856121 |  | 0.08381 | 0.1459 |
| rs1326172 | 6 | 76741709 | IMPG1 | 0.08384 | 0.05301 |
| rs860554 | 1 | 201262432 | PKP1 | 0.08389 | 0.1359 |
| rs7701036 | 5 | 85093571 |  | 0.08419 | 0.03911 |
| rs17088003 | 4 | 67972601 |  | 0.08474 | 0.09245 |
| rs4123361 | 16 | 22812364 | HS3ST2 | 0.08512 | 0.1705 |
| rs2090380 | 4 | 153986325 |  | 0.0852 | 0.0416 |
| rs2469795 | 17 | 3164801 | OR1D3P | 0.08526 | 0.05083 |
| rs7592040 | 2 | 26741551 | OTOF | 0.08536 | 0.02481 |
| rs10264186 | 7 | 129786406 | LOC100128325 | 0.08546 | 0.1605 |
| rs2077911 | 14 | 94983224 | SERPINA12 | 0.0855 | 0.07089 |
| rs12375351 | 8 | 131659295 |  | 0.08567 | 0.0983 |
| rs9848416 | 3 | 156239255 | KCNAB1 | 0.08582 | 0.1562 |
| rs4968857 | 17 | 67420433 | MAP2K6 | 0.08633 | 0.1032 |
| rs7588190 | 2 | 123525591 |  | 0.0867 | 0.1161 |
| rs751714 | 1 | 244964797 |  | 0.0867 | 0.3183 |
| rs519887 | 2 | 169780885 | ABCB11 | 0.08687 | 0.1918 |
| rs10508312 | 10 | 6814474 |  | 0.0871 | 0.1759 |
| rs7307991 | 12 | 8995694 | A2ML1 | 0.08729 | 0.1425 |
| rs2223059 | 8 | 117129410 |  | 0.08736 | 0.135 |
| rs3016390 | 11 | 132616073 | OPCML | 0.0874 | 0.1565 |
| rs6598219 | 12 | 132039699 |  | 0.08747 | 0.2434 |
| rs1265224 | 6 | 4514319 |  | 0.08751 | 0.1539 |
| rs12773202 | 10 | 3838993 | KLF6 | 0.0877 | 0.1522 |
| rs17120184 | 14 | 48922262 |  | 0.08797 | 0.1202 |
| rs2468239 | 12 | 88444560 | C12orf29,CEP290 | 0.08806 | 0.0539 |
| rs4401444 | 4 | 82620131 |  | 0.0887 | 0.08337 |
| rs9649183 | 7 | 93674925 |  | 0.08877 | 0.2306 |
| rs11737402 | 4 | 88291439 | HSD17B11 | 0.08959 | 0.1248 |
| rs10498581 | 14 | 85736868 | RNU3P3 | 0.09074 | 0.09825 |
| rs3771395 | 2 | 71133014 | VAX2 | 0.09075 | 0.152 |
| rs3745550 | 19 | 7115573 | LOC100131165,INSR | 0.09082 | 0.05678 |
| rs17058290 | 13 | 61174931 |  | 0.09097 | 0.1 |
| rs2238253 | 14 | 72638048 | RGS6 | 0.09105 | 0.08516 |
| rs13069451 | 3 | 35537731 |  | 0.09141 | 0.06393 |
| rs6711988 | 2 | 106080746 | LOC728966 | 0.09209 | 0.5072 |
| rs2007560 | 13 | 45388967 | LOC144817 | 0.09215 | 0.1971 |
| rs13041173 | 20 | 32542814 | LOC100128033 | 0.09253 | 0.2619 |
| rs10877092 | 12 | 58686243 |  | 0.09272 | 0.1516 |
| rs12368065 | 12 | 56946240 | RBMS2 | 0.09332 | 0.0944 |
| rs8073958 | 17 | 4384778 | SPNS3,SPNS2 | 0.09359 | 0.0612 |
| rs7823798 | 8 | 122462289 |  | 0.09374 | 0.03631 |
| rs1523060 | 3 | 182450613 |  | 0.09376 | 0.2534 |
| rs6050664 | 20 | 2605308 | TMC2 | 0.09382 | 0.1736 |
| rs268804 | 14 | 58039592 | SLC35F4 | 0.09389 | 0.1135 |
| rs9476916 | 6 | 9804986 |  | 0.09399 | 0.09308 |
| rs2895814 | 8 | 83323041 |  | 0.09409 | 0.1208 |
| rs11746854 | 5 | 78829154 | HOMER1,RPL7AP32 | 0.09412 | 0.08272 |
| rs11639717 | 16 | 676989 | RAB40C,LOC100132363,WFIKKN1,C16orf13,TRNAG-CCC,FAM195A | 0.09417 | 0.07008 |
| rs4851735 | 2 | 105786077 |  | 0.09534 | 0.1554 |
| rs11061382 | 12 | 131694265 | LOC116437 | 0.09565 | 0.1606 |
| rs11074015 | 15 | 92270337 |  | 0.09567 | 0.2799 |
| rs522594 | 9 | 13682981 |  | 0.09573 | 0.1651 |
| rs7765284 | 6 | 85777822 |  | 0.09574 | 0.1576 |
| rs10062421 | 5 | 177652884 | AGXT2L2 | 0.09589 | 0.01788 |
| rs10082014 | 1 | 61456362 |  | 0.09608 | 0.1471 |
| rs4131141 | 1 | 51528697 |  | 0.09642 | 0.07553 |
| rs9639217 | 7 | 15045529 | LOC100128217 | 0.09658 | 0.1327 |
| rs3733197 | 4 | 102839287 | BANK1 | 0.09685 | 0.08742 |
| rs6897774 | 5 | 26409352 |  | 0.09685 | 0.21 |
| rs931702 | 3 | 112934752 | BOC | 0.09748 | 0.1394 |
| rs13314357 | 3 | 136487559 | STAG1 | 0.09772 | 0.08407 |
| rs4777372 | 15 | 71643787 | THSD4,RPL17P39 | 0.09786 | 0.2016 |
| rs12516704 | 5 | 52833426 |  | 0.09816 | 0.2872 |
| rs10853838 | 19 | 52166835 | SIGLEC14 | 0.09833 | 0.09671 |
| rs2042290 | 19 | 55013771 | LAIR2 | 0.09845 | 0.07073 |
| rs6981943 | 8 | 83315422 |  | 0.0991 | 0.09927 |
| rs1307279 | 9 | 87281307 | NTRK2 | 0.09924 | 0.06632 |
| rs6500042 | 16 | 59559290 |  | 0.09931 | 0.2248 |
| rs17145648 | 16 | 8333541 |  | 0.1005 | 0.1752 |
| rs12601673 | 17 | 50805330 |  | 0.1012 | 0.0895 |
| rs8058150 | 16 | 7977528 |  | 0.1013 | 0.1407 |
| rs6427191 | 1 | 169389288 | C1orf114 | 0.1021 | 0.2055 |
| rs1571662 | 21 | 28503077 |  | 0.1022 | 0.09753 |
| rs1398944 | 4 | 90057814 |  | 0.1024 | 0.06217 |
| rs10187088 | 2 | 127463952 | GYPC | 0.1027 | 0.03653 |
| rs2790552 | 14 | 49134069 |  | 0.1028 | 0.1073 |
| rs10281731 | 7 | 155661335 |  | 0.1031 | 0.1819 |
| rs9964644 | 18 | 8178318 | PTPRM | 0.1035 | 0.1329 |
| rs1935581 | 10 | 90195149 | RNLS | 0.1039 | 0.07193 |
| rs7554095 | 1 | 87681151 |  | 0.104 | 0.3198 |
| rs705904 | 16 | 11306397 | LOC729954 | 0.1042 | 0.05847 |
| rs7311897 | 12 | 9020328 | A2ML1 | 0.1043 | 0.07454 |
| rs3824633 | 10 | 16874666 | RSU1 | 0.1052 | 0.06555 |
| rs11617386 | 13 | 69599261 |  | 0.1053 | 0.102 |
| rs1620996 | 6 | 21602552 | SOX4,LOC645301 | 0.1054 | 0.1122 |
| rs742903 | X | 141105942 |  | 0.1061 | 0.09169 |
| rs2441921 | 8 | 105234363 | RIMS2 | 0.107 | 0.08203 |
| rs7781650 | 7 | 103669828 |  | 0.1071 | 0.07473 |
| rs785475 | 1 | 46609736 | PIK3R3,LOC100133124 | 0.1077 | 0.08136 |
| rs2343468 | 2 | 45569103 |  | 0.1081 | 0.07247 |
| rs10799000 | 1 | 34340467 | CSMD2 | 0.1083 | 0.07498 |
| rs9884208 | 4 | 61363382 |  | 0.1087 | 0.1373 |
| rs2078778 | 9 | 136938961 | BRD3,ARF4P1 | 0.1087 | 0.1399 |
| rs1352075 | 11 | 69457293 | CCND1 | 0.1089 | 0.1972 |
| rs2465785 | 7 | 52161793 |  | 0.1092 | 0.09674 |
| rs2164851 | 2 | 78302215 |  | 0.1094 | 0.08734 |
| rs748666 | 15 | 96400537 |  | 0.1097 | 0.3923 |
| rs910612 | 6 | 43950706 | C6orf223 | 0.1098 | 0.1181 |
| rs1884644 | 20 | 6029634 | LRRN4 | 0.11 | 0.121 |
| rs4737483 | 8 | 58878786 |  | 0.1104 | 0.07606 |
| rs410763 | 6 | 33007111 |  | 0.1107 | 0.2618 |
| rs4939551 | 18 | 46139548 | KIAA0427 | 0.1108 | 0.2462 |
| rs2206586 | 1 | 14359224 |  | 0.1109 | 0.09465 |
| rs16903246 | 5 | 87855403 | LOC645323 | 0.1113 | 0.3441 |
| rs17399334 | 2 | 58690295 | LOC644456 | 0.1116 | 0.06066 |
| rs9494600 | 6 | 137241328 | PEX7,SLC35D3 | 0.1116 | 0.1391 |
| rs10225656 | 7 | 97221135 |  | 0.1119 | 0.1462 |
| rs1112438 | 3 | 39152345 | GORASP1,TTC21A | 0.1121 | 0.06525 |
| rs9994690 | 4 | 80442759 |  | 0.1122 | 0.06642 |
| rs6711663 | 2 | 53549197 |  | 0.1132 | 0.1817 |
| rs4892806 | X | 111622504 | ZCCHC16,LOC643888 | 0.1134 | 0.09891 |
| rs10888742 | 1 | 52402956 | RAB3B | 0.114 | 0.07139 |
| rs7616008 | 3 | 59697659 |  | 0.1141 | 0.04791 |
| rs2506696 | 9 | 136950691 | BRD3,ARF4P1 | 0.1148 | 0.2456 |
| rs7482257 | 11 | 27755298 | BDNF | 0.1151 | 0.1811 |
| rs12338788 | 9 | 116214058 | RGS3 | 0.1153 | 0.2107 |
| rs7512337 | 1 | 185335532 |  | 0.116 | 0.1548 |
| rs1012577 | 7 | 87499470 | SLC25A40,DBF4 | 0.1165 | 0.1397 |
| rs1952964 | 14 | 32518353 |  | 0.1171 | 0.08604 |
| rs17583463 | 7 | 46264195 |  | 0.1174 | 0.1216 |
| rs1447958 | 3 | 60547602 | FHIT | 0.1174 | 0.1552 |
| rs4655383 | 1 | 213578006 |  | 0.1179 | 0.08807 |
| rs2326864 | 6 | 130094398 | LOC100130402 | 0.118 | 0.2653 |
| rs260886 | 12 | 22930568 |  | 0.1183 | 0.1052 |
| rs9904197 | 17 | 55477138 | MSI2 | 0.1198 | 0.366 |
| rs17413169 | 10 | 33533148 | NRP1 | 0.12 | 0.08939 |
| rs5932615 | X | 128468626 |  | 0.1204 | 0.04485 |
| rs10075972 | 5 | 23493956 | PRDM9 | 0.1214 | 0.08016 |
| rs4548027 | 6 | 130397515 | L3MBTL3 | 0.1217 | 0.0604 |
| rs4849733 | 2 | 119664339 |  | 0.1217 | 0.07826 |
| rs10043348 | 5 | 117007686 |  | 0.1226 | 0.1303 |
| rs431736 | 11 | 115907433 |  | 0.1227 | 0.094 |
| rs16855398 | 2 | 7544326 |  | 0.1227 | 0.1913 |
| rs1732597 | 12 | 115712201 |  | 0.1229 | 0.08549 |
| rs7883954 | X | 141009134 |  | 0.1229 | 0.1189 |
| rs642294 | 1 | 60494956 | C1orf87 | 0.123 | 0.1384 |
| rs2024366 | 7 | 154032974 | DPP6 | 0.1234 | 0.1192 |
| rs10852766 | 17 | 73951864 | FBF1,ACOX1 | 0.1239 | 0.2025 |
| rs2100516 | 1 | 8214803 |  | 0.1242 | 0.1948 |
| rs11815205 | 10 | 54060101 | PRKG1,LOC729054,DKK1 | 0.1244 | 0.122 |
| rs788912 | 4 | 73412231 | ADAMTS3 | 0.1245 | 0.2517 |
| rs9814627 | 3 | 141920434 | GK5 | 0.1254 | 0.06371 |
| rs273995 | 7 | 137629146 | CREB3L2,LOC100130880 | 0.1254 | 0.1254 |
| rs6550108 | 3 | 32275125 | LOC100129194,CMTM8,KRT18P15 | 0.1254 | 0.2043 |
| rs17462259 | 4 | 47574737 | ATP10D | 0.1258 | 0.1019 |
| rs12434438 | 14 | 62197298 | HIF1A | 0.1262 | 0.1552 |
| rs500713 | 11 | 117320976 | DSCAML1 | 0.1262 | 0.2615 |
| rs1387292 | 9 | 11254070 |  | 0.1263 | 0.1539 |
| rs12687590 | X | 115853187 |  | 0.1267 | 0.3589 |
| rs1078756 | 14 | 104061646 | C14orf153 | 0.1277 | 0.1955 |
| rs9511399 | 13 | 25236232 | ATP12A | 0.1281 | 0.1704 |
| rs2332211 | 4 | 172133937 |  | 0.1284 | 0.362 |
| rs6968993 | 7 | 49931275 | VWC2,LOC100128734 | 0.1288 | 0.07624 |
| rs10991552 | 9 | 107877624 |  | 0.1297 | 0.1943 |
| rs1446553 | 2 | 149201775 | MBD5 | 0.1298 | 0.2332 |
| rs2894634 | 7 | 111722814 | DOCK4 | 0.1307 | 0.1807 |
| rs1241719 | 14 | 92673710 |  | 0.1308 | 0.06352 |
| rs9603965 | 13 | 113491759 | ATP11A | 0.1308 | 0.1071 |
| rs4130404 | 8 | 99420915 | LOC100131849,KCNS2 | 0.131 | 0.1136 |
| rs10276363 | 7 | 142191647 | TRB@,TRBV11-2,TRBV12-2,TRBV6-5,TRBV7-4 | 0.1311 | 0.1964 |
| rs10275423 | 7 | 103905649 |  | 0.1316 | 0.1135 |
| rs6918870 | 6 | 76277296 |  | 0.1317 | 0.3634 |
| rs8017965 | 14 | 86335746 |  | 0.1323 | 0.1026 |
| rs1258236 | 10 | 50916484 | C10orf53 | 0.1325 | 0.09076 |
| rs17017866 | 2 | 80007727 | CTNNA2 | 0.1325 | 0.3947 |
| rs2430212 | X | 117088056 | KLHL13 | 0.1332 | 0.3575 |
| rs10174809 | 2 | 173386534 |  | 0.1334 | 0.07323 |
| rs3129678 | 13 | 22547878 |  | 0.1341 | 0.2959 |
| rs10510946 | 3 | 65817691 | MAGI1 | 0.1345 | 0.2708 |
| rs11634632 | 15 | 61912189 |  | 0.1347 | 0.1209 |
| rs17163588 | 1 | 26450009 | PDIK1L | 0.1349 | 0.0829 |
| rs1440006 | 2 | 190612339 | ANKAR,OSGEPL1 | 0.1349 | 0.1555 |
| rs12206701 | 6 | 4615655 | KU-MEL-3 | 0.1351 | 0.1545 |
| rs17482717 | 1 | 165663798 | ALDH9A1,LOC440700 | 0.1354 | 0.1503 |
| rs17105227 | 14 | 77513800 | C14orf4 | 0.1357 | 0.07276 |
| rs7762514 | 6 | 4462171 |  | 0.1357 | 0.2844 |
| rs238416 | 19 | 45857049 | RPS16P9,KLC3,ERCC2 | 0.1361 | 0.0161 |
| rs11767887 | 7 | 46310371 |  | 0.1363 | 0.08726 |
| rs7610241 | 3 | 65706257 | MAGI1 | 0.1366 | 0.191 |
| rs1388615 | 3 | 62226704 | PTPRG | 0.1368 | 0.1898 |
| rs7966147 | 12 | 39562350 |  | 0.1372 | 0.1134 |
| rs667932 | 4 | 17165360 |  | 0.1372 | 0.1892 |
| rs373707 | 19 | 56439340 | NLRP13,NLRP8 | 0.1373 | 0.2281 |
| rs198646 | 6 | 143156612 | HIVEP2 | 0.1379 | 0.3632 |
| rs973720 | 6 | 23013575 |  | 0.1384 | 0.04867 |
| rs17272364 | 8 | 54155807 | OPRK1 | 0.1384 | 0.1135 |
| rs1386354 | 2 | 173011537 |  | 0.1385 | 0.2529 |
| rs228188 | 6 | 38318373 | BTBD9 | 0.1388 | 0.09924 |
| rs11078432 | 17 | 2952430 |  | 0.1388 | 0.1198 |
| rs10046 | 15 | 51502986 | CYP19A1 | 0.1388 | 0.1896 |
| rs180727 | 10 | 117727166 |  | 0.14 | 0.1668 |
| rs4815747 | 20 | 4821622 | RASSF2 | 0.141 | 0.2125 |
| rs2125576 | 5 | 167084107 |  | 0.1413 | 0.1273 |
| rs1542715 | 11 | 26027235 |  | 0.1416 | 0.2065 |
| rs17095577 | 14 | 98028428 | LOC730217 | 0.1418 | 0.2172 |
| rs7623720 | 3 | 149280435 | WWTR1,LOC100128025 | 0.1426 | 0.1966 |
| rs2583008 | 2 | 182572502 |  | 0.1428 | 0.1523 |
| rs4434528 | 7 | 15337929 | TMEM195 | 0.1431 | 0.2174 |
| rs482700 | 4 | 116067490 | MRPS33P3 | 0.1435 | 0.1582 |
| rs17553945 | 15 | 95517815 |  | 0.1436 | 0.08123 |
| rs187372 | 16 | 84351828 | WFDC1 | 0.1436 | 0.2927 |
| rs4502701 | 4 | 122905041 |  | 0.1445 | 0.4431 |
| rs6805866 | 3 | 141402137 | LOC646730 | 0.1449 | 0.03387 |
| rs2658636 | 10 | 59718844 | LOC100128586 | 0.145 | 0.1728 |
| rs9381129 | 6 | 42040291 | TAF8 | 0.1452 | 0.08177 |
| rs2761845 | 13 | 51294332 | DLEU7 | 0.1452 | 0.1134 |
| rs1550226 | 15 | 61505679 | RORA | 0.1453 | 0.06898 |
| rs1336405 | 10 | 85261363 |  | 0.1454 | 0.183 |
| rs1495922 | 2 | 241216359 |  | 0.1454 | 0.2613 |
| rs1790834 | 18 | 71948257 | CYB5A | 0.1456 | 0.2295 |
| rs16829064 | 3 | 158247867 | RSRC1 | 0.1456 | 0.2635 |
| rs883133 | 10 | 121188960 | GRK5 | 0.1457 | 0.1651 |
| rs12606371 | 18 | 57365624 | CCBE1 | 0.1458 | 0.2158 |
| rs12795565 | 11 | 10511142 | AMPD3 | 0.1463 | 0.06975 |
| rs1729761 | 13 | 95752225 | ABCC4 | 0.1463 | 0.08686 |
| rs2494506 | 1 | 159829535 | VSIG8 | 0.1464 | 0.05537 |
| rs700513 | 4 | 73409668 | ADAMTS3 | 0.1465 | 0.2956 |
| rs6726744 | 2 | 106653926 |  | 0.1466 | 0.16 |
| rs16915601 | 12 | 19693016 |  | 0.147 | 0.6394 |
| rs6969328 | 7 | 155882479 | tcag7.1213 | 0.1479 | 0.1034 |
| rs17080711 | 18 | 66924242 |  | 0.148 | 0.1364 |
| rs4468331 | 11 | 3165412 | OSBPL5,LOC100133241 | 0.1482 | 0.05805 |
| rs615756 | 12 | 114117760 |  | 0.1487 | 0.2263 |
| rs6127280 | 20 | 53396845 |  | 0.1499 | 0.7409 |
| rs1562812 | 15 | 96513174 |  | 0.1503 | 0.2339 |
| rs10933018 | 2 | 224669429 | AP1S3 | 0.1505 | 0.1947 |
| rs10491467 | 5 | 119586814 |  | 0.1515 | 0.2554 |
| rs2916 | 1 | 67216697 | SGIP1,TCTEX1D1 | 0.1516 | 0.1256 |
| rs3757574 | 7 | 45148019 | NACAD,TBRG4,SNORA5A,SNORA5C,SNORA5B | 0.1518 | 0.1954 |
| rs10139118 | 14 | 26563796 |  | 0.152 | 0.1318 |
| rs9948324 | 18 | 48349191 | MRO | 0.1522 | 0.2745 |
| rs6746636 | 2 | 53538984 |  | 0.1523 | 0.1203 |
| rs2477764 | 10 | 3270102 |  | 0.1534 | 0.1845 |
| rs1499244 | 11 | 22798431 | GAS2 | 0.1534 | 0.2429 |
| rs974529 | 2 | 137870529 | THSD7B | 0.1546 | 0.05919 |
| rs4818789 | 21 | 46948827 | SLC19A1,LOC100130597 | 0.1549 | 0.3895 |
| rs12133002 | 1 | 237416049 | RYR2 | 0.1554 | 0.4366 |
| rs12636212 | 3 | 86205223 |  | 0.1555 | 0.1872 |
| rs9840651 | 3 | 177067339 |  | 0.156 | 0.07863 |
| rs13155773 | 5 | 64773576 | ADAMTS6 | 0.1561 | 0.6224 |
| rs1466266 | 11 | 5115856 | OR52S1P,OR52E3P,OR52J1P | 0.1567 | 0.1689 |
| rs16861406 | 2 | 174097106 | ZAK | 0.158 | 0.0778 |
| rs11893616 | 2 | 199167804 |  | 0.1588 | 0.05013 |
| rs1152484 | 14 | 56791252 |  | 0.1589 | 0.2375 |
| rs1007036 | 1 | 212951364 | NSL1,TATDN3 | 0.1598 | 0.1011 |
| rs10258322 | 7 | 46261337 |  | 0.1599 | 0.2852 |
| rs17378869 | 13 | 23931733 | SACS | 0.1603 | 0.2828 |
| rs2072293 | 14 | 74537885 | C14orf45,ALDH6A1,LIN52 | 0.1605 | 0.1604 |
| rs6040121 | 20 | 10726917 |  | 0.1606 | 0.09879 |
| rs459644 | 21 | 46015300 | C21orf29,KRTAP10-5,KRTAP10-6,KRTAP10-7,KRTAP10-8 | 0.1612 | 0.059 |
| rs2293150 | 3 | 150285664 | LOC100129720,EIF2A | 0.1612 | 0.1199 |
| rs7986186 | 13 | 82423704 |  | 0.1612 | 0.1644 |
| rs1795819 | 12 | 55002014 | PPP1R1A,GLYCAM1 | 0.1619 | 0.1501 |
| rs919933 | 2 | 34616306 |  | 0.1621 | 0.116 |
| rs4724137 | 7 | 4919277 | RADIL,PAPOLB | 0.1627 | 0.463 |
| rs2902883 | 19 | 52186517 | MIR99B,hsa-mir-99b,MIRLET7E,hsa-let-7e,MIR125A,hsa-mir-125a,NCRNA00085 | 0.1628 | 0.2474 |
| rs837493 | 12 | 125075660 |  | 0.1634 | 0.5002 |
| rs4691121 | 4 | 165392008 |  | 0.1637 | 0.2446 |
| rs11855184 | 15 | 51807464 | DMXL2 | 0.1639 | 0.2453 |
| rs7462518 | 8 | 143620393 | BAI1 | 0.164 | 0.3513 |
| rs12701888 | 7 | 41417008 |  | 0.165 | 0.1663 |
| rs4075908 | 19 | 55941827 | LOC284296,SHISA7 | 0.1653 | 0.05878 |
| rs343032 | 7 | 35501213 |  | 0.1655 | 0.3417 |
| rs2004484 | 8 | 74267120 | LOC100127988 | 0.1666 | 0.2323 |
| rs11216386 | 11 | 116815199 | DSCAML1 | 0.167 | 0.2722 |
| rs3931230 | 15 | 92974636 | ST8SIA2 | 0.1672 | 0.2055 |
| rs1927412 | 13 | 77933618 |  | 0.1673 | 0.2588 |
| rs12198732 | 6 | 87149869 | LOC643926 | 0.1677 | 0.3022 |
| rs2702934 | 8 | 6707885 | XKR5 | 0.1679 | 0.2233 |
| rs6570048 | 6 | 136198594 | PDE7B | 0.1688 | 0.1786 |
| rs13021124 | 2 | 231539173 |  | 0.169 | 0.2376 |
| rs2415713 | 14 | 42635007 |  | 0.1706 | 0.06086 |
| rs17840238 | 6 | 61969533 |  | 0.1707 | 0.05461 |
| rs324324 | 18 | 38358755 |  | 0.1708 | 0.2614 |
| rs984430 | 9 | 87582827 | NTRK2 | 0.1716 | 0.09269 |
| rs10775435 | 18 | 5460319 | EPB41L3 | 0.172 | 0.2374 |
| rs1934775 | 6 | 9831530 |  | 0.1728 | 0.2886 |
| rs589916 | 11 | 114063362 | ZBTB16 | 0.173 | 0.1586 |
| rs2211720 | 8 | 36760400 | KCNU1 | 0.1735 | 0.324 |
| rs12931375 | 16 | 55467633 |  | 0.1736 | 0.1302 |
| rs12145073 | 1 | 85394258 | MCOLN2 | 0.1741 | 0.1974 |
| rs1598705 | 3 | 66729080 |  | 0.1753 | 0.1083 |
| rs4716306 | 6 | 10106467 | RPL7AP36 | 0.1753 | 0.1761 |
| rs9523144 | 13 | 91509067 |  | 0.1755 | 0.1999 |
| rs6682683 | 1 | 46342337 | MAST2 | 0.1758 | 0.2297 |
| rs5920018 | X | 145228381 |  | 0.176 | 0.1527 |
| rs6644910 | XY | 1503037 | IL3RA,SLC25A6,LOC729629,NCRNA00105 | 0.1766 | 0.279 |
| rs507647 | 13 | 27063614 |  | 0.1769 | 0.209 |
| rs9401746 | 6 | 124764631 | NKAIN2 | 0.1777 | 0.1252 |
| rs2108961 | 17 | 11584698 | DNAH9 | 0.1779 | 0.1908 |
| rs4938013 | 11 | 113264470 | ANKK1 | 0.1804 | 0.07647 |
| rs2303632 | 12 | 108918330 | FICD,SART3 | 0.1805 | 0.1963 |
| rs2230658 | 1 | 46078854 | NASP,CCDC17 | 0.1806 | 0.2189 |
| rs5915630 | X | 5973034 | NLGN4X | 0.1808 | 0.1779 |
| rs2459855 | 11 | 91295509 |  | 0.1809 | 0.1707 |
| rs263811 | 13 | 55563725 |  | 0.1811 | 0.0821 |
| rs6436465 | 2 | 224976957 |  | 0.1815 | 0.4289 |
| rs17353315 | 2 | 222185903 |  | 0.1816 | 0.2375 |
| rs4327688 | 6 | 23890877 |  | 0.182 | 0.2198 |
| rs1531321 | 21 | 24639297 |  | 0.1827 | 0.265 |
| rs2567456 | 15 | 96345844 |  | 0.1828 | 0.2539 |
| rs17616063 | 16 | 51436882 |  | 0.1834 | 0.2251 |
| rs4736598 | 8 | 133649465 | LRRC6 | 0.1834 | 0.3416 |
| rs1870795 | 4 | 91246656 | FAM190A | 0.184 | 0.1632 |
| rs209350 | 5 | 161510796 | GABRG2 | 0.1842 | 0.0933 |
| rs2835167 | 21 | 37311117 |  | 0.1846 | 0.5673 |
| rs10995571 | 10 | 51588177 | NCOA4,TIMM23 | 0.1848 | 0.2551 |
| rs1372045 | 11 | 76563014 | ACER3 | 0.1852 | 0.1954 |
| rs2223579 | 1 | 176453688 | PAPPA2 | 0.1853 | 0.1796 |
| rs6464401 | 7 | 154009325 | DPP6 | 0.1856 | 0.2606 |
| rs537756 | 11 | 96016054 | MAML2 | 0.186 | 0.2637 |
| rs10427677 | 22 | 35460650 | ISX | 0.1863 | 0.2472 |
| rs9961807 | 18 | 53819904 |  | 0.1864 | 0.1848 |
| rs9386887 | 6 | 110647920 | C6orf186 | 0.1868 | 0.1956 |
| rs9434618 | 1 | 8200578 |  | 0.1874 | 0.3276 |
| rs10262336 | 7 | 80660357 |  | 0.1874 | 0.6257 |
| rs2675380 | 5 | 58093288 | RAB3C | 0.1886 | 0.1801 |
| rs1762506 | 1 | 107656356 |  | 0.19 | 0.1992 |
| rs2324599 | 13 | 40920004 |  | 0.1901 | 0.1388 |
| rs10502070 | 11 | 106339209 |  | 0.1908 | 0.1447 |
| rs7474747 | 10 | 54545202 | MBL2 | 0.1913 | 0.1638 |
| rs10779763 | 1 | 11690766 |  | 0.1921 | 0.1946 |
| rs7030211 | 9 | 74378277 | TMEM2 | 0.1924 | 0.2232 |
| rs11097280 | 4 | 91860898 |  | 0.1927 | 0.1377 |
| rs12043489 | 1 | 169301175 | NME7 | 0.1927 | 0.3527 |
| rs12090448 | 1 | 82203562 |  | 0.1931 | 0.1974 |
| rs2450286 | 8 | 50831539 | LOC100127998,SNTG1 | 0.1931 | 0.2787 |
| rs11165976 | 1 | 98755006 |  | 0.1939 | 0.2806 |
| rs2288856 | 19 | 56879974 |  | 0.194 | 0.1456 |
| rs17102710 | 12 | 66982114 | GRIP1 | 0.194 | 0.1689 |
| rs10411547 | 19 | 2617422 | GNG7 | 0.1943 | 0.4218 |
| rs3784563 | 15 | 74290993 | STOML1,PML | 0.1953 | 0.2595 |
| rs1379465 | 2 | 6659744 | LOC391349 | 0.1956 | 0.1926 |
| rs518216 | 1 | 46037357 | AKR1A1,NASP | 0.1967 | 0.2295 |
| rs8010676 | 14 | 88568270 |  | 0.1968 | 0.5866 |
| rs7674341 | 4 | 181375630 |  | 0.1969 | 0.1211 |
| rs2236580 | 1 | 214026324 |  | 0.1972 | 0.2962 |
| rs9864170 | 3 | 85610908 |  | 0.1973 | 0.2419 |
| rs6712896 | 2 | 45707814 | SRBD1 | 0.1979 | 0.2944 |
| rs1022234 | 4 | 123563078 |  | 0.198 | 0.2419 |
| rs2064504 | 20 | 44403710 | WFDC3,DNTTIP1 | 0.1985 | 0.1311 |
| rs966061 | 13 | 77178812 |  | 0.199 | 0.1694 |
| rs6980919 | 8 | 5651425 |  | 0.1992 | 0.2761 |
| rs2219265 | 3 | 66942668 |  | 0.1994 | 0.1939 |
| rs7096316 | 10 | 108216760 |  | 0.2003 | 0.09426 |
| rs2156384 | 21 | 24652281 |  | 0.201 | 0.2218 |
| rs10505941 | 12 | 24694019 | SOX5 | 0.2012 | 0.5246 |
| rs11699172 | 20 | 42979553 | R3HDML,HNF4A | 0.2016 | 0.249 |
| rs12380608 | 9 | 6900255 | KDM4C | 0.2025 | 0.1613 |
| rs17716313 | 8 | 95564340 | KIAA1429 | 0.2029 | 0.6787 |
| rs11660291 | 18 | 45522194 |  | 0.2039 | 0.2252 |
| rs7463426 | 8 | 31523972 | NRG1 | 0.2043 | 0.1881 |
| rs7300680 | 12 | 130299753 | TMEM132D | 0.2052 | 0.3133 |
| rs11854320 | 15 | 90903502 | GABARAPL3,ZNF774,LOC100130560 | 0.2054 | 0.1048 |
| rs10087922 | 8 | 135082117 |  | 0.2055 | 0.3826 |
| rs1477451 | 2 | 118448374 |  | 0.2064 | 0.3969 |
| rs4361562 | 5 | 2075565 |  | 0.2069 | 0.1947 |
| rs4310858 | 16 | 25761510 | HS3ST4 | 0.2079 | 0.2068 |
| rs12134651 | 1 | 211351513 |  | 0.209 | 0.2425 |
| rs11667211 | 19 | 1839483 | REXO1 | 0.2092 | 0.2276 |
| rs5989802 | XY | 1499770 | IL3RA,SLC25A6,LOC729629 | 0.2106 | 0.3001 |
| rs1205083 | 14 | 72289633 |  | 0.2113 | 0.2891 |
| rs3920611 | 6 | 67272216 |  | 0.2113 | 0.4385 |
| rs6686663 | 1 | 79835066 |  | 0.2129 | 0.1494 |
| rs4678610 | 3 | 33582575 | CLASP2 | 0.2132 | 0.3131 |
| rs9316663 | 13 | 22554991 |  | 0.2145 | 0.23 |
| rs9642649 | 7 | 154423082 | DPP6 | 0.2148 | 0.2187 |
| rs679557 | 19 | 56669758 | ZNF444,GALP | 0.215 | 0.06072 |
| rs1508334 | 9 | 32179169 |  | 0.215 | 0.1437 |
| rs16879109 | 5 | 7792917 | ADCY2 | 0.2154 | 0.06431 |
| rs2754330 | 9 | 17554647 |  | 0.2162 | 0.304 |
| rs7233189 | 18 | 5480541 | EPB41L3 | 0.2166 | 0.2034 |
| rs4972932 | 2 | 228601389 | SLC19A3 | 0.217 | 0.5152 |
| rs1926145 | 10 | 129571206 |  | 0.2174 | 0.3113 |
| rs11078053 | 17 | 12082742 |  | 0.2178 | 0.1981 |
| rs12539828 | 7 | 129157420 |  | 0.2189 | 0.6045 |
| rs4953028 | 2 | 44101827 | ABCG8 | 0.2199 | 0.1938 |
| rs11056858 | 12 | 16437515 | SLC15A5 | 0.2201 | 0.1398 |
| rs293372 | 15 | 89661746 | ABHD2 | 0.2208 | 0.2272 |
| rs1013150 | X | 12932441 | TLR8 | 0.2214 | 0.1645 |
| rs6659102 | 1 | 176535567 | PAPPA2 | 0.2219 | 0.1201 |
| rs2676070 | 15 | 33690502 | RYR3 | 0.222 | 0.2322 |
| rs17303366 | 8 | 76346061 |  | 0.2222 | 0.1188 |
| rs6774925 | 3 | 163572907 |  | 0.2222 | 0.4194 |
| rs6802413 | 3 | 81227042 |  | 0.2224 | 0.3128 |
| rs12902333 | 15 | 24335853 |  | 0.2225 | 0.3326 |
| rs1288688 | 3 | 71412654 | FOXP1 | 0.2234 | 0.2741 |
| rs7213025 | 17 | 50834441 |  | 0.2258 | 0.1921 |
| rs6790010 | 3 | 109987098 |  | 0.2258 | 0.2631 |
| rs10426177 | 19 | 56269096 | NLRP9,RFPL4A,LOC729974,LOC646663 | 0.2259 | 0.2342 |
| rs1786776 | 18 | 35087457 | BRUNOL4 | 0.2262 | 0.2043 |
| rs12809620 | 12 | 33135050 | ASS1P14 | 0.2265 | 0.343 |
| rs1496721 | 15 | 93073841 |  | 0.2269 | 0.2535 |
| rs6496469 | 15 | 88735310 | NTRK3 | 0.2295 | 0.2449 |
| rs4919480 | 10 | 102464133 |  | 0.2297 | 0.1245 |
| rs12456203 | 18 | 24160328 | KCTD1,CIAPIN1P | 0.2297 | 0.2537 |
| rs651377 | 5 | 51996032 |  | 0.2304 | 0.2499 |
| rs740965 | 7 | 121513561 | PTPRZ1 | 0.2329 | 0.3894 |
| rs6974145 | 7 | 35536636 |  | 0.2333 | 0.3006 |
| rs2972474 | 3 | 115420104 | GAP43 | 0.2334 | 0.1462 |
| rs6483557 | 11 | 18976674 | LOC645415 | 0.2337 | 0.4832 |
| rs8103033 | 19 | 40170053 | LOC400696 | 0.2341 | 0.3135 |
| rs2577703 | 2 | 23592219 | KLHL29 | 0.2348 | 0.2087 |
| rs9849625 | 3 | 148540154 | CPB1 | 0.2353 | 0.1507 |
| rs9468304 | 6 | 11042165 | ELOVL2 | 0.2356 | 0.162 |
| rs11738392 | 5 | 84021150 |  | 0.2359 | 0.403 |
| rs7662337 | 4 | 67615172 |  | 0.2366 | 0.1426 |
| rs2728487 | 7 | 47209007 |  | 0.2372 | 0.3756 |
| rs925717 | 12 | 79379050 |  | 0.2374 | 0.1557 |
| rs3792747 | 5 | 95767920 | PCSK1 | 0.2376 | 0.2976 |
| rs256563 | 2 | 190731832 | PMS1 | 0.2379 | 0.413 |
| rs2842463 | 6 | 76009426 | TMEM30A,FILIP1 | 0.2382 | 0.3292 |
| rs16865682 | 1 | 41006154 | ZNF684 | 0.2388 | 0.2231 |
| rs5966170 | X | 145450143 |  | 0.2389 | 0.352 |
| rs1421144 | 9 | 86378767 | GKAP1 | 0.2394 | 0.1417 |
| rs1861519 | 10 | 72496131 | ADAMTS14 | 0.2395 | 0.2682 |
| rs11631304 | 15 | 93060990 |  | 0.2399 | 0.2176 |
| rs738603 | 22 | 49396413 |  | 0.2418 | 0.202 |
| rs4490524 | 4 | 117252491 |  | 0.2422 | 0.2458 |
| rs6433017 | 2 | 151728587 |  | 0.2436 | 0.3014 |
| rs3782637 | 12 | 5833530 | ANO2 | 0.2457 | 0.3083 |
| rs4898455 | X | 153154887 | L1CAM,LCAP,AVPR2 | 0.2461 | 0.1864 |
| rs11701407 | 21 | 26099440 |  | 0.2471 | 0.2239 |
| rs12106212 | 20 | 54797408 |  | 0.2476 | 0.04906 |
| rs7026685 | 9 | 109899802 |  | 0.2479 | 0.1544 |
| rs11134425 | 5 | 165833633 |  | 0.2489 | 0.3912 |
| rs787652 | 10 | 95036575 | RPL17P34 | 0.2492 | 0.1876 |
| rs1857956 | 4 | 41867049 |  | 0.2501 | 0.2155 |
| rs270678 | 6 | 104784819 |  | 0.2504 | 0.4896 |
| rs11712937 | 3 | 156800384 |  | 0.2506 | 0.1474 |
| rs2242592 | 11 | 113279430 | ANKK1,DRD2 | 0.2517 | 0.1068 |
| rs7432375 | 3 | 136288405 | STAG1 | 0.2518 | 0.202 |
| rs12456768 | 18 | 72701941 | ZNF407 | 0.2523 | 0.152 |
| rs9524558 | 13 | 95262093 | TGDS,GPR180 | 0.2526 | 0.3544 |
| rs17001789 | X | 54699330 | LOC392473 | 0.2537 | 0.3505 |
| rs2164171 | 21 | 27789058 |  | 0.2539 | 0.3705 |
| rs4798367 | 18 | 5475677 | EPB41L3 | 0.2541 | 0.161 |
| rs16860626 | 2 | 13742696 |  | 0.255 | 0.3077 |
| rs1483550 | 4 | 162925724 | FSTL5 | 0.2564 | 0.5266 |
| rs10955369 | 8 | 105949909 |  | 0.2572 | 0.2376 |
| rs16869397 | 7 | 70713870 | WBSCR17 | 0.2578 | 0.1322 |
| rs8006303 | 14 | 90137426 |  | 0.2579 | 0.3951 |
| rs10466336 | 11 | 94987033 |  | 0.2593 | 0.2611 |
| rs4509910 | 13 | 41989620 |  | 0.2605 | 0.4243 |
| rs6745975 | 2 | 239646741 |  | 0.2609 | 0.09672 |
| rs11721883 | 4 | 24397676 |  | 0.2616 | 0.1996 |
| rs950259 | 12 | 131963879 |  | 0.2617 | 0.1598 |
| rs9316866 | 13 | 22846168 |  | 0.2619 | 0.1147 |
| rs1216105 | 19 | 22703220 | LOC100128139 | 0.2633 | 0.2292 |
| rs17052054 | 3 | 52337754 | MIR135A1,hsa-mir-135a-1,DNAH1 | 0.2637 | 0.5199 |
| rs6644896 | XY | 1502582 | IL3RA,SLC25A6,LOC729629,NCRNA00105 | 0.2646 | 0.384 |
| rs10514328 | 5 | 89847694 | GPR98 | 0.2651 | 0.09941 |
| rs7334667 | 13 | 33878276 | STARD13 | 0.2666 | 0.1081 |
| rs11658347 | 17 | 1109605 |  | 0.2681 | 0.2676 |
| rs6078321 | 20 | 11739808 |  | 0.2684 | 0.2659 |
| rs763384 | 20 | 10897894 |  | 0.269 | 0.1521 |
| rs198674 | 6 | 143192850 | HIVEP2 | 0.2694 | 0.1734 |
| rs6598055 | 11 | 252318 | SIRT3,PSMD13,COX8B | 0.2706 | 0.1026 |
| rs1247767 | 10 | 78806426 | KCNMA1 | 0.2713 | 0.565 |
| rs13220323 | 6 | 160506755 | IGF2R,LOC729603 | 0.2722 | 0.1409 |
| rs1865176 | 4 | 130375622 |  | 0.2742 | 0.2799 |
| rs3935993 | 5 | 119196820 |  | 0.2742 | 0.3249 |
| rs12669706 | 7 | 121545143 | PTPRZ1 | 0.2744 | 0.3291 |
| rs4357980 | 17 | 13557909 |  | 0.2749 | 0.4233 |
| rs4976890 | 8 | 37688572 | GPR124 | 0.2752 | 0.241 |
| rs2901705 | 3 | 171098888 | TNIK | 0.2754 | 0.564 |
| rs6599229 | 3 | 38672585 | SCN5A | 0.2776 | 0.2557 |
| rs7741814 | 6 | 150590784 |  | 0.2784 | 0.3338 |
| rs11062035 | 12 | 338473 | SLC6A12,SLC6A13 | 0.2788 | 0.3741 |
| rs7800918 | 7 | 13995209 | ETV1 | 0.2794 | 0.1726 |
| rs4741277 | 9 | 13055896 | LOC100130801 | 0.2803 | 0.4785 |
| rs7236987 | 18 | 13066038 | CEP192 | 0.2809 | 0.1857 |
| rs1467317 | X | 41173758 | LOC643043,DDX3X | 0.281 | 0.3189 |
| rs7963883 | 12 | 28812198 |  | 0.2829 | 0.3025 |
| rs1846914 | 15 | 55083180 |  | 0.2831 | 0.2023 |
| rs941990 | 6 | 21658881 | LOC729161 | 0.284 | 0.327 |
| rs7257950 | 19 | 46754714 |  | 0.2841 | 0.3328 |
| rs10059061 | 5 | 95781172 | PCSK1 | 0.2866 | 0.2504 |
| rs10814278 | 9 | 3589867 |  | 0.2872 | 0.5975 |
| rs2132849 | 16 | 84975343 |  | 0.2909 | 0.1274 |
| rs4268198 | 9 | 83397162 |  | 0.2917 | 0.4055 |
| rs9589279 | 13 | 92185178 | GPC5 | 0.2921 | 0.3588 |
| rs4472787 | 1 | 247666165 | LOC644852 | 0.2934 | 0.2696 |
| rs2318345 | 8 | 139901178 | COL22A1 | 0.2934 | 0.4809 |
| rs10486908 | 7 | 82860532 |  | 0.2934 | 0.671 |
| rs1492076 | 8 | 100464671 | VPS13B | 0.2936 | 0.4186 |
| rs12713741 | 2 | 26207421 | KIF3C,LOC729769 | 0.295 | 0.3025 |
| rs7576163 | 2 | 155182433 | GALNT13 | 0.2951 | 0.09677 |
| rs4911163 | 20 | 33470694 | GGT7,ACSS2 | 0.2953 | 0.2098 |
| rs11012338 | 10 | 21070393 | NEBL | 0.2955 | 0.3047 |
| rs1427142 | 6 | 93183255 |  | 0.2964 | 0.26 |
| rs8022729 | 14 | 107225161 | IGHV3-73,C14orf99,IGHV3-74,IGHVII-74-1,IGHV3-75 | 0.2971 | 0.4035 |
| rs6801834 | 3 | 141555626 |  | 0.2981 | 0.3689 |
| rs4695849 | 4 | 174471671 | NBLA00301 | 0.2989 | 0.3814 |
| rs10929046 | 2 | 235547067 |  | 0.3004 | 0.1809 |
| rs11928074 | 3 | 195557461 | MUC4 | 0.3005 | 0.2874 |
| rs4830791 | X | 12711092 | FRMPD4 | 0.3016 | 0.1487 |
| rs1414615 | 6 | 94491539 |  | 0.3019 | 0.6243 |
| rs7689380 | 4 | 172216304 |  | 0.3021 | 0.5355 |
| rs4943346 | 13 | 36450937 | DCLK1 | 0.3022 | 0.3062 |
| rs7988265 | 13 | 103341164 | TPP2,C13orf39 | 0.3029 | 0.4827 |
| rs2119010 | 15 | 93555717 | CHD2 | 0.3055 | 0.233 |
| rs1862019 | 12 | 45991000 |  | 0.3063 | 0.3073 |
| rs2032065 | 21 | 32914159 | TIAM1,LOC150051 | 0.3064 | 0.04135 |
| rs2693694 | 14 | 99679775 | BCL11B | 0.3082 | 0.3047 |
| rs13156814 | 5 | 76979394 | TBCA | 0.3086 | 0.4863 |
| rs6439295 | 3 | 131122731 |  | 0.3088 | 0.4403 |
| rs1871916 | 5 | 113083726 |  | 0.3103 | 0.3099 |
| rs2360093 | 2 | 195389486 |  | 0.3113 | 0.4024 |
| rs11689205 | 2 | 23282771 |  | 0.3115 | 0.4818 |
| rs4610302 | 4 | 88400110 | SPARCL1 | 0.3125 | 0.5154 |
| rs4151697 | 5 | 140743661 | PCDHGA1,PCDHGA2,PCDHGA3,PCDHGB1,PCDHGA4,PCDHGB2,PCDHGA5,PCDHGB3,PCDHGA6,PCDHGA7 | 0.3126 | 0.4063 |
| rs758944 | 7 | 75953297 | YWHAG | 0.3129 | 0.4988 |
| rs12120904 | 1 | 169478042 | F5 | 0.3141 | 0.251 |
| rs12611286 | 19 | 47031583 | LOC100127977 | 0.3173 | 0.3821 |
| rs7846989 | 9 | 120482940 | TLR4 | 0.3178 | 0.4379 |
| rs12916193 | 15 | 89705728 | ABHD2 | 0.3183 | 0.2129 |
| rs12246059 | 10 | 92440481 |  | 0.3187 | 0.1165 |
| rs17114976 | 21 | 43885970 | RSPH1 | 0.3198 | 0.3641 |
| rs970282 | 5 | 175174011 |  | 0.3202 | 0.3726 |
| rs7662252 | 4 | 165262655 |  | 0.3224 | 0.6995 |
| rs2712402 | 3 | 128455073 | RAB7A | 0.3238 | 0.2944 |
| rs1382462 | 13 | 55200003 |  | 0.3242 | 0.2799 |
| rs2749527 | 14 | 94827068 | SERPINA2 | 0.3253 | 0.2516 |
| rs6860457 | 5 | 39782692 |  | 0.3259 | 0.2975 |
| rs11186162 | 10 | 92162586 |  | 0.3266 | 0.3617 |
| rs7914872 | 10 | 98817687 | SLIT1 | 0.3295 | 0.3619 |
| rs2824175 | 21 | 18384773 |  | 0.3306 | 0.4112 |
| rs17019074 | 2 | 80584769 | CTNNA2 | 0.3315 | 0.2079 |
| rs3924297 | 2 | 71738385 | DYSF | 0.3325 | 0.3022 |
| rs7049850 | X | 16697183 | CTPS2 | 0.3331 | 0.2352 |
| rs7051370 | X | 110519625 | CAPN6 | 0.3334 | 0.4814 |
| rs9865722 | 3 | 28394952 | AZI2 | 0.3341 | 0.4785 |
| rs7249323 | 19 | 13377865 | CACNA1A | 0.3345 | 0.2458 |
| rs2235976 | 14 | 95227957 | GSC | 0.3348 | 0.5766 |
| rs7591530 | 2 | 64567550 | RPL23AP37 | 0.335 | 0.241 |
| rs2441022 | 5 | 14902038 | LOC402198 | 0.335 | 0.2916 |
| rs12377888 | 9 | 3096561 |  | 0.3351 | 0.4672 |
| rs7542210 | 1 | 200662215 |  | 0.3353 | 0.4964 |
| rs12380813 | 9 | 17607554 | SH3GL2 | 0.3357 | 0.3247 |
| rs6452792 | 5 | 87792844 |  | 0.3375 | 0.4004 |
| rs11895356 | 2 | 201800167 | ORC2L | 0.3376 | 0.3326 |
| rs17049106 | 2 | 129861630 |  | 0.3381 | 0.3879 |
| rs17113403 | 14 | 43203107 |  | 0.3394 | 0.5599 |
| rs877064 | 1 | 20249510 | PLA2G2E | 0.3401 | 0.6663 |
| rs3922668 | 16 | 28992646 | SPNS1,LAT | 0.3405 | 0.523 |
| rs269876 | 5 | 22591057 | CDH12 | 0.3408 | 0.2705 |
| rs6773879 | 3 | 177063272 |  | 0.3417 | 0.2672 |
| rs11735495 | 4 | 45829948 |  | 0.3426 | 0.3426 |
| rs10509605 | 10 | 92436384 |  | 0.3437 | 0.2808 |
| rs2055012 | 21 | 19481308 |  | 0.3444 | 0.1894 |
| rs17473108 | 4 | 164463778 | 1-Mar | 0.3456 | 0.4826 |
| rs12231124 | 12 | 25954453 | LOC645233 | 0.3477 | 0.4881 |
| rs8191478 | 16 | 88877429 | CDT1,APRT,GALNS | 0.3487 | 0.4492 |
| rs6714968 | 2 | 222251031 |  | 0.3497 | 0.5779 |
| rs4810782 | 20 | 47024363 |  | 0.352 | 0.287 |
| rs1154598 | 4 | 41791492 |  | 0.3521 | 0.2542 |
| rs10772890 | 12 | 16197432 | DERA | 0.3532 | 0.2917 |
| rs4755303 | 11 | 45023786 |  | 0.3546 | 0.3047 |
| rs10833865 | 11 | 22973380 |  | 0.3554 | 0.2808 |
| rs2660369 | 12 | 124872587 | NCOR2 | 0.3555 | 0.2061 |
| rs236106 | 20 | 5960691 | MCM8 | 0.3563 | 0.2728 |
| rs7604694 | 2 | 13681063 |  | 0.3598 | 0.2973 |
| rs1813160 | 2 | 71360625 | MCEE,MPHOSPH10 | 0.3598 | 0.4397 |
| rs1347267 | 15 | 39263633 |  | 0.3599 | 0.3585 |
| rs3012761 | 9 | 134687735 |  | 0.3602 | 0.2735 |
| rs1553985 | 4 | 76554604 | CDKL2 | 0.3611 | 0.4767 |
| rs5937175 | X | 68422477 | LOC100128860 | 0.3619 | 0.1448 |
| rs11711889 | 3 | 153370666 |  | 0.3619 | 0.4118 |
| rs10482957 | 21 | 26298990 |  | 0.3661 | 0.408 |
| rs7024169 | 9 | 30308963 |  | 0.368 | 0.8953 |
| rs11614654 | 12 | 133729999 | ZNF10 | 0.3684 | 0.4985 |
| rs5992761 | 22 | 18102627 | ATP6V1E1,BCL2L13 | 0.3687 | 0.4193 |
| rs3911086 | 2 | 45575726 |  | 0.3687 | 0.4627 |
| rs1607614 | 2 | 36117253 |  | 0.3695 | 0.3797 |
| rs17051989 | 9 | 89174943 |  | 0.3701 | 0.2872 |
| rs17175251 | 6 | 80732242 | TTK | 0.3701 | 0.3973 |
| rs196631 | 7 | 36345343 | EEPD1 | 0.3725 | 0.2819 |
| rs10791132 | 11 | 131117445 |  | 0.3726 | 0.3722 |
| rs4952105 | 2 | 30407284 |  | 0.3729 | 0.3702 |
| rs11017373 | 10 | 132412890 |  | 0.3746 | 0.4552 |
| rs17118511 | 12 | 53130285 | KRT126P,LOC400036,LOC100128678 | 0.3748 | 0.6609 |
| rs9599856 | 13 | 72130769 | DACH1 | 0.3755 | 0.5845 |
| rs11964846 | 6 | 15490551 | JARID2 | 0.376 | 0.5858 |
| rs2058452 | 7 | 17731550 |  | 0.3765 | 0.382 |
| rs7327169 | 13 | 74230216 |  | 0.3768 | 0.3373 |
| rs13140777 | 4 | 105054988 |  | 0.3775 | 0.6933 |
| rs900030 | 4 | 1193832 | LOC100130872 | 0.3795 | 0.4362 |
| rs6707063 | 2 | 229656721 |  | 0.3818 | 0.3874 |
| rs12497191 | 3 | 12390135 | PPARG | 0.382 | 0.1792 |
| rs10887363 | 10 | 86525965 |  | 0.3824 | 0.3596 |
| rs2514496 | 11 | 80860642 |  | 0.3829 | 0.497 |
| rs10134663 | 14 | 86945740 |  | 0.3831 | 0.5485 |
| rs2072965 | 20 | 1146136 | PSMF1,ACTG1P3 | 0.385 | 0.6059 |
| rs324960 | 7 | 34802391 | NPSR1 | 0.3852 | 0.2773 |
| rs4345960 | 11 | 11540810 | GALNTL4 | 0.3872 | 0.3487 |
| rs1729760 | 13 | 95752024 | ABCC4 | 0.3874 | 0.2499 |
| rs875811 | 7 | 48462746 | ABCA13 | 0.3875 | 0.3436 |
| rs9554967 | 13 | 104161966 |  | 0.3882 | 0.4525 |
| rs4676588 | 3 | 38940741 | SCN11A | 0.3886 | 0.2853 |
| rs332012 | 5 | 24646826 | CDH10 | 0.3886 | 0.345 |
| rs7584695 | 2 | 235082853 |  | 0.3899 | 0.5273 |
| rs2217515 | 8 | 94404371 |  | 0.3927 | 0.3651 |
| rs6913508 | 6 | 159111946 | SYTL3 | 0.393 | 0.4389 |
| rs12460587 | 19 | 52586919 | ZNF841 | 0.3955 | 0.6187 |
| rs5753027 | 22 | 30554204 | HORMAD2 | 0.3966 | 0.4274 |
| rs3858799 | 13 | 108335873 | FAM155A | 0.3971 | 0.2309 |
| rs9566456 | 13 | 40117691 | LHFP | 0.3972 | 0.3164 |
| rs12841273 | X | 152626413 | ZNF275 | 0.3982 | 0.4412 |
| rs7151975 | 14 | 70553891 | SLC8A3 | 0.3982 | 0.5954 |
| rs1004173 | 6 | 47445017 | CD2AP | 0.3993 | 0.3819 |
| rs203075 | 17 | 49998938 | CA10 | 0.3996 | 0.2393 |
| rs993598 | 2 | 179182695 | OSBPL6 | 0.4002 | 0.373 |
| rs7597807 | 2 | 230021055 | PID1 | 0.4002 | 0.3977 |
| rs256805 | 5 | 82251838 | LOC100127911 | 0.4009 | 0.6079 |
| rs4986187 | 18 | 44872493 |  | 0.4011 | 0.4101 |
| rs10762110 | 10 | 53161158 | PRKG1 | 0.4012 | 0.326 |
| rs3759465 | 13 | 108921730 | TNFSF13B | 0.4019 | 0.4865 |
| rs320427 | 1 | 213565567 |  | 0.4022 | 0.5457 |
| rs4244347 | 10 | 103452645 | FBXW4 | 0.4079 | 0.4036 |
| rs1016815 | 1 | 175428957 | TNR | 0.4088 | 0.3749 |
| rs4446676 | 7 | 103878454 |  | 0.4114 | 0.4423 |
| rs186139 | 5 | 73816110 |  | 0.4124 | 0.465 |
| rs3777194 | 5 | 95245384 | ELL2 | 0.4129 | 0.5511 |
| rs2421812 | 1 | 171407761 | CYCSP53 | 0.4158 | 0.6773 |
| rs6529956 | X | 6480342 |  | 0.4158 | 0.9363 |
| rs4981950 | 14 | 32501255 |  | 0.4165 | 0.5062 |
| rs17368045 | 1 | 190213532 | FAM5C | 0.4168 | 0.2058 |
| rs921473 | 16 | 8328333 |  | 0.418 | 0.5696 |
| rs3862101 | 8 | 17607488 | MTUS1 | 0.4232 | 0.253 |
| rs2812385 | 1 | 231864002 | DISC1 | 0.4233 | 0.4859 |
| rs2192884 | 2 | 206545465 | NRP2 | 0.4249 | 0.07202 |
| rs9937453 | 16 | 22155629 | VWA3A | 0.4252 | 0.5197 |
| rs12043211 | 1 | 14284460 |  | 0.4283 | 0.2983 |
| rs5919895 | X | 144853578 |  | 0.4285 | 0.3878 |
| rs10486733 | 7 | 43273124 | HECW1 | 0.4299 | 0.2371 |
| rs10214302 | 5 | 148238456 |  | 0.4319 | 0.5107 |
| rs10162699 | 15 | 82170051 |  | 0.4337 | 0.4215 |
| rs7206749 | 16 | 11376922 | TNP2,PRM3,PRM2,PRM1 | 0.4345 | 0.286 |
| rs11007320 | 10 | 29229683 |  | 0.4354 | 0.1807 |
| rs2420449 | 2 | 115573050 | DPP10 | 0.4357 | 0.395 |
| rs938059 | X | 66336727 |  | 0.4364 | 0.5028 |
| rs4818107 | 21 | 41383412 | DSCAM | 0.4375 | 0.4175 |
| rs697294 | 3 | 64217284 | PRICKLE2 | 0.4384 | 0.5015 |
| rs9885632 | 6 | 131311909 |  | 0.4393 | 0.9615 |
| rs11874912 | 18 | 12733705 | PSMG2 | 0.4397 | 0.4662 |
| rs7050299 | X | 116451603 |  | 0.4406 | 0.1432 |
| rs10910892 | 1 | 181160639 |  | 0.4419 | 0.4119 |
| rs13286134 | 9 | 76880794 |  | 0.4437 | 0.2813 |
| rs2970809 | 12 | 4564613 | FGF6 | 0.4442 | 0.4979 |
| rs872157 | 1 | 37716037 |  | 0.4458 | 0.5913 |
| rs12893005 | 14 | 21607930 |  | 0.446 | 0.5455 |
| rs6041093 | 20 | 12088568 |  | 0.4496 | 0.921 |
| rs2422697 | 20 | 2330615 | TGM3 | 0.4506 | 0.4669 |
| rs12817026 | 12 | 74402900 |  | 0.451 | 0.607 |
| rs17181389 | 11 | 44996996 |  | 0.4535 | 0.6804 |
| rs10876339 | 12 | 53161768 | LOC643898,KRT76 | 0.456 | 0.3999 |
| rs11696650 | 20 | 55378322 |  | 0.4567 | 0.3081 |
| rs10516853 | 4 | 90866819 | MMRN1 | 0.4576 | 0.5587 |
| rs285702 | 15 | 93116471 |  | 0.4581 | 0.4864 |
| rs360252 | 2 | 127012268 |  | 0.4588 | 0.7951 |
| rs985863 | 9 | 89219411 |  | 0.459 | 0.3297 |
| rs12680036 | 8 | 121947406 |  | 0.46 | 0.803 |
| rs9296562 | 6 | 47490193 | CD2AP | 0.4611 | 0.4334 |
| rs9864901 | 3 | 145522384 |  | 0.4612 | 0.472 |
| rs17133399 | 11 | 99193504 | CNTN5 | 0.4615 | 0.3354 |
| rs9369262 | 6 | 12332170 |  | 0.4658 | 0.3398 |
| rs301551 | 3 | 11798039 |  | 0.4663 | 0.4007 |
| rs17603726 | 4 | 37336553 | KIAA1239 | 0.4674 | 0.3682 |
| rs11611462 | 12 | 76211849 |  | 0.4674 | 0.5608 |
| rs788935 | 4 | 73379012 | ADAMTS3 | 0.4704 | 0.5139 |
| rs8137744 | 22 | 44697280 | KIAA1644 | 0.4714 | 0.4314 |
| rs9543740 | 13 | 75238833 |  | 0.4735 | 0.2912 |
| rs11674847 | 2 | 12922923 |  | 0.474 | 0.5666 |
| rs9489496 | 6 | 118997773 |  | 0.4742 | 0.5639 |
| rs7574829 | 2 | 115495142 | DPP10 | 0.4743 | 0.4531 |
| rs10888046 | 1 | 17979118 | ARHGEF10L | 0.4756 | 0.7295 |
| rs17341637 | X | 135718189 | RP3-527F8.2,CD40LG | 0.4784 | 0.6593 |
| rs2885478 | 2 | 234900234 | TRPM8 | 0.4785 | 0.3248 |
| rs6433105 | 2 | 169974671 | LRP2 | 0.4807 | 0.2533 |
| rs726934 | 10 | 105950609 | C10orf79 | 0.482 | 0.7646 |
| rs2971955 | 7 | 133665939 | EXOC4 | 0.4822 | 0.4574 |
| rs16985852 | 2 | 19242173 | FLJ41481 | 0.4823 | 0.6207 |
| rs6896002 | 5 | 21588931 | LOC100132788 | 0.484 | 0.3637 |
| rs13417114 | 2 | 72433296 | EXOC6B | 0.4849 | 0.5046 |
| rs9877486 | 3 | 60233087 | FHIT | 0.4872 | 0.4261 |
| rs10520924 | 5 | 25428287 |  | 0.4885 | 0.3808 |
| rs7077238 | 10 | 129683747 | CLRN3 | 0.4896 | 0.4043 |
| rs7910314 | 10 | 56004457 | PCDH15 | 0.4902 | 0.7566 |
| rs11734372 | 4 | 41673604 | LIMCH1 | 0.4931 | 0.6247 |
| rs10517450 | 4 | 59677511 |  | 0.4954 | 0.4608 |
| rs9639327 | 7 | 19340930 |  | 0.4957 | 0.9014 |
| rs10102717 | 8 | 19756813 |  | 0.4968 | 0.4 |
| rs578667 | 18 | 12498878 | SPIRE1 | 0.4971 | 0.5415 |
| rs264514 | 12 | 130167749 | TMEM132D | 0.4998 | 0.8337 |
| rs9949366 | 18 | 60690246 |  | 0.5027 | 0.3759 |
| rs360370 | 2 | 127118187 |  | 0.5062 | 0.7205 |
| rs12052647 | 2 | 4989067 |  | 0.5063 | 0.4382 |
| rs353465 | 5 | 176396888 | UIMC1 | 0.5065 | 0.8995 |
| rs1569748 | 1 | 84261598 |  | 0.5079 | 0.2003 |
| rs3762397 | 1 | 200090219 | NR5A2 | 0.508 | 0.2666 |
| rs11944669 | 4 | 22246755 |  | 0.512 | 0.4242 |
| rs11490542 | 19 | 6573967 | LOC100130584,RPL7P50 | 0.5151 | 0.5579 |
| rs9995630 | 4 | 161427827 |  | 0.5183 | 0.7495 |
| rs11739623 | 5 | 131864152 |  | 0.52 | 0.5024 |
| rs10756018 | 9 | 10254669 | PTPRD | 0.5216 | 0.5212 |
| rs2527753 | 8 | 5400784 |  | 0.5222 | 0.3416 |
| rs1062492 | 7 | 2290685 | MAD1L1,FTSJ2,NUDT1,SNX8,LOC727926 | 0.5252 | 0.5249 |
| rs6600143 | 16 | 201389 | C16orf35,HBZ,HBZP,HBM,HBAP1 | 0.5265 | 0.5316 |
| rs12541759 | 8 | 88827181 |  | 0.5284 | 0.994 |
| rs11062885 | 12 | 3978661 | PARP11 | 0.5298 | 0.3562 |
| rs4830593 | X | 8567981 | KAL1 | 0.5323 | 0.6405 |
| rs12069748 | 1 | 241843408 | WDR64 | 0.5328 | 0.5436 |
| rs12099330 | 11 | 20009559 | NAV2 | 0.5328 | 0.6416 |
| rs4574919 | 9 | 113501372 | MUSK | 0.5338 | 0.4656 |
| rs807418 | 4 | 12808091 |  | 0.5342 | 0.4891 |
| rs4243218 | 16 | 81900628 | PLCG2 | 0.5368 | 0.6367 |
| rs11525791 | 7 | 14491762 | DGKB | 0.5369 | 0.2176 |
| rs7971738 | 12 | 63896181 |  | 0.5397 | 0.5739 |
| rs11851053 | 14 | 105407208 | PLD4,AHNAK2 | 0.5407 | 0.3539 |
| rs2618390 | 3 | 12047854 | SYN2 | 0.5423 | 0.2047 |
| rs6018056 | 20 | 45427300 |  | 0.5427 | 0.5338 |
| rs2826487 | 21 | 22084693 |  | 0.5437 | 0.5721 |
| rs11232180 | 11 | 80236788 |  | 0.5474 | 0.6011 |
| rs919581 | 7 | 136980174 | PTN | 0.5483 | 0.4429 |
| rs17721983 | 3 | 71855936 |  | 0.5492 | 0.5604 |
| rs4580824 | 6 | 80120201 |  | 0.5497 | 0.5538 |
| rs6869938 | 5 | 31676987 |  | 0.5539 | 0.426 |
| rs4089885 | X | 23118388 |  | 0.5564 | 0.5366 |
| rs2662985 | 20 | 8397222 | PLCB1 | 0.5589 | 0.5119 |
| rs1446445 | 2 | 53256694 |  | 0.5589 | 0.6127 |
| rs204590 | 7 | 20907104 |  | 0.5589 | 0.7005 |
| rs2397327 | 10 | 3691304 |  | 0.5597 | 0.6602 |
| rs1418313 | 6 | 22516794 |  | 0.5597 | 0.6849 |
| rs10026106 | 4 | 61632982 |  | 0.5619 | 0.4111 |
| rs4946673 | 6 | 106000830 |  | 0.5651 | 0.3147 |
| rs10866820 | 8 | 22977170 | TNFRSF10C | 0.5664 | 0.3005 |
| rs9853619 | 3 | 130024270 |  | 0.568 | 0.7365 |
| rs942127 | 1 | 59284219 | LOC100131060 | 0.5695 | 0.9358 |
| rs16979600 | 19 | 45487420 | CLPTM1,RELB | 0.57 | 0.7488 |
| rs12605240 | 18 | 12591483 | SPIRE1 | 0.5727 | 0.481 |
| rs1391997 | 13 | 69127905 |  | 0.5728 | 0.5521 |
| rs6826407 | 4 | 89975033 | FAM13A | 0.5753 | 0.433 |
| rs3794716 | 17 | 80559167 | FOXK2 | 0.5757 | 0.3824 |
| rs3850153 | X | 3167102 | CXorf28 | 0.5777 | 0.9077 |
| rs6534331 | 4 | 122924339 |  | 0.5795 | 0.7575 |
| rs2653312 | 3 | 187264516 |  | 0.5822 | 0.6056 |
| rs212621 | 16 | 16269962 | ABCC6 | 0.5839 | 0.3201 |
| rs6720427 | 2 | 2527641 |  | 0.5857 | 0.3235 |
| rs1872277 | 11 | 45058125 |  | 0.5894 | 0.6378 |
| rs1993776 | 13 | 55508485 |  | 0.5913 | 0.5244 |
| rs443186 | 19 | 36345951 | NPHS1,KIRREL2,APLP1 | 0.5947 | 0.4936 |
| rs16901189 | 5 | 72066542 |  | 0.5971 | 0.8526 |
| rs11072416 | 15 | 33656712 | RYR3 | 0.5995 | 0.512 |
| rs17135375 | 5 | 112507046 | MCC | 0.6016 | 0.6901 |
| rs245136 | 5 | 11229362 | CTNND2 | 0.6016 | 0.8029 |
| rs2846186 | 11 | 134698376 |  | 0.6026 | 0.5833 |
| rs5029676 | 10 | 87189235 |  | 0.6029 | 0.5293 |
| rs2371290 | 14 | 80883422 |  | 0.604 | 0.5147 |
| rs16851066 | 3 | 140842821 | SPSB4 | 0.6077 | 0.5982 |
| rs1538001 | 13 | 34785068 |  | 0.6087 | 0.7578 |
| rs1440459 | 7 | 154849853 | LOC100128264,HTR5A | 0.6092 | 0.4935 |
| rs4552808 | 7 | 26607584 |  | 0.6107 | 0.9178 |
| rs17162010 | 7 | 9807756 | tcag7.893 | 0.6125 | 0.5326 |
| rs333548 | 15 | 66245497 | MEGF11 | 0.6214 | 0.4965 |
| rs3770244 | 2 | 45633100 | SRBD1 | 0.6246 | 0.8669 |
| rs736693 | 4 | 119847321 | SYNPO2 | 0.6287 | 0.9884 |
| rs1450949 | 5 | 51919136 |  | 0.6329 | 0.9656 |
| rs10795206 | 10 | 4909968 | tAKR | 0.6344 | 0.6479 |
| rs12716256 | 5 | 169962051 | KCNIP1 | 0.6351 | 0.616 |
| rs488196 | 19 | 56677299 | ZNF444,GALP | 0.6354 | 0.7378 |
| rs851860 | 6 | 67054884 |  | 0.6354 | 0.9189 |
| rs855365 | 6 | 9834529 |  | 0.6375 | 0.5831 |
| rs33713 | 5 | 87512314 | TMEM161B | 0.6398 | 0.9055 |
| rs11250938 | 10 | 1984431 |  | 0.6406 | 0.5216 |
| rs7111184 | 11 | 120771063 | GRIK4 | 0.6413 | 0.413 |
| rs6025653 | 20 | 56180563 | ZBP1 | 0.6436 | 0.712 |
| rs4815586 | 20 | 389984 | RBCK1 | 0.6438 | 0.8846 |
| rs9804196 | 10 | 83796220 | NRG3 | 0.6445 | 0.2959 |
| rs5910001 | X | 122509293 | GRIA3 | 0.6467 | 0.5737 |
| rs11082752 | 18 | 46968603 | DYM | 0.6494 | 0.5686 |
| rs509497 | 18 | 6957193 | LAMA1 | 0.6523 | 0.71 |
| rs17119158 | 14 | 84334011 |  | 0.656 | 0.462 |
| rs3845972 | 3 | 60027834 | FHIT | 0.6564 | 0.648 |
| rs1393824 | 4 | 109171067 | LOC644993 | 0.66 | 0.9819 |
| rs4098 | 3 | 156501063 |  | 0.6601 | 0.2164 |
| rs26039 | 5 | 11219938 | CTNND2 | 0.6643 | 0.777 |
| rs11029100 | 11 | 26047452 |  | 0.6646 | 0.4513 |
| rs16964761 | 13 | 105086976 |  | 0.665 | 0.9311 |
| rs6553726 | 4 | 174682766 |  | 0.6663 | 0.5899 |
| rs10903603 | 10 | 1981965 |  | 0.6711 | 0.5675 |
| rs2048451 | 5 | 90567208 | LOC729040 | 0.6726 | 0.8305 |
| rs7455326 | 7 | 121148166 | RPL18P4 | 0.6741 | 0.6402 |
| rs17245882 | X | 145672737 |  | 0.6788 | 0.6127 |
| rs5971552 | X | 31230867 | DMD | 0.6793 | 0.5952 |
| rs10058487 | 5 | 24195807 |  | 0.6802 | 0.4036 |
| rs17790020 | 8 | 62419877 | CLVS1,ASPH | 0.6843 | 0.657 |
| rs563097 | 8 | 26717123 | ADRA1A,LOC100127897 | 0.6844 | 0.7767 |
| rs883743 | 6 | 4522240 |  | 0.6864 | 0.6945 |
| rs13379043 | 14 | 74250126 | C14orf43 | 0.6875 | 0.9104 |
| rs926767 | 14 | 29202736 |  | 0.6877 | 0.932 |
| rs10938882 | 4 | 22235324 |  | 0.6894 | 0.5689 |
| rs2636860 | 9 | 117030582 | COL27A1 | 0.691 | 0.6821 |
| rs2431367 | 5 | 92138100 |  | 0.6917 | 0.8049 |
| rs17276873 | 9 | 125253806 | OR1J1,OR1J2 | 0.6933 | 0.728 |
| rs9820239 | 3 | 41372090 | ULK4 | 0.6954 | 0.6524 |
| rs6517436 | 21 | 39223338 | KCNJ6 | 0.7021 | 0.5344 |
| rs253594 | 5 | 155970607 | SGCD | 0.7034 | 0.8264 |
| rs1475720 | 13 | 82301380 |  | 0.7043 | 0.8888 |
| rs151228 | 16 | 28563026 | NUPR1,CCDC101 | 0.7044 | 0.6978 |
| rs11142684 | 9 | 73691183 | TRPM3 | 0.7083 | 0.4924 |
| rs1441772 | 8 | 19753602 |  | 0.7086 | 0.619 |
| rs1368898 | 2 | 80735034 | CTNNA2 | 0.7087 | 0.6826 |
| rs3827223 | 21 | 43338175 | C2CD2 | 0.7103 | 0.871 |
| rs1396414 | 3 | 3496478 |  | 0.7105 | 0.31 |
| rs2815634 | 10 | 12589764 | CAMK1D | 0.7111 | 0.5377 |
| rs9299186 | 9 | 113319332 | SVEP1 | 0.7115 | 0.7082 |
| rs1078684 | 18 | 65953119 |  | 0.7163 | 0.4808 |
| rs9405302 | 6 | 6477849 |  | 0.7187 | 0.9642 |
| rs4714791 | 6 | 44441440 | LOC100128935 | 0.72 | 0.9315 |
| rs7186907 | 16 | 52527403 | TOX3 | 0.721 | 0.9384 |
| rs10743735 | 12 | 30971860 |  | 0.7217 | 0.6708 |
| rs1523936 | 6 | 69605682 | BAI3 | 0.722 | 0.4921 |
| rs11684559 | 2 | 174401638 |  | 0.7221 | 0.853 |
| rs4371706 | 5 | 51185244 |  | 0.7292 | 0.6275 |
| rs4407260 | 2 | 165236635 |  | 0.7294 | 0.7874 |
| rs6489498 | 12 | 3902269 |  | 0.7308 | 0.5248 |
| rs10057772 | 5 | 111976736 |  | 0.7309 | 0.7964 |
| rs9545162 | 13 | 36304734 |  | 0.7323 | 0.6724 |
| rs7110848 | 11 | 123812614 | OR4D5,OR6T1 | 0.7334 | 0.7769 |
| rs4864619 | 4 | 58178214 |  | 0.7352 | 0.5716 |
| rs4901141 | 14 | 52084533 | FRMD6 | 0.7369 | 0.5105 |
| rs12769490 | 10 | 106115547 | CCDC147 | 0.737 | 0.8148 |
| rs966645 | 10 | 106010539 | C10orf79,GSTO1,GSTO2 | 0.7371 | 0.7673 |
| rs17158744 | 7 | 111353361 |  | 0.7381 | 0.7267 |
| rs7700519 | 5 | 4708616 |  | 0.7391 | 0.5908 |
| rs4241997 | 4 | 56588964 |  | 0.7407 | 0.8065 |
| rs9783924 | 18 | 22010421 | IMPACT | 0.7491 | 0.41 |
| rs4904145 | 14 | 84338200 |  | 0.7515 | 0.8675 |
| rs1975080 | 18 | 21225533 | ANKRD29 | 0.7556 | 0.8414 |
| rs6082968 | 20 | 2352590 | TGM6 | 0.7559 | 0.4947 |
| rs4539728 | 19 | 19431480 | SF4,KIAA0892 | 0.7573 | 0.9344 |
| rs11111443 | 12 | 103495380 | LOC644171 | 0.7599 | 0.947 |
| rs1792366 | 11 | 92449367 | FAT3 | 0.7616 | 0.6555 |
| rs870842 | 20 | 9909366 |  | 0.764 | 0.5914 |
| rs11025754 | 11 | 20798999 | NELL1 | 0.7753 | 0.6591 |
| rs6953748 | 7 | 158121184 | PTPRN2 | 0.7805 | 0.6344 |
| rs926685 | 1 | 84267412 |  | 0.7819 | 0.5239 |
| rs2340412 | X | 139580982 | SOX3,RP1-177G6.2 | 0.7843 | 0.6673 |
| rs4509693 | 10 | 102501571 | PAX2 | 0.7854 | 0.9795 |
| rs1407913 | 9 | 10228955 | PTPRD | 0.7859 | 0.8918 |
| rs1498609 | 5 | 58344518 | PDE4D,LOC642781 | 0.7863 | 0.6649 |
| rs2147639 | 13 | 55581949 |  | 0.7896 | 0.555 |
| rs1024350 | 14 | 107141122 | IGHV3-65,IGHVII-65-1,IGHV3-66,IGHV1-67,LOC192127,IGHVII-67-1,IGHVIII-67-2,IGHVIII-67-3,IGHVIII-67-4 | 0.7932 | 0.7911 |
| rs2919996 | 18 | 28769637 |  | 0.7967 | 0.6507 |
| rs11134043 | 5 | 4285241 |  | 0.7976 | 0.8554 |
| rs5753158 | 22 | 30855697 | SEC14L3 | 0.8045 | 0.8289 |
| rs3750249 | 8 | 17079358 | ZDHHC2,CNOT7 | 0.8081 | 0.5067 |
| rs6528069 | X | 21915630 |  | 0.8084 | 0.8959 |
| rs1513410 | 3 | 145566063 |  | 0.8092 | 0.7832 |
| rs7129006 | 11 | 22803712 | GAS2 | 0.8094 | 0.7746 |
| rs510386 | 9 | 138560560 | LCN9 | 0.8125 | 0.6205 |
| rs1343567 | 9 | 14627829 | ZDHHC21 | 0.8157 | 0.6732 |
| rs11775611 | 8 | 52820274 |  | 0.8204 | 0.9836 |
| rs17268691 | 5 | 65913576 | MAST4,LOC100129571 | 0.8205 | 0.9983 |
| rs7986393 | 13 | 25930658 | LOC246717,ATP8A2 | 0.8215 | 0.6078 |
| rs9561537 | 13 | 94975867 | GPC6 | 0.8227 | 0.8013 |
| rs4844078 | X | 148066042 | AFF2 | 0.828 | 0.5809 |
| rs6565851 | 18 | 73887392 |  | 0.8315 | 0.7499 |
| rs7161558 | 14 | 31698164 |  | 0.8357 | 0.8551 |
| rs4937181 | 11 | 126611183 | KIRREL3 | 0.8369 | 0.5934 |
| rs8049355 | 16 | 12776837 | CPPED1 | 0.8403 | 0.9613 |
| rs11651535 | 17 | 36178309 |  | 0.8415 | 0.6522 |
| rs9650706 | 9 | 386094 | DOCK8 | 0.8421 | 0.9333 |
| rs2895215 | 7 | 1837636 |  | 0.8434 | 0.5327 |
| rs17767591 | 17 | 69795788 |  | 0.8465 | 0.8622 |
| rs7523134 | 1 | 58899674 | MYSM1 | 0.8495 | 0.8229 |
| rs7327131 | 13 | 73801428 |  | 0.8502 | 0.8177 |
| rs12587414 | 14 | 94287575 |  | 0.8513 | 0.9981 |
| rs11688532 | 2 | 128666689 |  | 0.8522 | 0.9765 |
| rs17124619 | 20 | 31934178 |  | 0.8546 | 0.9643 |
| rs12024346 | 1 | 195995948 |  | 0.855 | 0.613 |
| rs7841191 | 8 | 83405148 |  | 0.8564 | 0.6059 |
| rs537148 | 8 | 12922112 |  | 0.8575 | 0.856 |
| rs6650282 | 13 | 95748221 | ABCC4 | 0.8616 | 0.9434 |
| rs17806455 | 3 | 20501468 |  | 0.8681 | 0.7824 |
| rs1962914 | 18 | 3426898 | TGIF1 | 0.8733 | 0.934 |
| rs4759551 | 12 | 131640617 | LOC116437 | 0.8746 | 0.7293 |
| rs2171778 | 4 | 38936833 | FAM114A1 | 0.8786 | 0.9487 |
| rs7895964 | 10 | 4002033 |  | 0.8834 | 0.681 |
| rs4341651 | 13 | 54586637 |  | 0.8851 | 0.8164 |
| rs7616178 | 3 | 189433127 | TP63 | 0.887 | 0.9314 |
| rs2042566 | 2 | 12878668 | TRIB2 | 0.8908 | 0.9405 |
| rs1035803 | 4 | 146997376 |  | 0.8992 | 0.9251 |
| rs10517919 | 4 | 167962448 | SPOCK3 | 0.8993 | 0.9736 |
| rs10947982 | 6 | 12384972 |  | 0.9012 | 0.8416 |
| rs7434469 | 4 | 6410746 | PPP2R2C | 0.9024 | 0.9011 |
| rs4766521 | 12 | 111386961 |  | 0.9041 | 0.9685 |
| rs11692282 | 2 | 126759423 |  | 0.9095 | 0.7129 |
| rs13432312 | 2 | 38490324 |  | 0.9117 | 0.7723 |
| rs12881336 | 14 | 57843487 | NAA30 | 0.9126 | 0.9052 |
| rs11103218 | 9 | 138813713 |  | 0.9131 | 0.677 |
| rs10514030 | 18 | 69754612 |  | 0.9243 | 0.9027 |
| rs7972295 | 12 | 43769718 | ADAMTS20 | 0.9249 | 0.9662 |
| rs1383782 | 14 | 85110699 |  | 0.9263 | 0.892 |
| rs2324027 | 17 | 15679601 | LOC100130110,IL6STP,MEIS3P1 | 0.9271 | 0.7829 |
| rs4344939 | 2 | 18839700 |  | 0.9319 | 0.8387 |
| rs17712592 | 5 | 158173509 | EBF1 | 0.9359 | 0.912 |
| rs16953047 | 16 | 54130170 | FTO | 0.9399 | 0.7723 |
| rs6970352 | 7 | 21323151 |  | 0.9463 | 0.8587 |
| rs83520 | 8 | 140720412 | KCNK9 | 0.9476 | 0.68 |
| rs17113393 | 14 | 43200986 |  | 0.9481 | 0.8664 |
| rs10961640 | 9 | 14662171 | ZDHHC21 | 0.9511 | 0.8473 |
| rs2081776 | 2 | 145080938 |  | 0.953 | 0.9117 |
| rs12422191 | 11 | 113274010 | ANKK1,DRD2 | 0.9566 | 0.7542 |
| rs10496483 | 2 | 115964510 | DPP10 | 0.9579 | 0.9396 |
| rs3110430 | 8 | 107606599 |  | 0.9629 | 0.8656 |
| rs9946373 | 18 | 3962377 |  | 0.9648 | 0.9096 |
| rs263836 | 13 | 55557872 |  | 0.965 | 0.8473 |
| rs2731826 | 5 | 94890124 | TTC37,ARSK | 0.9676 | 0.8769 |
| rs7658861 | 4 | 165305889 |  | 0.9729 | 0.8163 |
| rs2487783 | 13 | 60847318 | LOC440142 | 0.9731 | 0.9945 |
| rs1507358 | 1 | 212925361 | NSL1 | 0.9752 | 0.7957 |
| rs2298703 | 11 | 113134605 | NCAM1 | 0.9754 | 0.9739 |
| rs10885557 | 10 | 116114636 | AFAP1L2 | 0.9762 | 0.8738 |
| rs7144510 | 14 | 55940193 |  | 0.9775 | 0.7874 |
| rs557135 | 6 | 52765760 | GSTA3 | 0.9779 | 0.9788 |
| rs10506475 | 12 | 66498118 |  | 0.9782 | 0.7035 |
| rs10176156 | 2 | 171230560 | MYO3B | 0.9859 | 0.8006 |
| rs4828054 | X | 99997406 | SYTL4 | 0.9862 | 0.9889 |
| rs10276315 | 7 | 68037870 |  | 0.9932 | 0.8336 |
| rs2137002 | 14 | 84417927 |  | 0.9944 | 0.8817 |
| rs13396519 | 2 | 11040146 | KCNF1 | 0.9967 | 0.6719 |
