# Supplementary material for: Genome-wide association study of endometrial cancer in E2C2
Source: Hum Genet. 2013 Oct 6;133(2):211–24. doi: 10.1007/s00439-013-1369-1 (PMC3898362; doi:10.1007/s00439-013-1369-1)
Supplement: Supplementary file 5 — Supplementary material 5 (DOCX 16 kb) [file 439_2013_1369_MOESM5_ESM.docx]

Supplementary Table 5: Racial and ethnic distribution among replication studies.

|  | CPSII | Turin | WISE | AHS | EDGE | FHCRC | MEC | ANECS/SEARCH/  QIMR/HCS/WTCC2 | SECGS |
| --- | --- | --- | --- | --- | --- | --- | --- | --- | --- |
| Race/Ethnicity |  |  |  |  |  |  |  |  |  |
| White | 993 | 539 | 929 | 1358 | 451 |  |  | 9,560 |  |
| Black |  |  | 193 |  | 27 | 23 | 212 |  |  |
| Asian |  |  |  |  |  |  | 366 |  | 2,770 |
| Hispanic |  |  |  |  | 18 | 46 | 318 |  |  |
| Hawaiian |  |  |  |  |  |  | 81 |  |  |
